# The Relation Between Consumers' Frontal Alpha Asymmetry, Attitude, and Investment Decision

**DOI:** 10.3389/fnins.2020.577978

**Published:** 2021-01-21

**Authors:** Francesco Di Gruttola, Andrea P. Malizia, Sonia D'Arcangelo, Nicola Lattanzi, Emiliano Ricciardi, Maria Donata Orfei

**Affiliations:** ^1^IMT School for Advanced Studies Lucca, Lucca, Italy; ^2^Intesa Sanpaolo Innovation Center SpA, Neuroscience Lab, Torino, Italy

**Keywords:** frontal alpha asymmetry (FAA), EEG-electroencephalogram, decision-making -investments, consumer attitude, pupil diameter, consumer choice

## Abstract

The frontal alpha asymmetry (FAA) is a neurophysiological measure of motivation and preference. Despite the FAA is associated to commercial pleasantness, conflicting evidence emerged in the literature regarding its relationship with behavior. To study the association between FAA and consumers' decision, we manipulated a commercial script to elicit diverse consumers' attitudes and decisions and to evaluate whether the FAA score is associated to their final investment. A little informative script (S1) was used to polarize consumers' attitudes and investments toward unfavorable scores, while a more personalized message (S2) to elicit in customers a favorable attitude and higher investments. Twenty-one participants listened to the scripts, and their FAA, attitude, and monetary investment were measured. In S1, the FAA did not correlate with neither attitude nor the investment decision, while a robust negative correlation between these variables was found in S2. No other peripheral body and neural measures associated with attitude or final decision. Our data suggest that the FAA correlates with attitude and decision, when a commercial script is customized and provides an adequate information, likely leading the consumer to a more reasoned and planned decision-making process. When facilitating a favorable attitude toward an offer, the negative correlation of FAA and behavior may reflect the involvement of a control system, whose role is to monitor and govern possible conflicts between approach and avoidance motivations. This observation provides additional indication on the value of FAA as a marker of consumer behaviors, and how it could be affected by experimental and contextual bias.

## Introduction

So far, research in Consumer Neuroscience developed different approaches to determine consumers' attitude toward a given commercial message—i.e., the tendency to respond to a stimulus with some degree of favorableness or unfavorableness (Ajzen, 2008)—and their final choice, through behavioral, body peripheral, or neural measures (Vecchiato et al., 2014; Cherubino et al., 2019). An index that specifically measures consumers' motivation toward the stimulus and reflects their intention and volition is the frontal alpha asymmetry (FAA—for a comprehensive review, see Hewig, 2017; Hakim and Levy, 2018). The FAA is computed as the difference between the frontal right and left hemispheres in the alpha band spectra (Hakim and Levy, 2018). Typically, the frontal Alpha rhythm (8–12 Hz) is linked to brain functions related to information processing, attention, decision-making, and emotion regulation (Cohen et al., 2009; Klimesch, 2012; Zhang et al., 2018; Misselhorn et al., 2019); high alpha power has been related to inhibitory control, while low power to neural activation (Larson et al., 1998; Benedek et al., 2011). To date, this neural marker has been interpreted to reflect a motivational direction and preference toward the stimulus (regardless of the experienced positive or negative affective valence) that occurs just prior to the behavior outcome (Harmon-Jones et al., 2010; Hewig, 2017). As such, Vecchiato et al. (2011) measured the FAA during the presentation of different TV commercials and reported a relation between participants' ratings of the pleasantness of the video clips and the frontal asymmetry. A great left hemispheric activity in the alpha domain has been interpreted as an approach motivation, while a right may indicate an avoidance drive from the stimulus. This interpretation has been confirmed in other investigations (Coan and Allen, 2003; Hakim and Levy, 2018). However, although the evidence in favour of this classical interpretation is widely documented, alternative perspectives on the FAA are emerging in the literature. In this vein, a review by Gable et al. (2017) pointed out that, although the relationship between left frontal activity and the approach motivation results more solid, defined as the behavioral approach system (BAS), the findings in support of a relationship between a right frontal activity and the avoidance motivation are mixed, and further research are needed.

Despite the association between FAA and the pleasantness of the stimulus is widely documented, the extent to which FAA is also able to predict consumers' attitude and final decision is still a matter of debate. To this aim, Cherubino et al. (2019) underlined the importance of unveiling brain processes and behaviors that are not possible to observe with self-reported measures (e.g., questionnaires) and to boost the research for physiological markers able to predict consumers' attitude and choices. The FAA, being an index of consumers' motivation toward the stimulus (i.e., a commercial offer) could be a possible candidate. However, few studies—and with mixed observations—investigated the association between consumers' FAA and both the attitude and their final choice. Purposely, Fischer et al. (2018) highlighted how the FAA was a reliable predictor and a potential neural marker of the online information-sharing behavior that is an indirect measure of final consumer decision. Similarly, Ravaja et al. (2013) found that a high activation of the left frontal cortex (approach motivation) during the predecisional phase, predicted the decision to purchase grocery products with different prices. On the contrary, Ramsøy et al. (2018) asked potential consumers how much they would have paid for some commercial products (i.e., willingness to pay) and did not find an association between FAA and consumers' decision, thus challenging the assumption that the FAA may be related to customers' final choice. Accordingly, a well-defined correlation between FAA and both consumers' attitude and final decision is still missing, especially when considering different contextual factors, or diverse processes by which the decision is made.

In this context, to better characterize this relation, we could rely on the overt manipulation of the stimuli (i.e., commercial offers) both to elicit diverse attitudes of disfavor and favor in the customers and, consequently, to impact consumers' decision (i.e., different willingness to pay). Indeed, the modulation of a commercial through the amount of provided information and the personalization of the message may have a significant influence on consumers' attitudes and final choice (Hahn et al., 1992; Coulter et al., 2001; Goldsmith and Freiden, 2004; Xu, 2006; Ünal et al., 2011; Bostrom et al., 2013; Boerman et al., 2017; Gironda and Korgaonkar, 2018), with a possible impact also on the FAA and on the consequent relation between these variables. Specifically, when information is limited, customers hardly undertake an in-depth commercial evaluation and an accurate final decision; conversely, when the information load increases, the capacity for information processing and the consistency of decision-making increase (Hahn et al., 1992). Moreover, a more informative message may stimulate consumers toward a more positive attitude (Coulter et al., 2001; Ünal et al., 2011). Equally, after a careful evaluation of individual past behaviors, needs, and interests, the presentation of tailored commercial potentially prompts a general positive attitude on consumers' intentions and choices (Goldsmith and Freiden, 2004; Xu, 2006; Bostrom et al., 2013).

Consequently, through the manipulation of a commercial offer to induce diverse consumers' attitudes and investment decisions, the present study aimed at assessing whether the FAA score correlates to consumers' attitude and final decision. According to our experimental modulation, we developed two fictitious commercial offers. In the first frame, we presented a commercial call, limited in the amount of information and formulated in a general standard message. This type of call was expected to elicit an unfavorable and polarized attitude in participants and stimulate them to invest a small and less variable amount of money. Conversely, in a second frame, we presented a highly informative and personalized message compared with the first frame that was expected to produce a more favorable and less polarized attitude and a larger and more variable amount of money invested than the other condition. Given this experimental manipulation, we investigated the correlation between the FAA and consumers' attitudes and decisions (i.e., the percentage of investment), and also compared this neural marker with other control measures (e.g., the pupil diameter), to verify whether the FAA would specifically reflect consumers' intention and volition other than a general index of information processing and attention.

## Materials and Methods

### Participants

We recruited 21 native Italian and right-handed participants (11 females; mean age ± SD: 38.4 ± 11.3 years) by means of the Edinburgh Handedness Inventory (Oldfield, 1971). Participants had normal/corrected-to-normal vision, no history of auditory or psychiatric disorders, and were selected from a pool of volunteers at the IMT School for Advanced Studies Lucca whose age range (25–60) matched the representative target population of customers for the insurance policy used in the experiment. The study was made in accordance with the ethical principles of the Declaration of Helsinki (World Medical Association, 2013). Participants were provided with an exhaustive description of all the experimental procedures and were required to sign a written informed consent. The study was conducted under a protocol approved by the Area Vasta Nord Ovest Ethics Committee (protocol n. 24579/2018).

### Experimental Design

The experiment was conducted during weekdays' mornings (10–12 a.m.) and afternoons (2–6 p.m.). After reading and compiling the informed consent, participants sat comfortably in a soundproofed and shielded box. The room was illuminated exclusively with artificial lights. Accordingly, the brightness of the room was maintained stable and equal for all the participants in the experiment. They were in front of a table on which there were a computer screen and audio speakers used for the projection and reproduction of experimental stimuli. The investigators controlled the experimental protocol being outside the shielded box, and they were in contact with the participants through a microphone.

Firstly, participants were asked to perform a gambling game in which 15 matrices adapted from the Raven Matrices test were shown (Raven et al., 1998). The stimuli were presented by means of the PowerPoint software controlled by the experimenters. Participants had to complete each figure choosing one from six possible missing pieces. Before the choice, participants bet a fictitious amount of money using a real-like monetary system (1,000, 5,000, 10,000, and 20,000 Italian Lire) based on how much they felt confident in providing the correct answer. If participants guessed the answer, they won the amount bet, otherwise, they won nothing. Participants' answers were manually recorded on a paper grid by the experimenter. At the end of the gambling game, participants collected a virtual amount of money given by the sum of the bets won. Eventually, participants had to use this virtual wallet to be invested in each of the commercial offers presented later.

After this phase, participants were fitted with an EEG cap (Electrical Geodesic, 64 electrodes) suitable for their head circumference and an eye-tracker device (Tobii Pro Glasses 2). Then, the experimental protocol was administered by using the E-Prime software (version 2)^1^. The EEG signal and the pupil diameter were recorded throughout the duration of the protocol. After reading the instructions, a black screen with a white fixation cross was projected for 5 s (rest phase). Then a first commercial call started. Script 1 (S1) lasted 51 s. In this frame, the consultant claimed to have selected just 50 clients in order to propose new insurance policies at advantageous conditions. A few general details were provided on the policy, namely that the modules of the policy were adaptable to specific personal needs and the offer was valid only in the current month. Participants were instructed to listen to the entire audio script while continuing to stare the fixation cross, limiting body movements to a minimum and their interaction with the voice. The same pattern was performed also for the second commercial call. Script 2 (S2) lasted 87 s. In this frame, the consultant claimed that the offer was tailored according to participants' specific needs because their personal profiles were examined. Three main pieces of information were provided to the customers: the name of the insurance policy, the presence of different modules that were adaptable according to personal needs by making some practical example (e.g., work, family, etc.), and the presence of a progressive discount related to the purchase of multiple modules.

Accordingly, the two commercial scripts differed in the specificity of the product (i.e., insurance) details and the customization of the offer, so that the commercial S2 resulted to be more detailed and personalized, according to our experimental modulation aims. The transcripts of each script in Italian and translated into English language are attached in the Supplementary Material 1. Each commercial offer was presented once (one trial) to participants. Both of the audio scripts were recorded *ad hoc* by one of the experimenters. We used a female voice that acted as a fictitious consultant. A male actor was not used, supported by the evidence that the voice gender does not have a significant impact on advertising effectiveness (Rodero et al., 2012). The scripts were presented in the Italian language and differed in the duration, number of information, and personalization of the message provided by the fictitious consultant about the insurance policy. The PC screen brightness was maintained at the same level across participants both in baseline and experimental conditions because we presented the same black screen with a central white fixation cross.

After the end of each audio script, participants were asked to answer four closed questions which measured participants' attitude of disfavor and favor toward the offer with two response options and how much of the virtual wallet previously collected in the gambling game they wanted to invest in the just-presented offer. Participants answered directly by voice to the investigator by means of a microphone and without any time constraints. The experimenters manually collected the answers on a paper grid. The presentation of the two scripts was counterbalanced across participants in order to avoid possible problems and confounds derived from the presentation order. The start and the end of each event of the protocol, such as the rest and the audio script listening, were recorded through a parallel port by the EEG acquisition software.

### Measures

#### Sentiment Analysis

To check whether the traces did not use words that could evoke an *a priori* difference in participants' emotional valence that could account for both the attitude toward the stimulus and the EEG responses, we made a sentiment analysis on the English translation of each script in Python (version 3.8)^2^ by means of the vederSentiment (version 3.3.2)^3^ package. The vederSentiment classification score proved a large correlation with other well-established sentiment analysis indexes that include also the use of social words in their scores (i.e., the Linguistic Inquiry Word Count—Hutto and Gilbert, 2015). For each commercial offer, the algorithm divided the lexicon in sentences by means of the spaCy (version 2.3.2)^4^ package. For each sentence in each commercial offer, a normalized weighted composite valence index called compound score was obtained (Hutto and Gilbert, 2015). For each commercial offer, a whole compound score was computed by averaging the scores obtained in each sentence. The compound score ranged from −1 (most extreme negative) to 1 (most extreme positive—Hutto and Gilbert, 2015). The algorithm that we produced for the sentiment analysis is attached in the Supplementary Material 2.

#### Attitude Toward the Offer

For each condition and participant, the answers collected to the questions made at the end of the commercial offer were transformed in a metric scale: (a) Do you think the offer is disadvantageous or advantageous for you? (bias toward the offer, yes = 1, no = 2); (b) Do you think that the information in the offer was inadequate (vague/excessive) or adequate? (opinion on the adequacy of information, inadequate = 1, adequate = 2); (c) Did the personalized features of the offer give you a negative or positive impression? (opinion on the personalization of the offer, negative impression = 1, positive impression = 2); (d) Do you reject or accept to schedule a meeting with the consultant? (decision on planning a meeting with the consultant, reject = 1, accept = 2). Then, for each condition and participant, we summed up these scores and computed a whole index of attitude toward the offer, ranging from 4 (highest disfavor) to 8 (highest favor). We computed a whole score instead of using each single variable for the analysis given the small sample size and because this approach limits the number of variables to be analyzed and the multiple comparison correction in the statistical tests.

#### Monetary Investment

For each participant and condition, the monetary investment (%) was calculated according to the percentage formula, where participants' willingness to pay was divided by the total amount of money available won in the gambling game. We computed the percentage of investment score separately for each participant and each offer, without averaging the score across subjects or conditions. With this operation, we normalized the willingness to pay and made the scores comparable across participants.

#### Pupil Diameter

The pupil diameter (measured in mm) of the left and right eyes was obtained with a sampling rate of 100 Hz. For each participant, condition, and eye, the signal in the data segments between −5,000 ms (the onset of the rest phase) and −200 ms before the starting of the audio script, were considered baseline, while from 0 ms (the onset of the audio script) to the end of the commercial offer as the task. Because we only recorded one trial both for the baseline and the task, for each participant, condition, and eye, both the baseline and the trial were divided into two epochs of equal length. This operation contributed to make a separate preprocessing of the signal within the initial and final period of the trial. Then, each epoch was preprocessed by means of the CHAP^5^ toolbox of MATLAB (Version R2018b)^6^ (Hershman et al., 2019). The CHAP software merged the pupil diameter of both eyes together and computed the z scores according to the mean and SD of each epoch. For each epoch, the data points above and below 3 SD were considered outliers and excluded from the analysis. Then, the blinks were identified and excluded by the CHAP algorithm and the pupil size of all the missing values (e.g., blinks, outliers, or other missing) was reconstructed by means of the linear interpolation. For each condition, the mean pupil diameter of each epoch (initial and final period) was computed and averaged within the baseline and the task data segments. Then, for each condition, we computed the relative change of pupil diameter by subtracting the mean pupil diameter of the task with that of the baseline (Hershman et al., 2019). Accordingly, positive and negative values corresponded to a high and low pupil diameter changes with respect to the baseline.

#### EEG Preprocessing

EEG recordings (500 Hz sampling frequency, NetStation^7^ acquisition Software) were downsampled to 250 Hz and band-pass filtered between 1 and 40 Hz. The signal was visually inspected by an EEG expert (well trained in artifact recognition) and prominent artifacts removed by manual rejection. The noisy channels were excluded and interpolated *via* spline interpolation by visual inspection of the EEG expert. The electrode was classified as noisy if completely flat or showed a much higher magnitude than nearby electrodes (Cohen, 2014). Then, an average re-reference was applied on the retained signal (Ferree, 2006). Muscular, ocular, and electrocardiographic artifacts were removed using independent component analysis (ICA) in the EEGLAB toolbox (version 2019.1)^8^ of MATLAB (Delorme and Makeig, 2004). For each participant and condition (S1 and S2), the signal was divided in small epochs of 200 ms and evoked responses were removed from each epoch. This operation increased the stationarity of the signal without any event of interest linked to each epoch (Cohen, 2014). The epochs in the data segments between −5,000 ms (the onset of the rest phase) and −200 ms before the starting of the audio script, were used as baseline for trial normalization. Then, the fast Fourier transform analysis was performed with the Brainstorm software version 3.200818^9^ (Tadel et al., 2011).

#### EEG Asymmetry

First, for each participant, condition, and EEG epoch, we run a fast Fourier transform to obtain the power at 10 Hz, that is approximately the central frequency of the alpha spectrum domain (8–12 Hz—Klimesch, 2012). Then, for each participant, the alpha power was averaged between epochs in order to reduce the noise in the power spectrum and to consider only the frequency domain and not the time domain, since the two experimental conditions, S1 and S2, had different total durations. Then, for each participant, we baseline normalized the resulting power spectrum using the Decibel (dB) conversion formula (Cohen, 2014). This operation was performed in order to control and exclude possible trait differences in the EEG signals across participants and because “(…) raw power values are not normally distributed because they cannot be negative, and they are strongly positively skewed. This limits the ability to apply parametric statistical analyses (…)” (Cohen, 2014; p. 219). The left and right frontal (F3 and F4) and parietal (P3 and P4, as control) electrodes according to the 10–20 International System were used. These frontal sensors are EEG sites where the alpha asymmetry was classically reported (Allen et al., 2004). The parietal sensors in a specular position with respect to the frontal ones were considered control. In our study, we performed dB normalization instead of the classical log-transformation of EEG data performed by Allen et al. (2004). Thus, FAA and parietal (PAA) alpha asymmetries were calculated by subtracting the left from right normalized EEG activity [i.e., FAA = dB alpha (F4)—dB alpha (F3)]. Accordingly, we obtained the FAA index, where positive values reflect a higher alpha power in the right compared with the left hemisphere and consequently an approach motivation, while negative values correspond to a higher power in the left compared with the right hemisphere, thus reflecting an avoidance motivation toward the stimulus.

### Data Analysis

Preliminary, within the two scripts, we verified whether the compound score of the sentiment analysis reflected a negative (score ≤ −0.05), neutral (score between −0.05 and 0.05), or positive (score ≥ 0.05) valence (Hutto and Gilbert, 2015).

Then, the Bayesian statistical analyses were performed with the JASP software (Version 0.13.0)^10^. Firstly, unlike the frequentist approach, with the Bayesian statistics, starting from the prior probabilities on the two hypotheses (H0 and H1), we obtain the magnitude of the evidence in support of both H1 and H0 (Wagenmakers et al., 2017b). This allows us to estimate how confident we are in supporting, for instance, H0 (e.g., evidence in favor of a noncorrelation between the pupil diameter and the percentage of investment in S2) despite our small sample size. In fact, the Bayes Factor (BF_10_) that is the probability in support of H1 (values higher than 1) with respect to H0 (values below 1), could be interpreted as anecdotal, moderate, strong, very strong, and extreme, as evidenced in Table 1 (Wagenmakers et al., 2017a). In case the BF_10_ falls within the anecdotal evidence, we cannot fairly support our hypothesis (H1 or H0). Contrariwise, if the BF_10_ falls from the moderate to the extreme magnitude, we get progressively strong evidence in support of the hypothesis (H0 or H1). Secondly, the Bayesian statistic outperforms the frequentist approach in a small sample size scenario because offers the possibility to choose *a priori* how confident we are to find the evidence in support of H1 (Mcneish, 2016; Wagenmakers et al., 2017b; Zondervan-Zwijnenburg et al., 2017). The value of the prior could be set based on the current scientific knowledge about the topic of investigation (informative priors), or, if an extensive background lacks—such as the case of the relation between FAA and the willingness to pay—the use of a default uninformative prior is recommended. In the latter case, it is still possible to check the robustness of the evidence setting progressively wide and ultrawide prior values (robustness analysis), that is enhancing the expected probability of finding evidence in support of H1 with a high effect size (Van De Schoot et al., 2017; Zondervan-Zwijnenburg et al., 2017). Accordingly, the BF_10_ is considered fairly robust if the magnitude of the evidence is stable for most of the default, wide, and ultrawide prior values. Thus, the evidence taken from the results is the mostly supported one (e.g., if the evidence is anecdotal for the default but moderate for the wide and the ultrawide priors, the evidence in support of the hypothesis is considered mostly moderate).

**Table 1:** The interpretation of the B_10_, that is defined as the probability in support of the alternative hypothesis (H1, values higher than 1) with respect to null hypothesis (H0, values below 1 - Wagenmakers et al., 2017a).

**Interpretation**	**BF_**10**_**
Extreme evidence for H1	>100
Very strong evidence for H1	30–100
Strong evidence for H1	10–30
Moderate evidence for H1	3–10
Anecdotal evidence for H1	1–3
Equal evidence for H1 and H0	1
Anecdotal evidence for H0	1/3–1
Moderate evidence for H0	1/10–1/3
Strong evidence for H0	1/30–1/10
Very strong evidence for H0	1/100–1/30
Extreme evidence for H0	<1/100

As preliminary analysis, two one-tailed Bayesian paired-samples *T* tests were performed to control if the attitude score and percentage of investment, respectively, were higher in S2 than S1. Other three Bayesian paired samples *T* test were carried out to compare the score of the FAA, PAA, and pupil diameter between S1 and S2. As a further control, within each condition, we performed two Bayesian one-sample *T* test against zero to find possible unbalances in the FAA and PAA scores. Then, within each condition, we performed a Bayesian Kendall's Tau-b correlation to compare the attitude score and the percentage of investment with each other and with the FAA, the PAA, and the pupil diameter.

Each Bayesian *T* tests was redone three times setting a default (0.707), wide (1), and ultrawide (1.41) Cauchy prior, respectively (Wagenmakers et al., 2017a). Similarly, each Bayesian Kendall's Tau-B correlation was performed three times setting a default (1), wide (1.5), and ultrawide (2) stretched Beta prior (Wagenmakers et al., 2017a). The multiple comparison correction of the BF_10_ is recommended for pairwise comparisons when uninformative priors are used (De Jong, 2019; Han, 2019). Thus, we applied the false discovery rate Benjamini-Hochberg (FDR B-H) procedure to the Bayesian correlation test because for each condition and analysis, we performed seven pairwise comparisons. According to the Vovk-Sellke formula^11^, the BF_10_ of 2.46 (H1) and 0.406 (H0), that corresponded to a *p* value of 0.05 was used as threshold to consider a result as a false positive (Sellke et al., 2001; De Jong, 2019). Moreover, a false discovery rate of 0.25 was chosen to find the FDR B-H critical value. For each correlation analysis and separately for H1 and H0 significant results, firstly, the results were sorted in a descending (H1) or ascending (H0) order and then ranked according to their BF_10_. Then, according to the FDR B-H formula, we divided the rank of each false-positive result by the total number of tests (7) and multiplied the outcome by the false discovery rate (0.25). The resulting *p* value was then transformed in BF using again the Vovk-Sellke formula (Sellke et al., 2001; De Jong, 2019). This value represented the critical BF within which correctly accepting the hypotheses (H1 and H0). If the BF_10_ was below (H1) or above (H0), its critical value, we considered the result as a false positive. The FDR B-H correction was applied with the same procedure also when we redone the analysis with wide and ultrawide priors.

## Results

The descriptive of the variables involved in the analysis are presented in Table 2, while further details on the robustness analysis, as well as the prior and posterior distributions, are attached in the Supplementary Material 3.

**Table 2:** The sentiment analysis' compound score and the mean (±SD) of the percentage of investment, the attitude toward the offer, the EEG power asymmetry scores (computed in dB), and the pupil diameter in the little informative and standard script (S1) and in the highly informative and personalized script (S2).

**Variables**	**S1**	**S2**
Sentiment analysis' compound score	0.09	0.25
Investment (%)	14.762 (17.346)	32.143 (31.81)
Attitude toward the offer	5.095 (1.221)	6.095 (1.375)
EEG asymmetry F4-F3	−0.768 (1.366)	−0.095 (1.740)
EEG asymmetry P4-P3	0.650 (1.749)	0.173 (2.433)
Pupil diameter	0.283 (0.333)	0.391 (0.299)

*For the EEG asymmetry, we considered the right and left frontal (F4-F3) and parietal (P4-P3) electrodes*.

### Sentiment Analysis

The sentiment analysis revealed that both the commercial offers used a type of language which has a positive sentiment classification (compound score >0.05). The Script 1 assumed a compound score of 0.09, while the Script 2 of 0.25. Thus, we could exclude that the words presented in the two scripts had a connotation that could have evoked an *a priori* difference in participants' emotional valence.

### Bayesian Paired Samples *T*-Test

**Figure 1:**
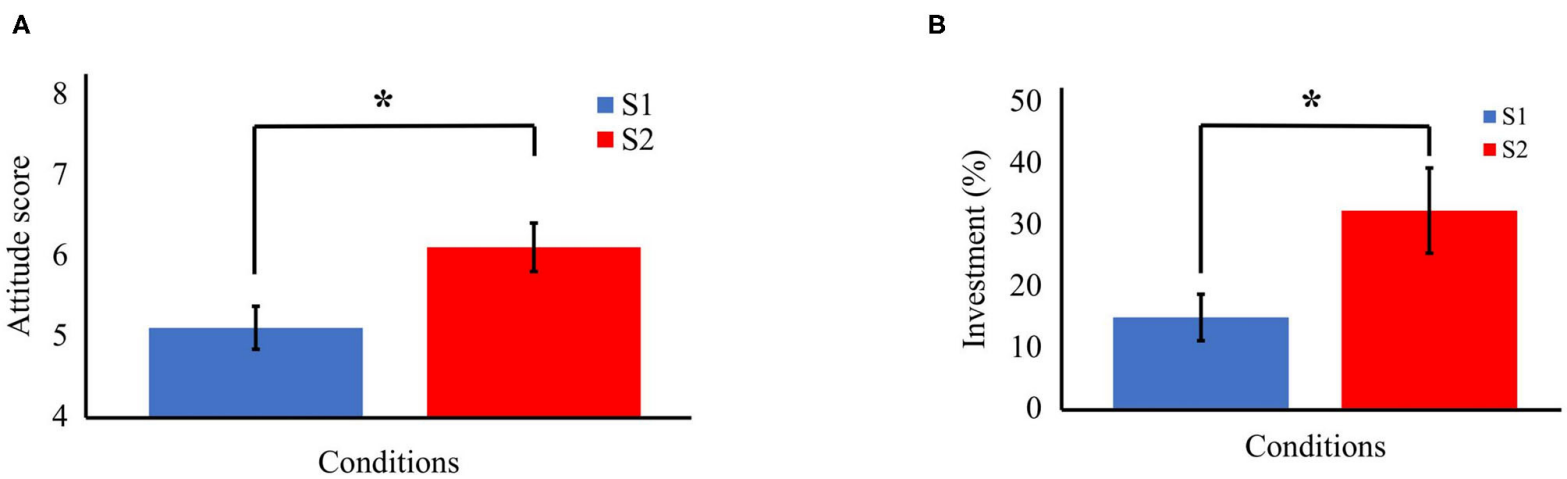
The significant results in support of H1 in the Bayesian paired-samples *T* test. **(A)** The attitude toward the offer score (vertical axis) in the two conditions (horizontal axis). In the highly informative and personalized script (S2), participants assumed a more-favorable and less-polarized attitude compared with the poorly informative and standard script (S1). The colored bars represent the mean in the two conditions, and the black lines the standard error with respect to the mean (*BF_10_ = 6). **(B)** The percentage of participants' investment (vertical axis) in the two conditions (horizontal axis). In the highly informative and personalized script (S2), participants invested a higher and more variable amount of money compared with the poorly informative and standard script (S1). The colored bars represent the mean in the two conditions, and the black lines the standard error with respect to the mean (*BF_10_ = 8. 8). *Moderate evidence in support of H1.

Two separate Bayesian paired samples *T*-test were performed to examine whether the attitude toward the offer score and the percentage of investment, respectively, where higher in S2 compared with S1. We found a moderate evidence in support of this hypothesis for both the attitude toward the offer (Figure 1A, BF_−0_ = 6) and the percentage of investment (Figure 1B; BF_−0_ = 8.8). The Bayes factor appears to be stable in both the analyses, ranging from BF_−0_ = 5.109 (wide) and BF_−0_ = 4.1 (ultrawide) for the attitude toward the offer, and from BF_−0_ = 7.7 (wide) and BF_−0_ = 6.2 (ultrawide) for the percentage of investment.

Moreover, we conducted other three separate Bayesian paired samples *T*-test (two-tailed) to compare pupil diameter, FAA, and PAA scores between conditions (S1 and S2). We found anecdotal evidence (BF_10_ = 0.5) in support of an equal FAA between S1 and S2. The BF_10_ was mostly anecdotal, because it was stable also for a wide prior (BF_10_ = 0.4) but moderate only for an ultrawide prior (BF_10_ = 0.3). Contrariwise, the evidence in support of an equality of the PAA scores between S1 and S2 is moderate (BF_10_ = 0.3). The BF_10_ remained stable also for wide (BF_10_ = 0.2) and ultrawide priors (BF_10_ = 0.2). For the pupil diameter, we found a BF_10_ = 0.5 that indicated an anecdotal evidence in support of H0, that is an equality between the pupil diameter scores between S1 and S2. The BF_10_ was mostly anecdotal and remained stable setting a wide prior (BF_10_ = 0.4) and became moderate only for an ultrawide prior (BF_10_ = 0.3).

### Bayesian One-Sample *T*-Test

**Figure 2:**
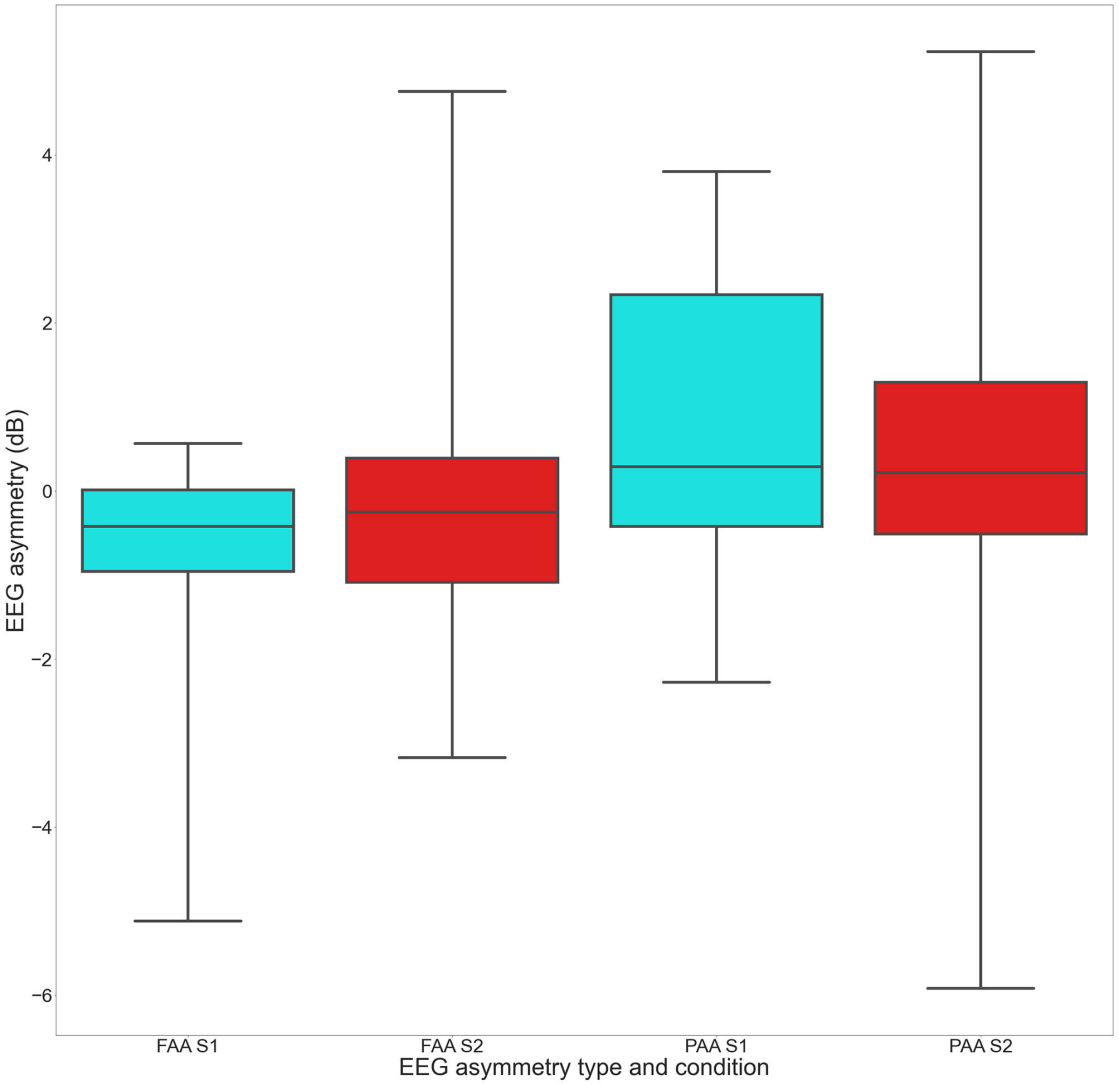
The EEG asymmetry score boxplots of the FAA and PAA in the two conditions (horizontal axis). The colored boxes represent the 25th to 75th percentiles of the scores, the line inside the box shows the median, and the whiskers represent the minimum and maximum scores. In S1 (cyan boxes), the FAA was polarized toward negative (avoidance) scores (B_−0_ = 6.), while in S2 (red boxes), we found moderate evidence in support of an equality of the FAA against zero (BF_10_ = 0.2). Similarly, in S2, we obtained a moderate evidence in support of an equality of the PAA score with respect to zero (B_10_ = 0.2).

As further control analyses, two separate Bayesian one-sample *T*-tests were performed within each condition (S1 and S2) to check whether the FAA and the PAA scores were unbalanced with respect to zero (Figure 2).

In S1, a moderate evidence in support of a polarization of the FAA toward negative (avoidance) scores (B_−0_ = 6.1) was found (one-tailed). The BF was robust also for wide (B_−0_ = 5.2) and ultrawide (B_−0_ = 4.2) priors. Another Bayesian one-sample *T*-test (two-tailed) was performed in S1 to compare the PAA score against zero. The analysis retuned a BF_10_ = 0.8 in favor of H0. Across wide and ultrawide priors, the BF_10_ appears to be stable, ranging, respectively, from BF_10_ = 0.6 to BF_10_ = 0.5. Thus, we obtained an anecdotal support in favor of an equality between the PAA score and zero in S1.

In S2, we found moderate evidence in support of an equality of the FAA against zero (BF_10_ = 0.2) that indicated a moderate evidence in support of H0 (two-tailed). This evidence is robust, because the BF_10_ across priors ranged from 0.2 (wide) to 0.1 (ultrawide). Similarly, we obtained a moderate evidence in support of an equality of the PAA score against zero with a B_10_ = 0.2 (two-tailed). The BF_10_ in support of H0 was stable, because it was moderate also for wide (BF_10_= 0.2) and ultrawide (BF_10_= 0.1) priors.

### Bayesian Kendall's Tau-B Correlation

Two separate nonparametric Bayesian Kendall's Tau-B correlation were performed within each condition (S1 and S2) to compare the attitude toward the offer and the percentage of investment between each other and with the FAA, the PAA, and the pupil diameter, respectively. The moderate, strong, very strong, and extreme BF reported were all accepted because they fall within the FDR B-H critical value.

**Figure 3:**
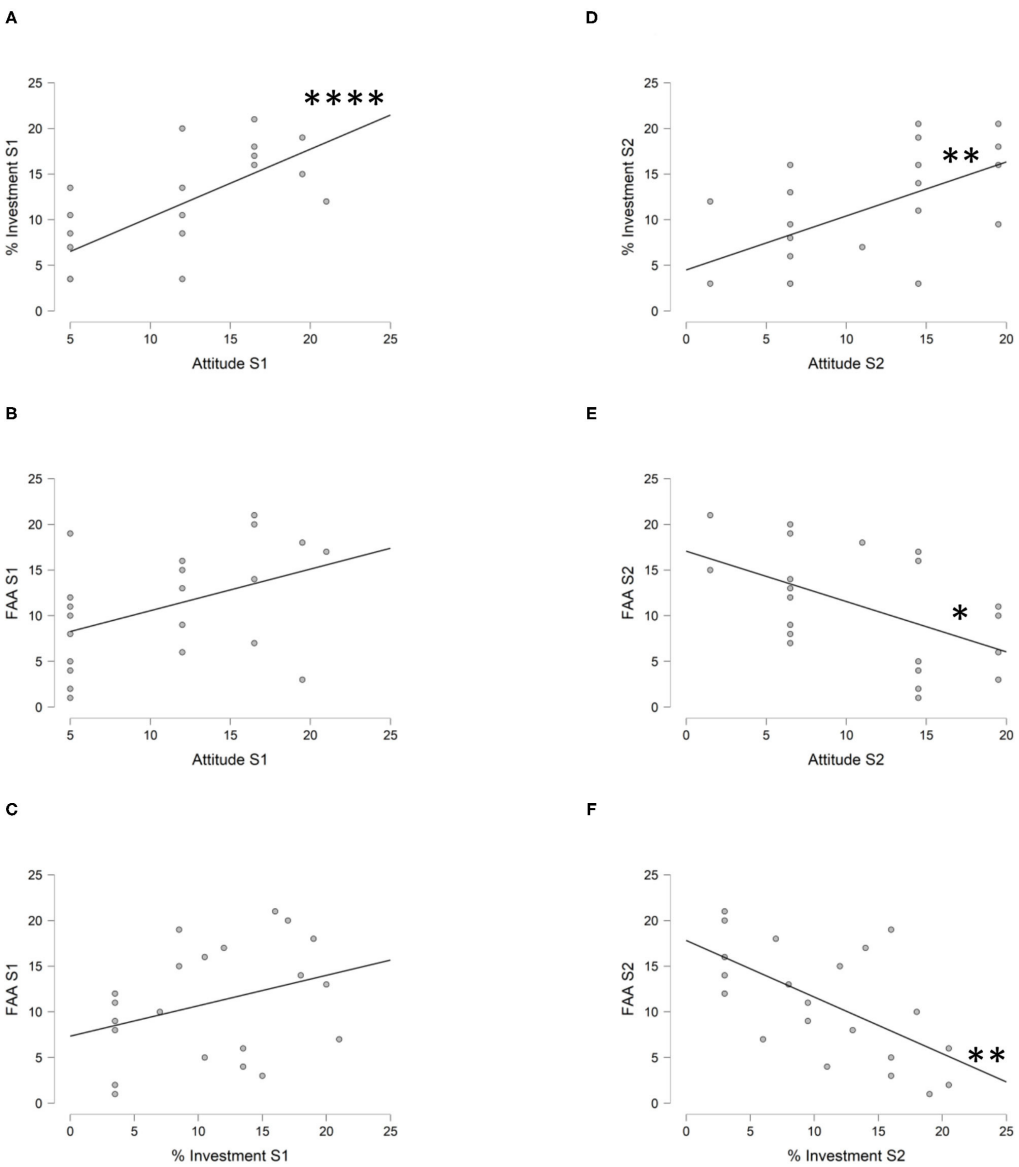
The Bayesian Kendall's Tau-B Correlation between attitude, investment and FAA within S1 and S2. **(A)** The scatter plot between the ranks of the attitude score (horizontal axis) and the percentage of investment (vertical axis) in the poorly informative and standard script (S1). In this condition, we found extreme evidence (BF_10_ = 102.3) that the more favorable is the attitude, the high is the percentage of investment, as well as the more unfavorable is the attitude, the low is the percentage of investment (****r_t_ = 0.559). **(B)** The scatter plot between the ranks of the attitude score (horizontal axis) and the FAA (vertical axis) in the poorly informative and standard script (S1) where we found anecdotal evidence (BF_10_ = 2.8) to sustain a positive correlation (*r*_t_ = 0.347). **(C)** The scatter plot between the ranks of the percentage of investment (horizontal axis) and the FAA (vertical axis) in the poorly informative and standard script (S1) where we found anecdotal evidence (BF_10_=0.7) in favor of a noncorrelation (*r*_t_ = 0.219). **(D)** The scatter plot between the ranks of the attitude score (horizontal axis) and the percentage of investment (vertical axis) in the highly informative and tailored script (S2). In this condition, we found a strong evidence (BF_10_ = 13.7) that the more favorable is the attitude, the high is the percentage of investment, as well as the more unfavorable is the attitude, the low is the percentage of investment (**r_t_ = 0.453). **(E)** The scatter plot between the ranks of the attitude score (horizontal axis) and the FAA (vertical axis) in the highly informative and tailored script (S2). In this condition, we found that the more favorable is the attitude, the more is participants' avoidance motivation toward the offer, as well as the more unfavorable is the attitude, the more is participants' approach motivation toward the offer (*r_t_ = −0.404; BF_10_ = 6.2). **(F)** The scatter plot between the ranks of the percentage of investment (horizontal axis) and the FAA (vertical axis) in the highly informative and tailored script (S2). In this condition, we found strong evidence (BF_10_ = 15.3) that the high is the investment, the more is participants' avoidance motivation toward the offer, as well as the low is the investment, the more is participants' approach motivation toward the offer (**r_t_ = −0.460). *Moderate evidence in support of H1; **strong evidence in support of H1; ****extreme evidence in support of H1.

In S1, we found an extreme evidence (BF_10_ = 102.3) in support of a positive correlation between the attitude toward the offer and the percentage of investment (Figure 3A—*r*_t_ = 0.559) that remains stable for wide (BF_10_ = 103) and became very strong for ultrawide (BF_10_ = 97.6) priors. Thus, the greater was the attitude of favor, the higher was the percentage of investment. When the attitude score was compared with the FAA score, we found an anecdotal evidence (BF_10_ = 2.8) in sustaining a positive correlation (Figure 3B—*r*_t_ = 0.347) that remains stable setting wide (BF_10_ = 2.4) and ultrawide (BF_10_ = 2.1) priors. Conversely, we found moderate evidence (BF_10_ = 0.3) in support of a noncorrelation between the attitude score and the PAA score (*r*_t_ = 0.006), confirmed also setting wide (BF_10_ = 0.2) and ultrawide (BF_10_ = 0.2) priors. Moreover, we found an equal evidence (BF_10_ = 1) in favor of a correlation and a noncorrelation between the attitude score and the pupil diameter (*r*_t_ = −0.259) that was mostly anecdotal in favor of a noncorrelation for wide (BF_10_ = 0.8) and ultrawide priors (BF_10_ = 0.7). When the percentage of investment was compared with the FAA score, we found an anecdotal evidence (BF_10_ = 0.7) in favor of a noncorrelation (Figure 3C—*r*_t_ = 0.219) that remained stable with wide (BF_10_ = 0.6) and ultrawide (BF_10_ = 0.5) priors. Moreover, we found an anecdotal evidence (BF_10_ = 0.4) in favor of a noncorrelation (*r*_t_ = 0.120) between the percentage of investment and the PAA, that was, however, mostly moderate because when setting wide (BF_10_ = 0.3) and ultrawide (BF_10_ = 0.2) priors became moderate. Similarly, we found an anecdotal evidence (BF_10_ = 0.3) in favor of a noncorrelation (*r*_t_ = −0.100) between the percentage of investment and the pupil diameter, that, however, became moderate setting both wide (BF_10_ = 0.3) and ultrawide priors (BF_10_ = 0.2).

In S2, we found a strong evidence (BF_10_ = 13.7) in support of a positive correlation (*r*_t_ = 0.453) between the attitude score and the percentage of investment (Figure 3D) that remained stable also for wide (BF_10_ = 12.5) and ultrawide (BF_10_ = 11.3) priors. Thus, the greater was the attitude of favor, the higher was the percentage of investment. When the attitude score was compared with the FAA score, we found a moderate evidence (BF_10_ = 6.2) in favor of a negative correlation (Figure 3E—*r*_t_ = −0.404) that remained stable also for wide (BF_10_ = 5.5) and ultrawide (BF_10_ = 4.9) priors. Thus, the greater was the favorable attitude, the higher was the avoidance motivation. Moreover, we found a moderate evidence (BF_10_ = 0.3) in support of a noncorrelation (*r*_t_ = −0.076) between the attitude score and the PAA, that remained stable also setting wide (BF_10_ = 0.2) and ultrawide priors (BF_10_ = 0.2). Similarly, we found a moderate evidence (BF_10_ = 0.3) in support of a noncorrelation (*r*_t_ = −0.044) between the attitude score and the pupil diameter, which remains stable also for wide (BF_10_ = 0.3) and ultrawide (BF_10_ = 0.2) priors. When the percentage of investment was correlated with the FAA score, we found a strong evidence (BF_10_ = 15.3) in favor of a negative correlation (Figure 3F—*r*_t_ = −0.460) that remains stable also for wide (BF_10_ = 14.2) and ultrawide (BF_10_ = 12.8) priors. Thus, the higher was the investment, the smaller was the FAA. Contrariwise, we found a moderate evidence (BF_10_ = 0.3) in support of a noncorrelation (*r*_t_ = 0.015) between the percentage of investment score and the PAA that remains stable also setting wide (BF_10_ = 0.2) and ultrawide priors (BF_10_ = 0.2). Similarly, we found a moderate evidence (BF_10_ = 0.3) to sustain a noncorrelation (*r*_t_ = 0.054) between the percentage of investment score and the pupil diameter that remained stable also for wide (BF_10_ = 0.2) and ultrawide (BF_10_ = 0.2) priors.

## Discussion

The main aim of this study was to evaluate whether the FAA score was correlated to customers' attitude and to their investment decision. With this aim, we manipulated a commercial offer in order to elicit diverse customers' attitudes and investment choices. Our results highlighted that the FAA, but not other peripheral body or neural control measures, has a robust association with participants' attitude and final decision, selectively when a highly informative and tailored commercial offer is presented. Consequently, this observation suggests that the reliability of the FAA as a marker of consumers' choices may be differentially affected by the specificity of different contextual factors, or diverse processes by which the decision is made, and could not be considered a general marker of individual decisions.

According to our experimental modulation, we verified that a poorly informative and “standard” commercial message elicited an unfavorable and less variable attitude as well as a limited and less variable percentage of investment in consumers compared with S2. Contrariwise, a highly informative and tailored message produced a more favorable and variable attitude and a larger and more interindividually variable percentage of investment compared with S1. This observation confirmed the success of our experimental manipulation. In fact, the mean and standard deviation of both the attitude and the percentage of investment were lower in S1 as compared with S2.

In S1, we found an extreme evidence of a positive correlation between the attitude score and the percentage of investment, likely due to the (negative) polarization that the scores assumed in this condition. In S1, the FAA is unbalanced toward negative (avoidance) scores (greater activity of the right frontal hemisphere compared with zero) and no significant correlation between the FAA and consumers' attitude or final investment scores was found. Conversely, in S2, we found a negative correlation between the attitude, the investment, and the FAA. This confirms that the FAA can be related to both consumers' attitude and final decision when a highly informative and tailored commercial offer is presented. This result is strengthened by the evidence that, unlike the FAA, the control variables (i.e., the PAA and the pupil diameter) did not correlate neither with the attitude nor with the percentage of investment. Therefore, the specific reliability of FAA as an indicator of consumers' decision appears to be influenced by the specificity of different contextual factors or diverse processes by which the decision is made.

From one side, we hypothesize our overt manipulation of the commercial offers could have biased individual mindsets so to influence both their attitude and final decision (Korteling et al., 2018). Especially for the poorly informative and “standard” commercial message (S1 condition), strong evidence demonstrates that a consumer commonly gives a little value to any advertisement, especially when the offer is uninformative and not personalized (Coulter et al., 2001; Goldsmith and Freiden, 2004; Xu, 2006; Ünal et al., 2011). In line with previous observations, our attitude score, that measured participants' perception of the offer, including their bias of perceived advantage versus disadvantage of the offer, was found lower in S1 compared with S2. Accordingly, we could hypothesize that in S1, participants may have interpreted the offer as unfair, thus polarizing both attitude and investment toward a low and little variable score, while in S2 the more informative and personalized offer may have invited consumers to use a less biased mindset, rather adopting rational stage of the decision-making process (Korteling et al., 2018).

Moreover, we found that a lower FFA corresponded to a favorable attitude toward the offer or a high investment, and, analogously, an unfavorable attitude and a low investment were related to a higher FFA. These observations may appear as counterintuitive as compared with the classical interpretation of approach and avoidance motivation of the FAA (Coan and Allen, 2003; Harmon-Jones et al., 2010; Ramsøy et al., 2018). Actually, alternative interpretation arises from studies that highlighted an association between the activity of the frontal right hemisphere and a regulatory system, namely the revised Behavioral Inhibition System (r-BIS; Gable et al., 2017), in both healthy subjects and brain-injured or psychiatric patients. This r-BIS would be responsible to supervise, govern, and regulate motivation and analyze the possible risks that arise when a decision has to be made. Thus, the r-BIS is a superordinate inhibitory control system that would intervene in case of conflicting motivation between approach and avoidance and “[…] *is thought to govern cognitive constructs of executive control and inhibitory function. This may result in suppression of a behavioral response or overriding motivational impulses* […]” and “[…] *is thought to alleviate tension between approach and avoidance systems by enhancing aversion of one behavior or the other*” (Gable et al., 2017, p. 3). A reduced recruitment of the r-BIS, corresponding to a low activation of the right frontal hemisphere and a consequently greater left activation, would be related to a more impulsive behavior, while an increased involvement of the r-BIS—associated with an increased activity of the right frontal cortex and a consequent less left hemispheric activation—would be associated with an active top-down control. In this view, we can consider that the results of the correlation that emerged between FAA and both attitude and investment choice reflected the activity of the r-BIS. Indeed, our experimental design in S2 could have forced participants to make a conflicting choice between an approach (i.e., positive attitude toward the offer) and avoidance (i.e., careful evaluation of individual past behaviors and needs) motivation and the r-BIS could have been used to regulate this conflicting decision. Furthermore, in line with our previous interpretation, the relationship with the attitude and investment would emerge only in S2, a condition in which the number of information and the personalization of the message invited consumers to assume a more rational mindset to get a reasoned decision. Specifically, in S2, a more positive attitude and a greater investment would correspond to a greater involvement of the r-BIS. We could therefore imagine that during the S2 condition, the r-BIS may have been involved in inhibiting the avoidance with respect to the approach motivation that may have supported consumers' more favorable attitude and higher investment compared with a lower response of the r-BIS. Thus, it is reasonable to speculate that in a situation in which participants decide to invest a large amount of money, there would be more need to inhibit the little value that consumers would commonly ascribe to advertisements (Coulter et al., 2001; Goldsmith and Freiden, 2004; Xu, 2006; Ünal et al., 2011) which may be reflected in a greater activation of the r-BIS.

To summarize, although it is not possible to sharply separate consumers' rational and intuitive mindsets, because both of them are involved and interact within the same decision-making process (Calabretta et al., 2016; Amidu et al., 2019), as well as we did not directly assess this aspect, the hypothesis that the FAA correlates with both attitude and decision, when consumers assume a more rational mindset to get a rational decision, could account for the results of both the S1 and S2 conditions. Three aspects support this hypothesis. Primarily, we evidenced how our attitude score, that measured participants' bias of perceived advantage and disadvantage of the offer, was found higher in S2 than in S1. Secondly, the direction of the correlation between the FAA and both attitude and investment, which emerged in S2, could reflect the activity of the r-BIS, a top-down control and monitoring system of the motivation of approach and avoidance located in the right frontal hemisphere (Gable et al., 2017). Thirdly, Hewig (2017), referring to the “Rubicon Model of Action Phases” framework (Heckhausen and Gollwitzer, 1987), argued that the FAA may reflect the volitional and intentional phase that occurs just prior to the action, as an intermediate step of a reasoned and planned decision-making process. Similarly, the consistency and the rationality of the decision-making process improve when we are exposed to highly informative messages (Hahn et al., 1992; Korteling et al., 2018). Accordingly, it might be possible that a link between the FAA and the final decision may not emerge when the characteristics of the message lead consumers to use a less rational, but more spontaneous, decisional process, as may have happened in S1. Although partly accounting for the conflicting evidence present in literature on the relation between FAA and consumers' decision—neither Ramsøy et al. (2018), nor Ravaja et al. (2013) manipulated consumers' attitude and final investment—this suggestion must be confirmed by further experiments, but could represent a starting point for future studies on this topic.

The present study has limitations that future investigations should consider and improve. Firstly, we did not have preferences and investment decision scores of the commercial offers in a larger and independent sample of customers for a large-scale generalization of the link between the FAA and their decision (Hakim and Levy, 2018). However, in our study, we used a representative sample of the population of possible insurance policy customers. For these reasons, although further efforts will be required in future studies to increase the experimental sample, we are confident that our results can be generalized at the population level. Secondly, although the sample size is consistent with other similar EEG studies in marketing research (e.g., Vecchiato et al., 2010; Daugherty et al., 2016; Gordon et al., 2018; Ramsøy et al., 2018), the participant number could be increased, in order to carry out parametric statistical tests, such as linear regression, which could determine whether the FAA score predicts consumers' attitude and decision. However, to overcome the small sample limitation, we used Bayesian statistic that outperforms the frequentist approach in a small-sample-size scenario (Mcneish, 2016; Wagenmakers et al., 2017b; Zondervan-Zwijnenburg et al., 2017) and allows future researchers to use our results to set more informative priors compared with our case when investigating this topic. Thirdly, we did not integrate the EEG and the eye tracker with other psychophysiological measures and techniques. The specific objective of the present study was to investigate the relation between the FAA with consumers' attitude and decision. For this reason, we used only the specific measures required for our goal. However, future research based on our results may broaden the range of measures and objectives also by using different body peripheral and neural parameters, in order to investigate other possible psychophysiological measures related to consumers' decisions (Cartocci et al., 2017; Hakim and Levy, 2018; Ocklenburg et al., 2018). Fourth, although we have preliminarily checked our scripts for their positive, negative, or neutral valence, future research could make a further step by controlling more specific categories of words that could influence a priori participants' attitudes and therefore their brain activity (O'Donnell et al., 2015). Fifth, although we only used the frequency domain in the EEG analysis, we designed two commercial script (S1 and S2) with different lengths, and we cannot account the extent of how this might have affected the results. Finally, it would be interesting to test an additional experimental condition that polarized consumers' attitudes and investments toward high and favorable scores, to understand whether, even in this case, the relation between FAA, attitude, and decision is similar to our S1 results. Beside these explanations, some methodological procedures that we peculiarly adopted with respect to other research could also account for our evidence. Firstly, we increased the external validity of the experimental design by presenting a gambling game that motivated and let participants gain the virtual real-like wallet that was then used for the investment. Secondly, we baseline-normalized the EEG signal acquired during the presentation of the commercial scripts by using the Decibel conversion formula. As a matter of fact, some studies highlighted that possible interindividual trait predispositions of FAA, measured during a baseline EEG registration, may shape the variability of the FAA collected during an experimental task (i.e., Uusberg et al., 2014).

In conclusion, in our research, we underlined how the FAA has a robust association with consumers' attitude and final decision, though this relation emerges specifically when the commercial script provides an adequate number of information and is customized, likely leading the consumer to a more reasoned and planned decision-making process. This correlation is specific of the FAA because both peripheral body and neural control measures (such as the PAA and the pupil diameter) were not associated with both attitude and final investment. Moreover, we also underlined how in a tailored and more informative commercial script the FAA may reflect the involvement of the r-BIS. This control system could intervene to monitor and govern possible conflicts between an approach and avoidance motivation. This evidence provide further indications on the possible association between the FAA and both attitude and final decision, although further researches are needed. Finding and validating psychophysiological measures, such as the FAA, might have a fundamental impact on Consumer Neuroscience, but the reliability and modifiability of this index has still to be validated across different (experimental) conditions. With the advent of low-cost EEG headsets, this index could even be easily used in ecological settings, both in applied research and in business consultancy, and could be useful evidence-based measure beside self-reports of customers' attitude and choices.

## Data Availability Statement

The raw data supporting the conclusions of this article will be made available by the authors, without undue reservation.

## Ethics Statement

The studies involving human participants were reviewed and approved by Area Vasta Nord Ovest Ethics Committee (protocol n. 24579/2018). The patients/participants provided their written informed consent to participate in this study.

## Author Contributions

FDG, MO, AM, NL, and ER contributed to the design and the conception of the research. FDG and MO contributed to the implementation and the analysis of the results. FDG contributed to writing the manuscript. MO, AM, NL, and ER contributed to the manuscript revision. SD'A, as member of Intesa Sanpaolo Innovation Center S.p.A., has contributed to research funding. MO, AM, SD'A, NL, and ER read and approved the submitted version of the manuscript. All authors contributed to the article and approved the submitted version.

## Conflict of Interest

The authors declare that the research was conducted in the absence of any commercial or financial relationships that could be construed as a potential conflict of interest.

## Footnotes

^1^E-Prime (Version 2.0) [Computer software].

^2^Python (Version 3.8) [Computer software]. (2020).

^3^VederSentiment (Version 3.3.2) [Computer software]. (2020).

^4^SpaCy (Version 2.3.2) [Computer software]. (2020).

^5^CHAP. (2020). *Open Source Software for Processing and Analyzing Pupillometry Data [Computer software]*. Retrieved from: https://in.bgu.ac.il/en/Labs/CNL/chap/default.aspx

^6^MATLAB (Version R2018b) [Computer software]. (2018).

^7^NetStation (Version 5) [Computer software].

^8^EEGLAB (Version 2019.1) [Computer software]. (2019).

^9^Brainstorm (Version 3.200818) [Computer software]. (2020).

^10^JASP (Version 0.13.0) [Computer software]. (2020).

^11^To compute the BF from the *p* value the formula is: BF = 1/(-e *p* ln(*p*)).
