# Supplementary material for: The Relation Between Consumers' Frontal Alpha Asymmetry, Attitude, and Investment Decision
Source: Front Neurosci. 2021 Jan 21;14:577978. doi: 10.3389/fnins.2020.577978 (PMC7874093; doi:10.3389/fnins.2020.577978)
Supplement: Supplementary file 1 [file Data_Sheet_1.docx]

Supplementary Material 1

# Script 1

## Italian

Buongiorno, sono Simona Rossi di [nome della banca/compagnia]. La disturbo? Posso rubarle pochi minuti? La chiamo perché abbiamo selezionato 50 clienti tra cui lei appunto, per proporre delle nuove polizze assicurative a condizioni particolarmente vantaggiose. Delle polizze con caratteristiche nuove e che rimarranno in promozione solo per questo mese. Ce ne sono di vari tipi per le diverse esigenze. Sicuramente potrebbe trovarne una che risponde ai suoi bisogni e ai suoi interessi, a condizioni come le dicevo particolarmente vantaggiose ma solo in questo mese e solo per voi 50 clienti selezionati. Se vuole possiamo fissare un appuntamento in filiale per esempio la prossima settimana, così gliele posso spiegare più nel dettaglio e possiamo vedere insieme le sue esigenze.

## English

Hello, I am Simona Rossi from [name of the bank / company]. Am I disturbing you? Can I borrow you for a few minutes? I am calling because we have selected 50 clients, including you, to propose new insurance policies at particularly advantageous conditions. Policies with new features and that will remain on promotion only during this month. There are various types of them for different needs. Surely you could find one that meets your needs and interests, under conditions as I said particularly advantageous but only this month and only for 50 selected customers like you. If you want we can schedule an appointment at the branch for example next week, so I can explain them in more detail and we can have a look together to your needs.

# Script 2

## Italian

Buongiorno, sono Alessandra Bianchi di [nome della banca/compagnia]. La chiamo per informarla che in questi giorni stiamo presentando ai nostri clienti delle nuove polizze assicurative che potrebbero interessarle. Poiché ne esistono di vari tipi mi sono permessa di esaminare il questionario da lei compilato per cominciare a prendere in considerazione le sue esigenze, così da scegliere il prodotto per lei più interessante, senza farle perdere troppo tempo. Se posso le accennerei brevemente quello che a mio parere più si adatta al suo profilo. Poi se le interessa possiamo fissare un appuntamento in filiale lo vediamo nel dettaglio. Ad esempio, stavo pensando alla polizza “Per Me Protezione” che data la sua età, la sua attività, mi sembra la più conveniente. Infatti, è una polizza che può comprendere più moduli che lei può combinare come meglio crede, è molto flessibile perché la composizione di moduli può essere modificata via via a seconda di eventuali cambiamenti di vita che ovviamente si possono verificare nella vita di ognuno di noi, no? Che so, la nascita di un figlio, il cambio di casa, il cambio di lavoro, o anche in considerazione della situazione economica generale che come saprà in questo momento è in continua evoluzione. Inoltre, è molto conveniente perché più moduli acquista e più lo sconto aumenta. Quindi, se le interessa, possiamo fissare un appuntamento in filiale anche subito nei prossimi giorni.

## English

Hello, I am Alessandra Bianchi from [name of the bank / company]. I am calling to inform you that we are currently presenting our clients with new insurance policies that may be of interest to you. Since there are various types of these, I have allowed myself to examine the questionnaire you have filled out in order to begin to consider your needs, so as to choose the most interesting product for you, without wasting too much time. If I may, I would briefly mention what I think most fits your profile. Then if you are interested, we can schedule an appointment at the branch. For example, I was thinking about the insurance policy “For Me Protection” which given your age, your activity, seems to me the most convenient. In fact, it is a policy that can include multiple modules that you can combine as you wish, it is very flexible because the composition of modules can be modified gradually depending on any changes in life that obviously can occur in our daily life, no? I don’t know, the birth of a child, the change of home, the change of job, or even considering the general economic situation that as you know at this time is constantly evolving. In addition, it is very convenient because the more modules you buy the more the discount increases. So, if you are interested, we can schedule an appointment at the branch right away in the next few days.
