# Supplementary material for: The Relation Between Consumers' Frontal Alpha Asymmetry, Attitude, and Investment Decision
Source: Front Neurosci. 2021 Jan 21;14:577978. doi: 10.3389/fnins.2020.577978 (PMC7874093; doi:10.3389/fnins.2020.577978)
Supplement: Supplementary file 2 [file Data_Sheet_2.docx]

Supplementary Material 2

# Source code for the sentiment analysis

#Install spacy and vedersentiment toolbox

pip install spacy vaderSentiment

#Import packages and prepare the analysis

import spacy

import numpy as np

import pandas as pd

from spacy.lang.en import English

from vaderSentiment import vaderSentiment

en = English()

en.add_pipe(en.create_pipe('sentencizer'))

#Sentiment analysis in S1

result_S1 = en("Hello, I am Simona Rossi from [name of the bank / company]. \

Am I disturbing you? Can I borrow you for a few minutes? I am \

calling because we have selected 50 clients, including you, to \

propose new insurance policies at particularly advantageous \

conditions. Policies with new features and that will remain on \

promotion only during this month. There are various types of them \

for different needs. Surely you could find one that meets your needs \

and interests, under conditions as I said particularly advantageous \

but only this month and only for 50 selected customers like you. If \

you want we can schedule an appointment at the branch for example \

next week, so I can explain them in more detail and we can have a \

look together to your needs.")

sentences_S1 = [str(s) for s in result_S1.sents]

analyzer_S1 = vaderSentiment.SentimentIntensityAnalyzer()

sentiment_S1 = [analyzer_S1.polarity_scores(str(s)) for s in sentences_S1]

sent_S1_df=pd.DataFrame(sentiment_S1)

compound_S1=np.mean(sent_S1_df['compound'])

#Sentiment analysis in S2

result_S2 = en("Hello, I am Alessandra Bianchi from [name of the bank / company]. \

I am calling to inform you that we are currently presenting our \

clients with new insurance policies that may be of interest to you. \

Since there are various types of these, I have allowed myself to \

examine the questionnaire you have filled out in order to begin \

to consider your needs, so as to choose the most interesting product \

for you, without wasting too much time. If I may, I would briefly \

mention what I think most fits your profile. Then if you are interested, \

we can schedule an appointment at the branch. For example, I was \

thinking about the insurance policy “For Me Protection” which given \

your age, your activity, seems to me the most convenient. In fact, \

it is a policy that can include multiple modules that you can combine \

as you wish, it is very flexible because the composition of modules can \

be modified gradually depending on any changes in life that obviously can \

occur in our daily life, no? I don’t know, the birth of a child, the change \

of home, the change of job, or even considering the general economic situation \

that as you know at this time is constantly evolving. In addition, it is very \

convenient because the more modules you buy the more the discount increases. \

So, if you are interested, we can schedule an appointment at the branch right \

away in the next few days.")

sentences_S2 = [str(s) for s in result_S2.sents]

analyzer_S2 = vaderSentiment.SentimentIntensityAnalyzer()

sentiment_S2 = [analyzer_S2.polarity_scores(str(s)) for s in sentences_S2]

sent_S2_df=pd.DataFrame(sentiment_S2)

compound_S2=np.mean(sent_S2_df['compound'])

#Display the results of the compound score in S1 and S2

summary=[{'Compound':compound_S1},{'Compound':compound_S2}]

sum_df=pd.DataFrame(summary)

sum_df.index=['S1','S2']

print(sum_df)
