# Supplementary material for: The Relation Between Consumers' Frontal Alpha Asymmetry, Attitude, and Investment Decision
Source: Front Neurosci. 2021 Jan 21;14:577978. doi: 10.3389/fnins.2020.577978 (PMC7874093; doi:10.3389/fnins.2020.577978)
Supplement: Supplementary file 3 [file Data_Sheet_3.docx]

Supplementary Material 3

# Robustness analysis of the Bayesian Paired-Sampled T-Test

## Attitude S1 – Attitude S2


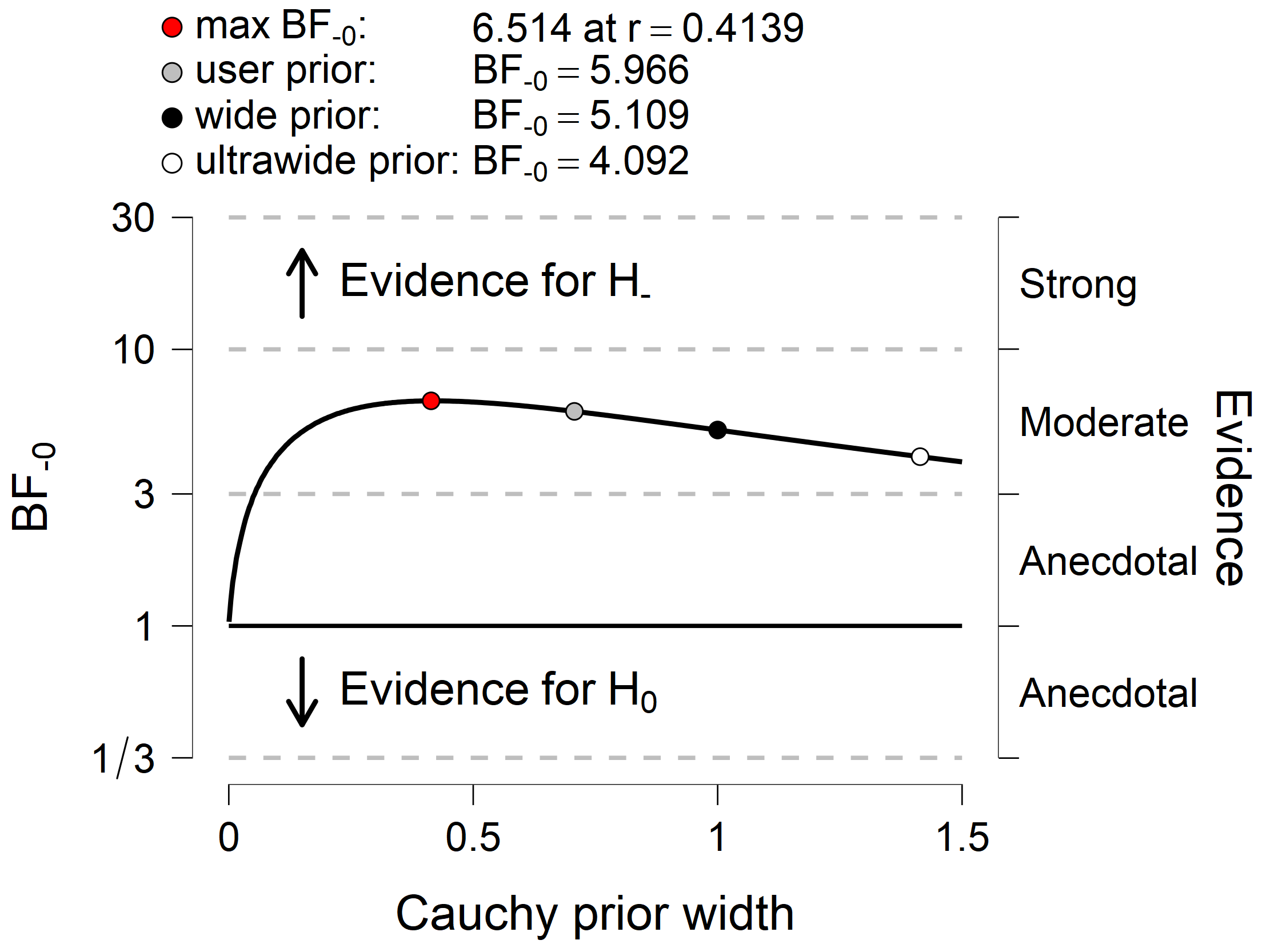


**Supplementary Figure 1.** Robustness analysis for the BF_-0_ when comparing of the attitude score between S1 and S2 (one-tailed).


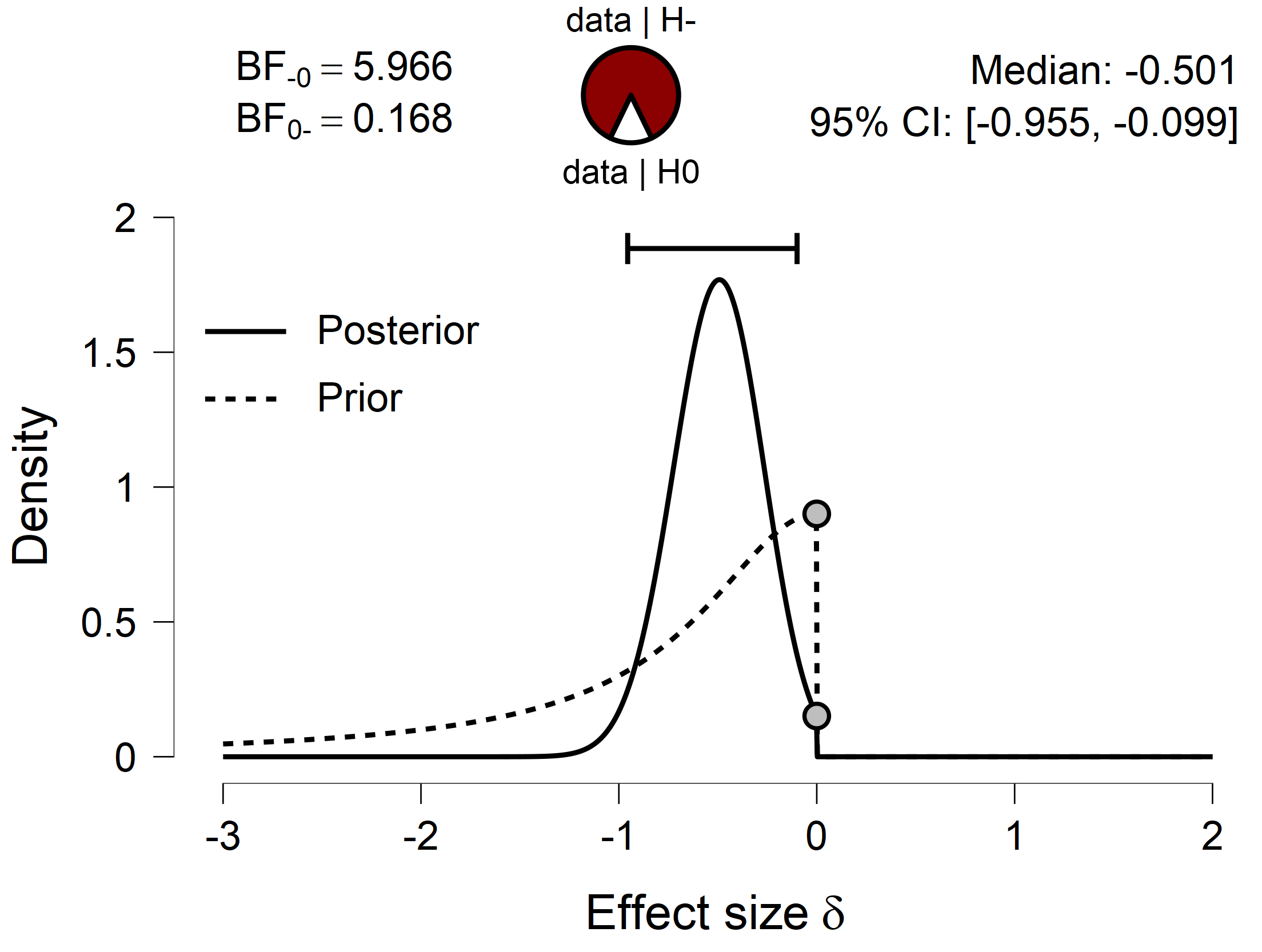


**Supplementary Figure 2.** Prior and posterior distribution of the effect size under H1 setting a default prior.

## Percentage of investment S1 – Percentage of investment S2


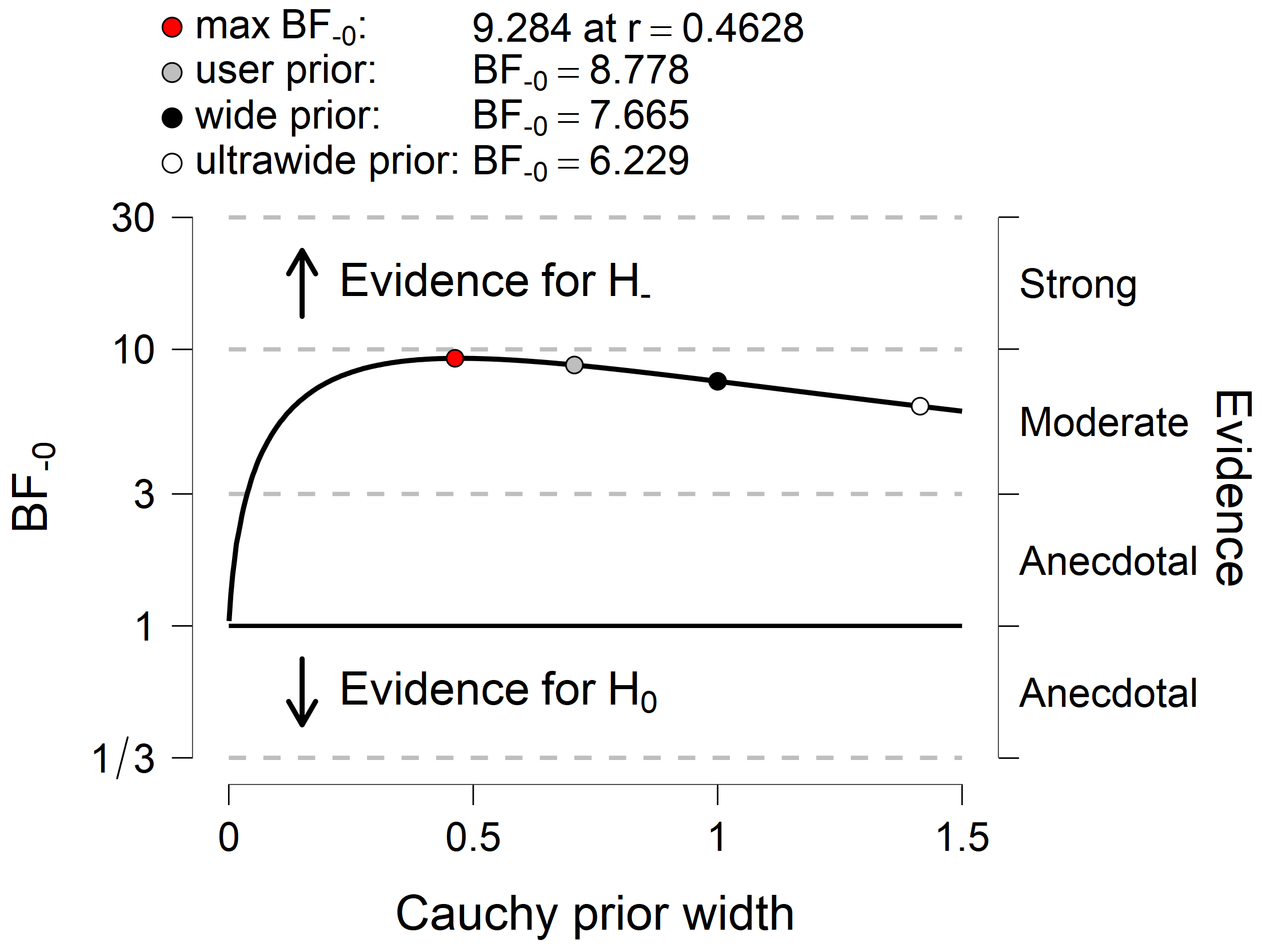


**Supplementary Figure 3.** Robustness analysis for the BF_-0_ when comparing of the percentage of investment between S1 and S2 (one-tailed).


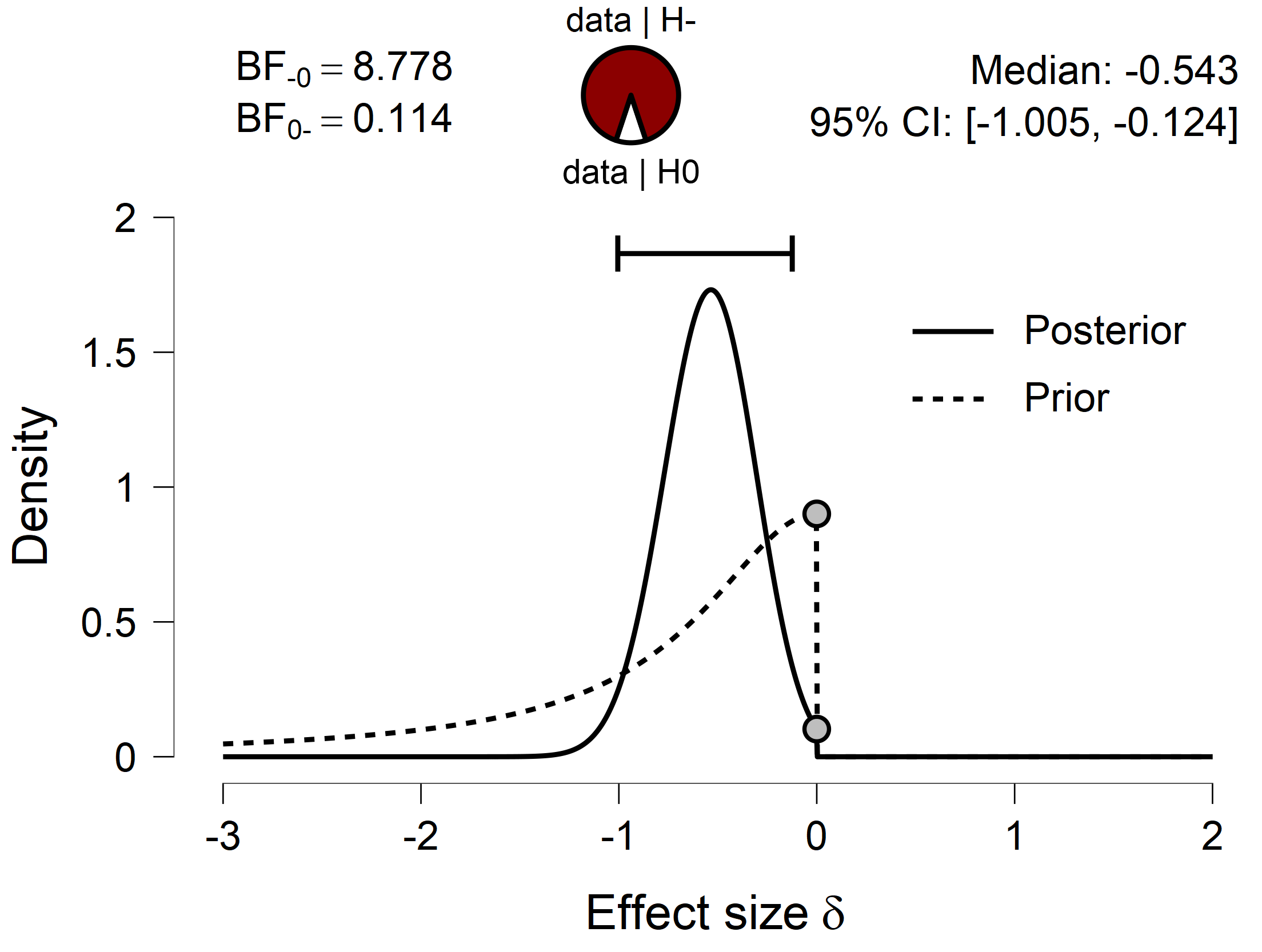


**Supplementary Figure 4.** Prior and posterior distribution of the effect size under H1 setting a default prior.

## FAA S1 – FAA S2


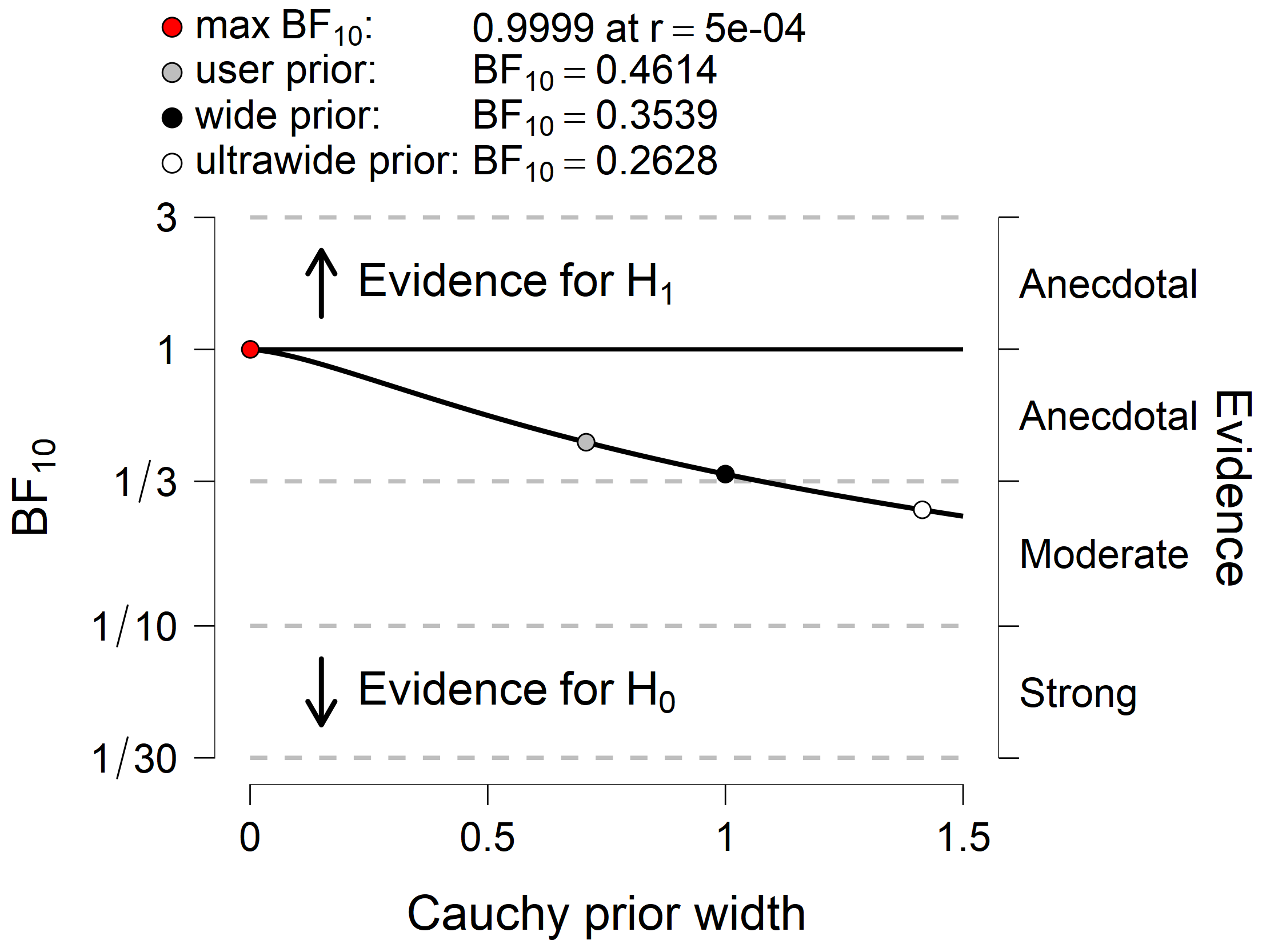


**Supplementary Figure 5.** Robustness analysis for the BF_10_ when comparing of the Frontal Alpha Asymmetry (FAA) between S1 and S2.


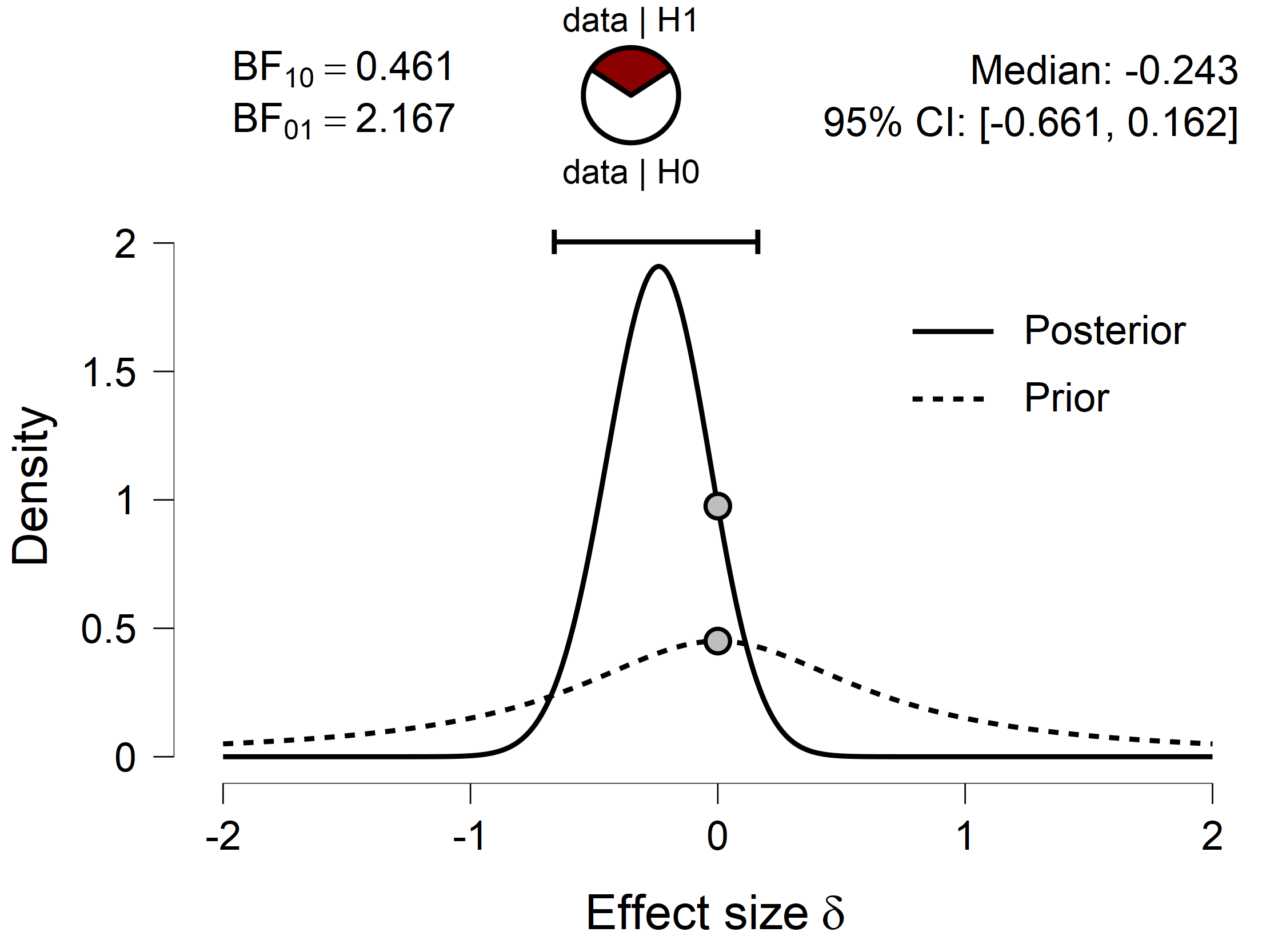


**Supplementary Figure 6.** Prior and posterior distribution of the effect size under H1 setting a default prior.

## PAA S1 – PAA S2


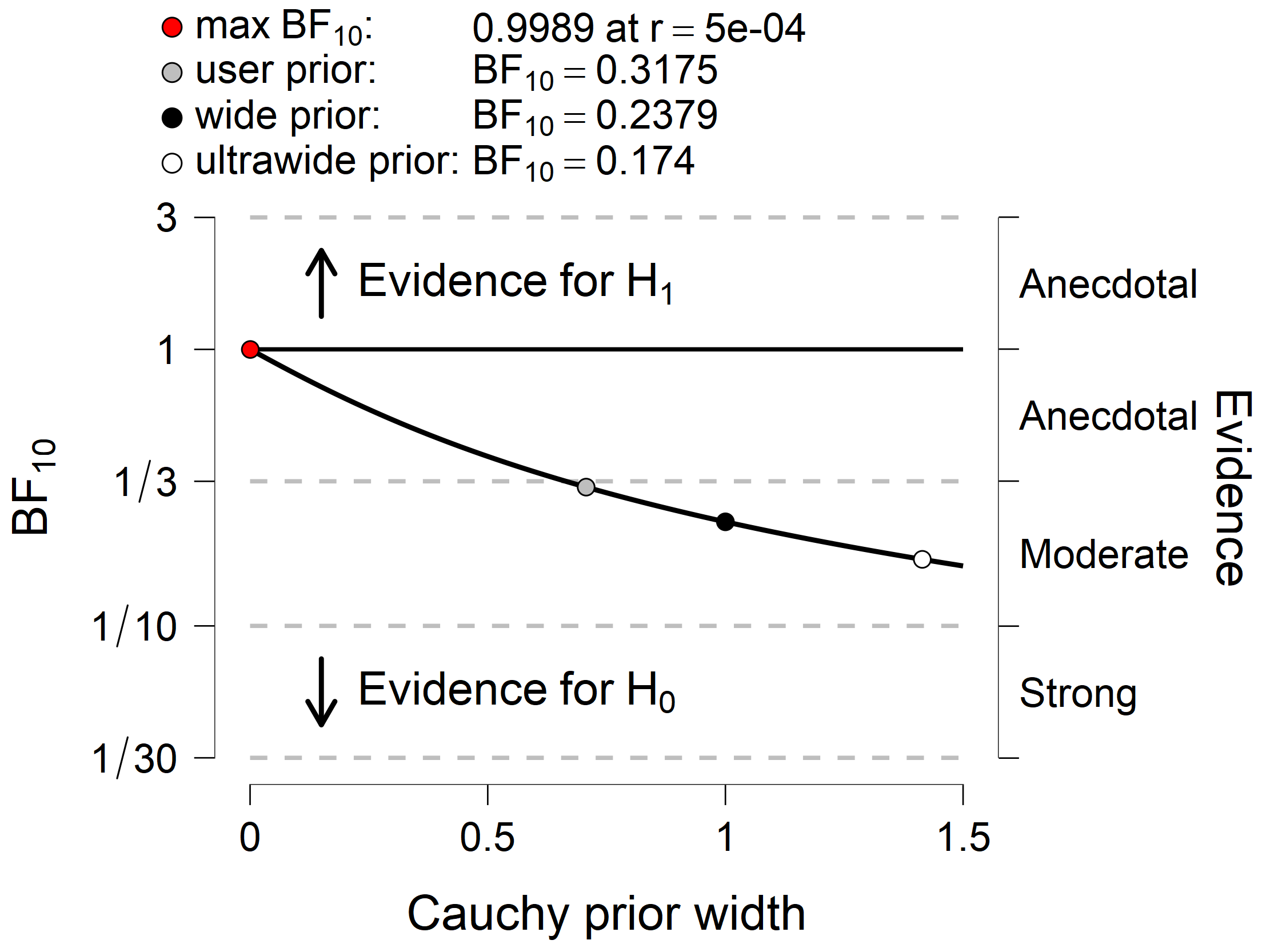


**Supplementary Figure 7.** Robustness analysis for the BF_10_ when comparing of the Parietal Alpha Asymmetry (PAA) between S1 and S2.


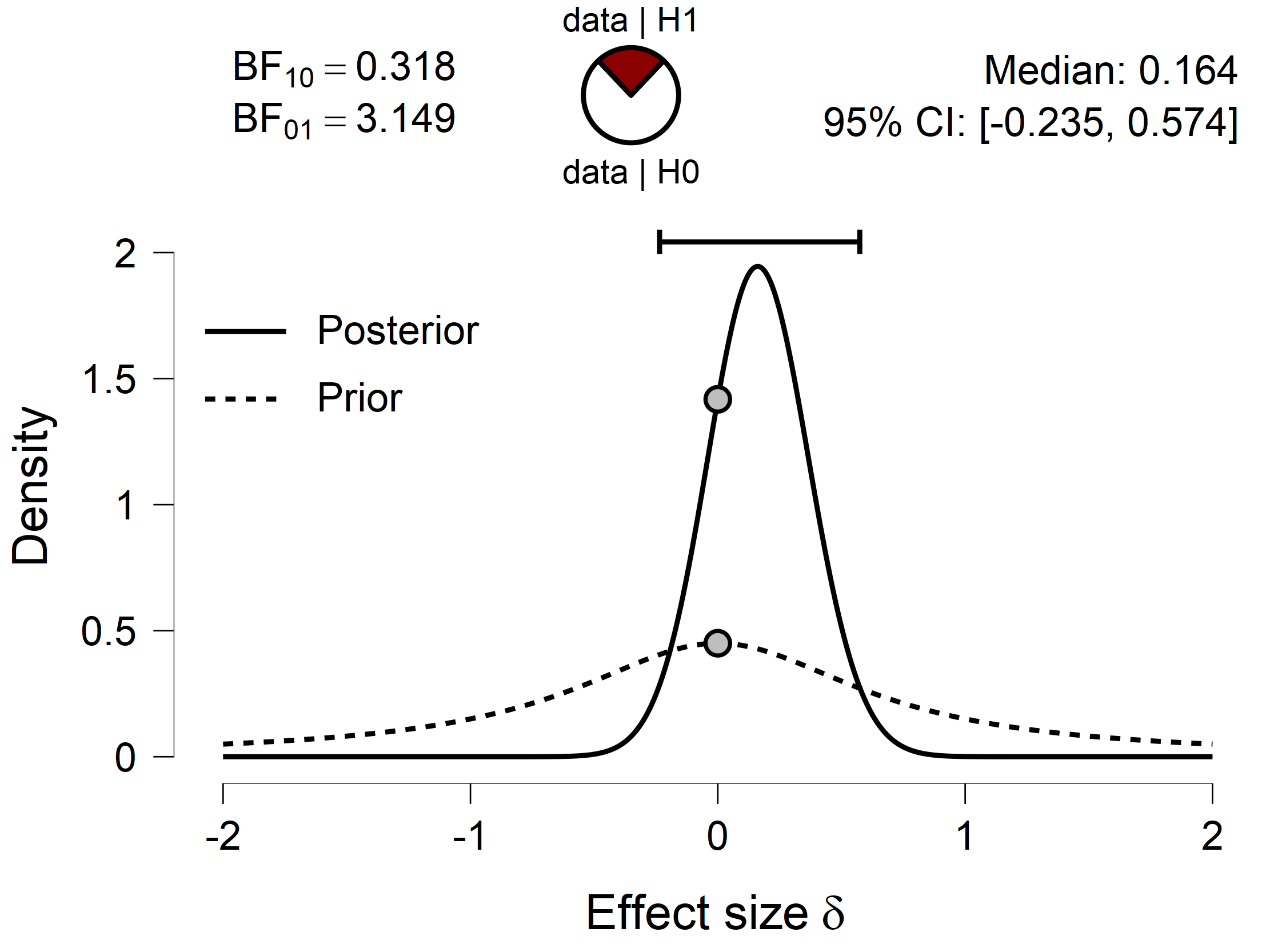


**Supplementary Figure 8.** Prior and posterior distribution of the effect size under H1 setting a default prior.

## Pupil Diameter S1 – Pupil Diameter S2


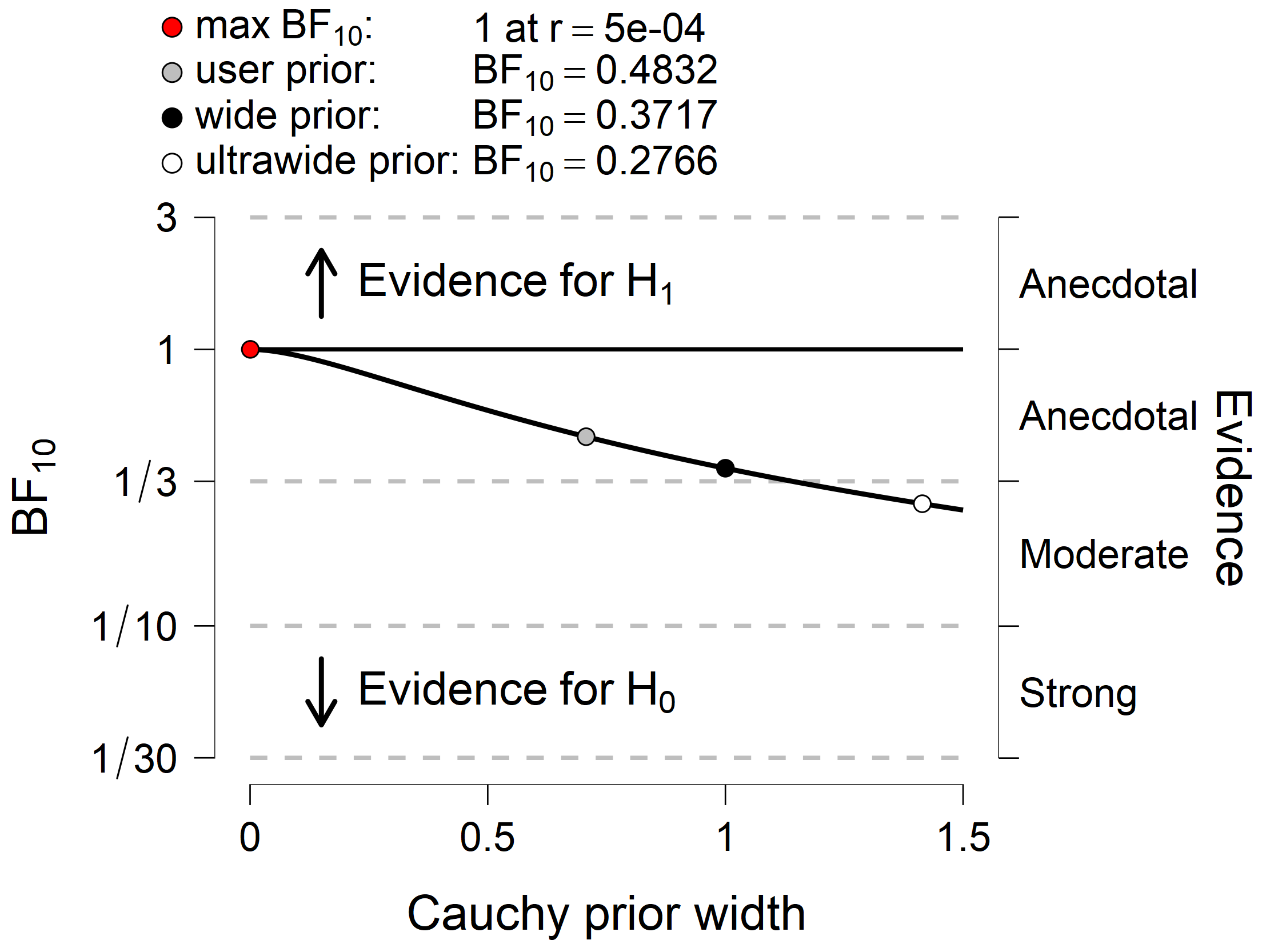


**Supplementary Figure 9.** Robustness analysis for the BF_10_ when comparing of the Pupil Diameter between S1 and S2.


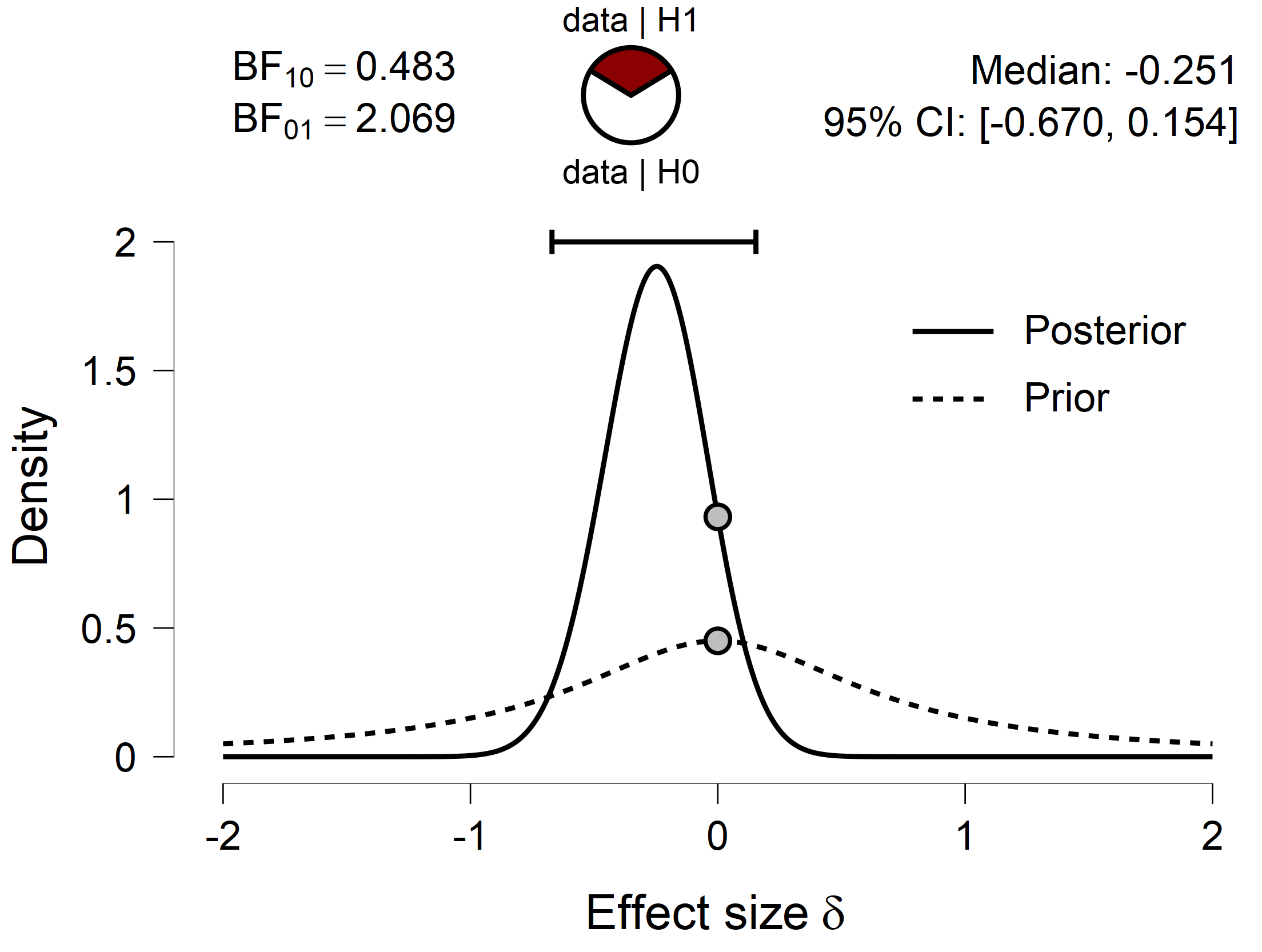


**Supplementary Figure 10.** Prior and posterior distribution of the effect size under H1 setting a default prior.

# Robustness analysis of the Bayesian One-Sample T-Test

## FAA S1


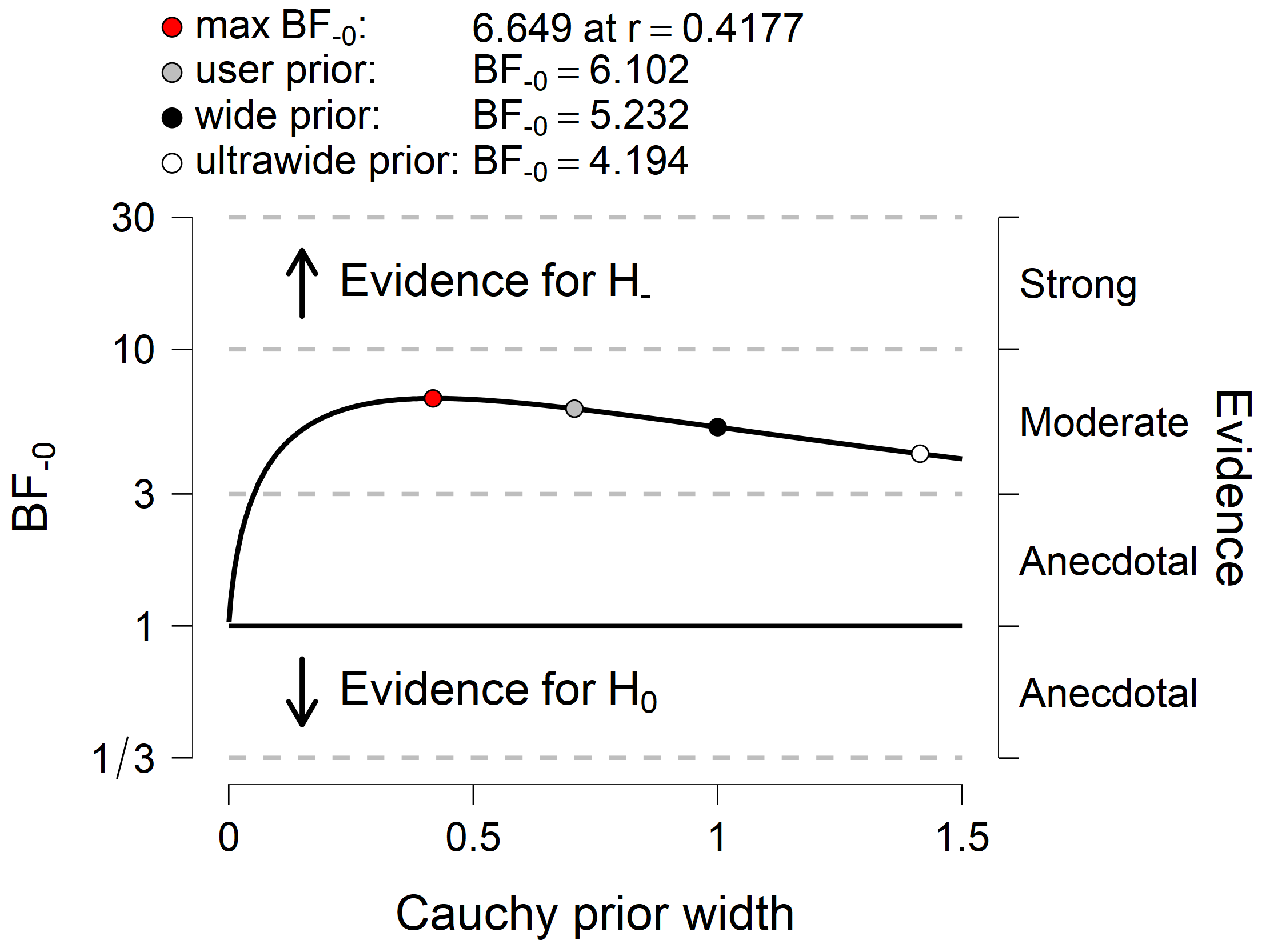


**Supplementary Figure 11.** Robustness analysis for the BF_-0_ when comparing of the FAA in S1 against zero (one-tailed).


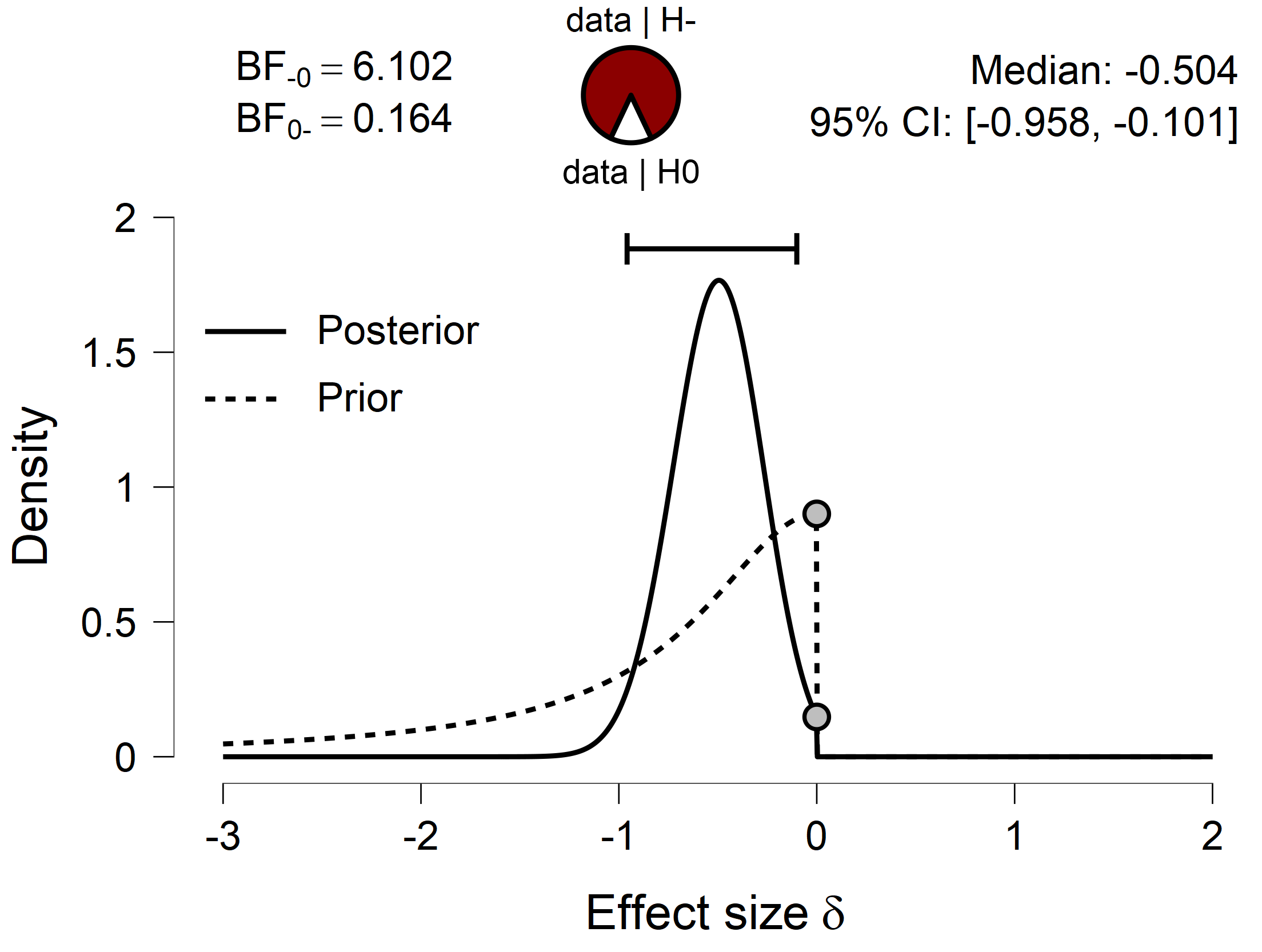


**Supplementary Figure 12.** Prior and posterior distribution of the effect size under H1 setting a default prior.

## FAA S2


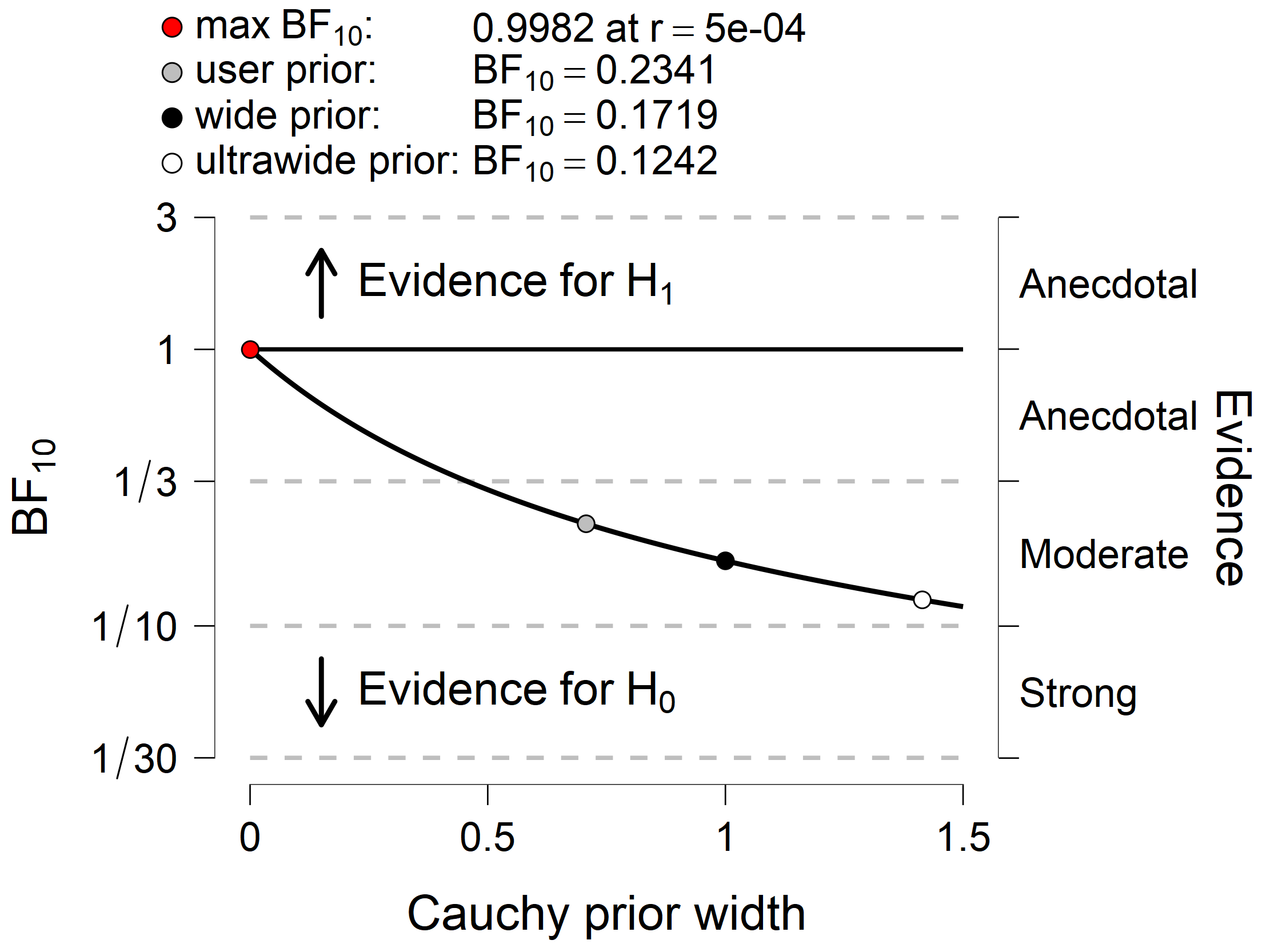


**Supplementary Figure 13.** Robustness analysis for the BF_10_ when comparing of the FAA in S2 against zero.


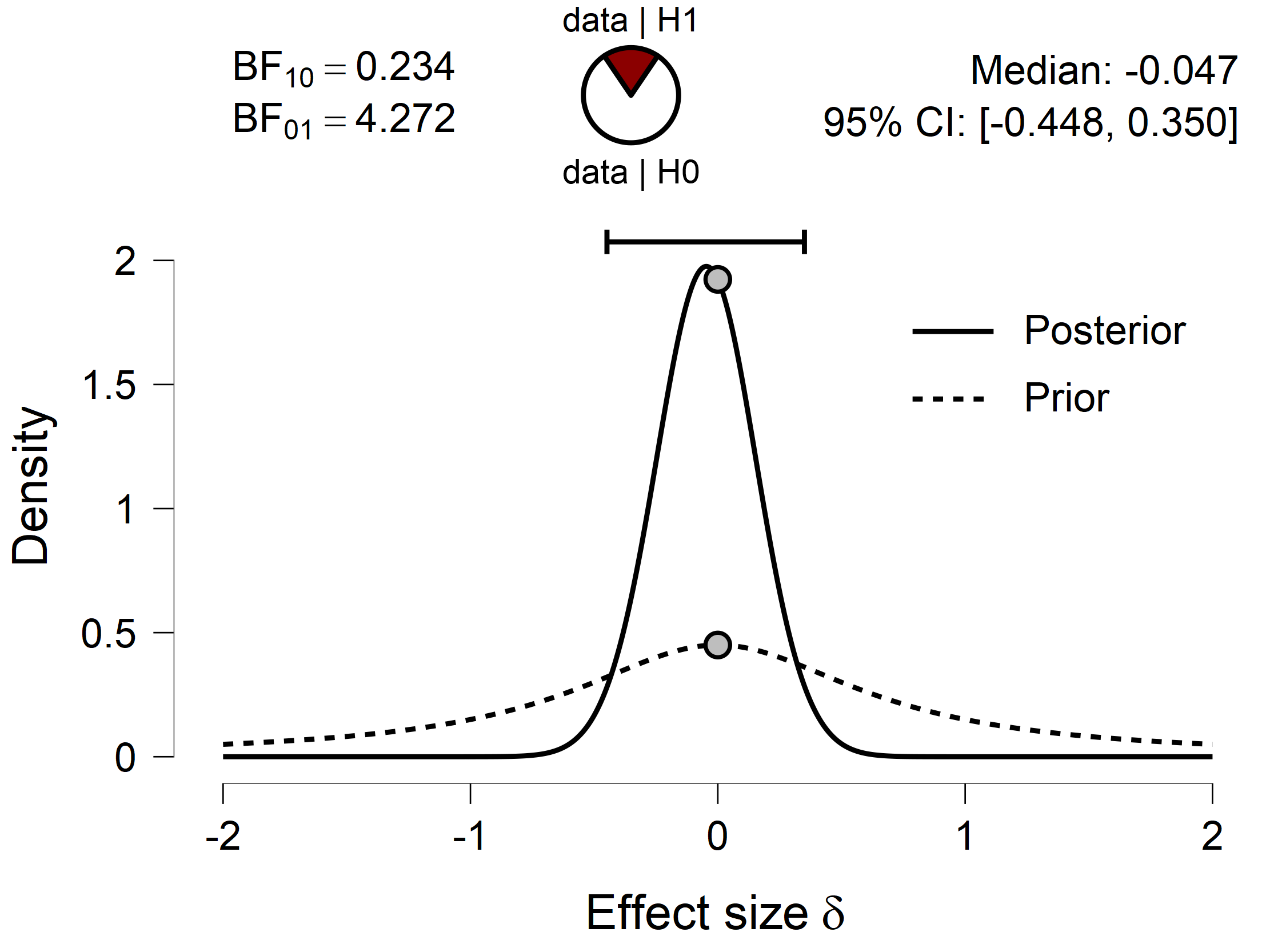


**Supplementary Figure 14.** Prior and posterior distribution of the effect size under H1 setting a default prior.

## PAA S1


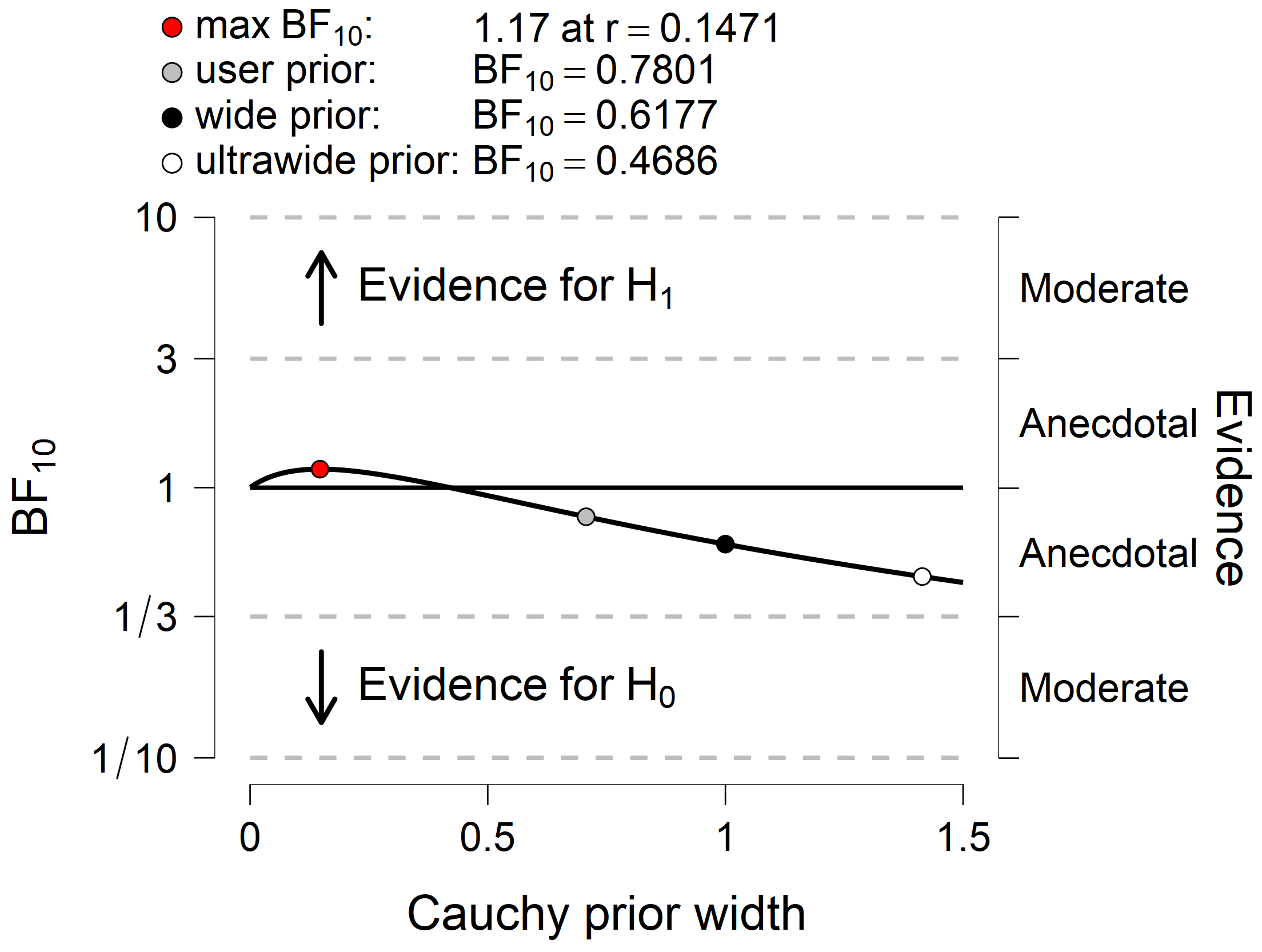


**Supplementary Figure 15.** Robustness analysis for the BF_10_ when comparing of the PAA in S1 against zero.


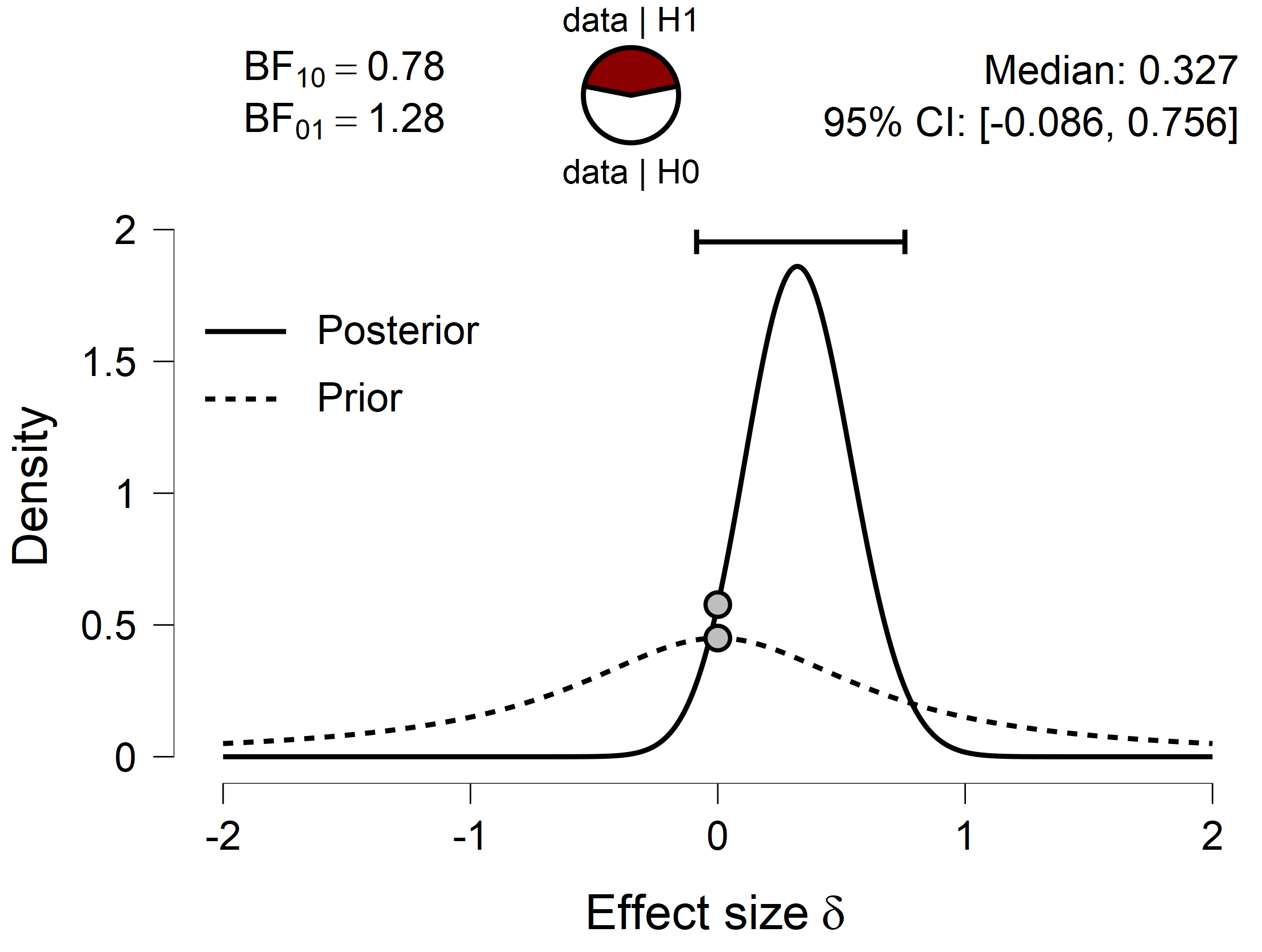


**Supplementary Figure 16.** Prior and posterior distribution of the effect size under H1 setting a default prior.

## PAA S2


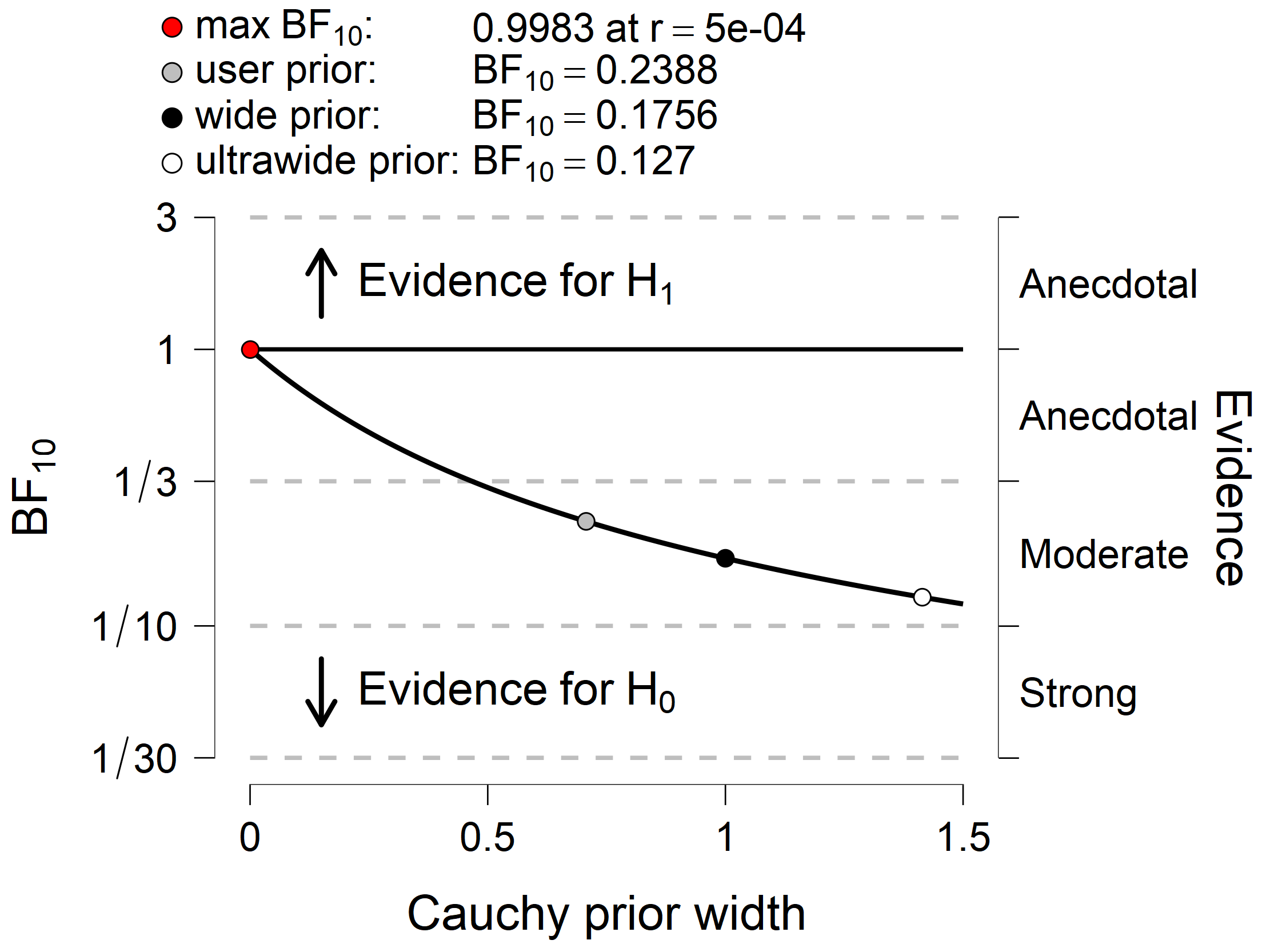


**Supplementary Figure 17.** Robustness analysis for the BF_10_ when comparing of the PAA in S2 against zero.


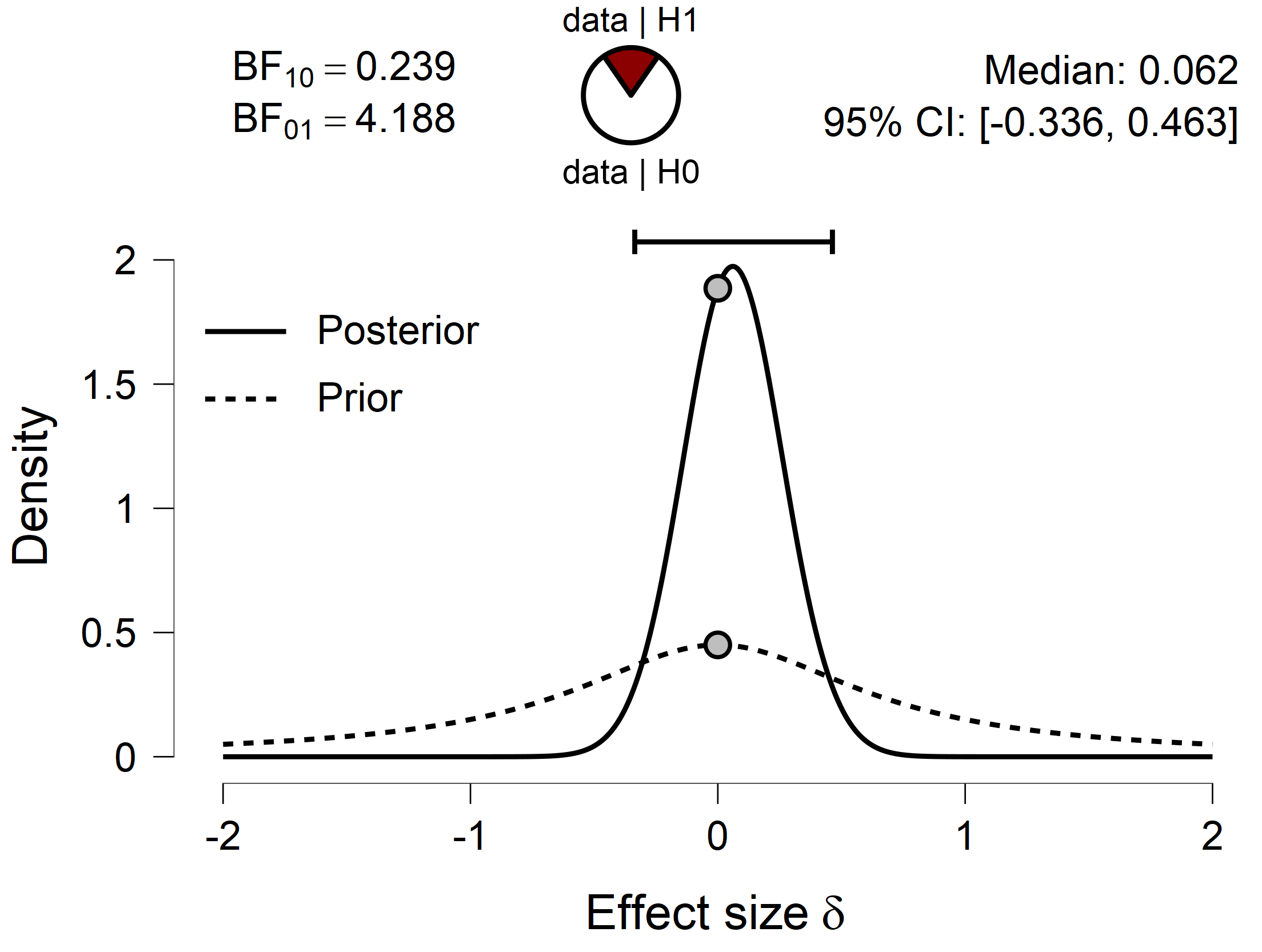


**Supplementary Figure 18.** Prior and posterior distribution of the effect size under H1 setting a default prior.

# Robustness analysis of the Bayesian Kendall’s Tau-B Correlation

## Attitude S1 – Percentage of Investment S1


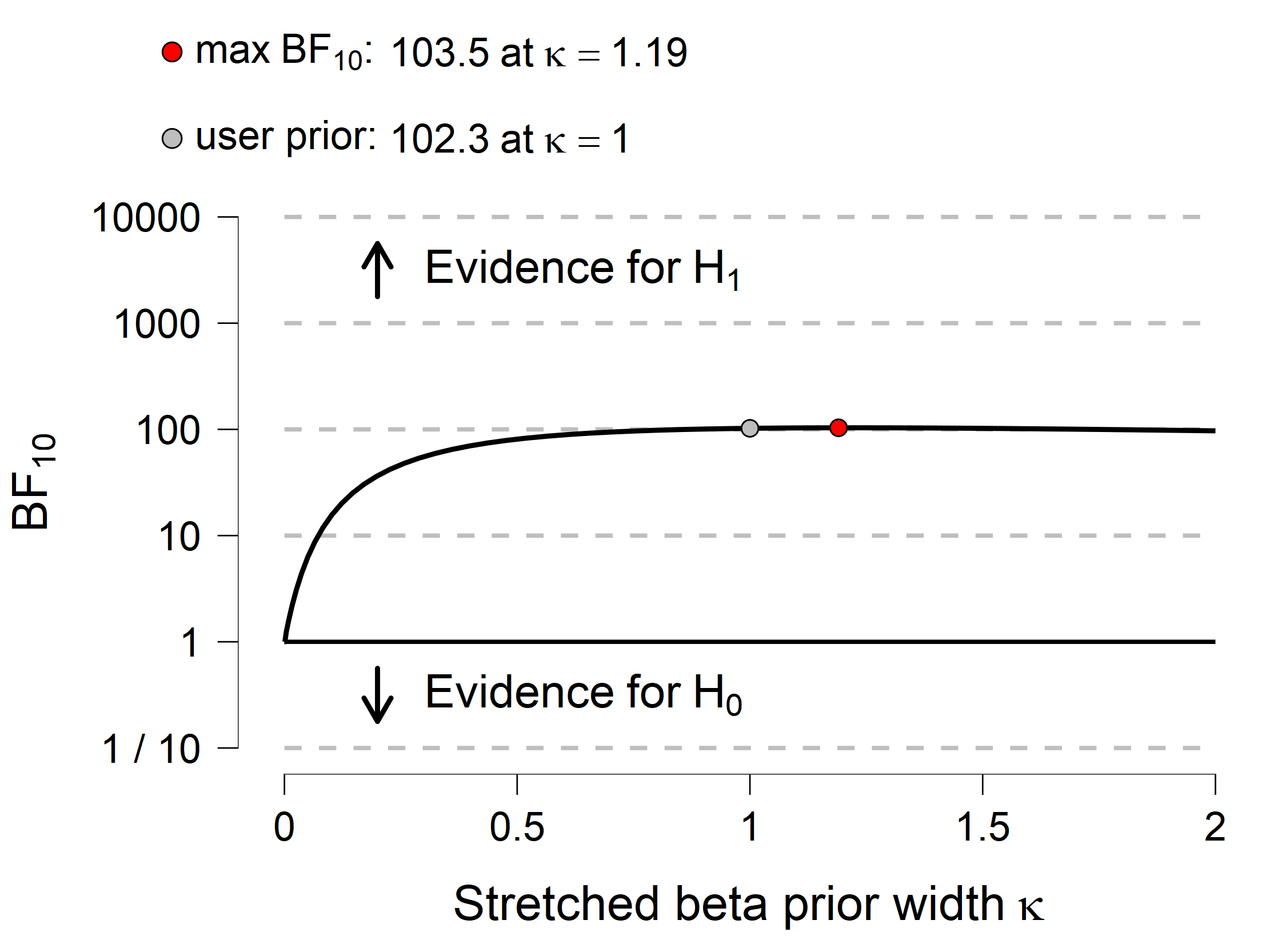


**Supplementary Figure 19.** Robustness analysis for the BF_10_ when correlating the attitude in S1 with the percentage of investment in S1. Maximum BF_10_ in red and Default BF_10_ in grey.


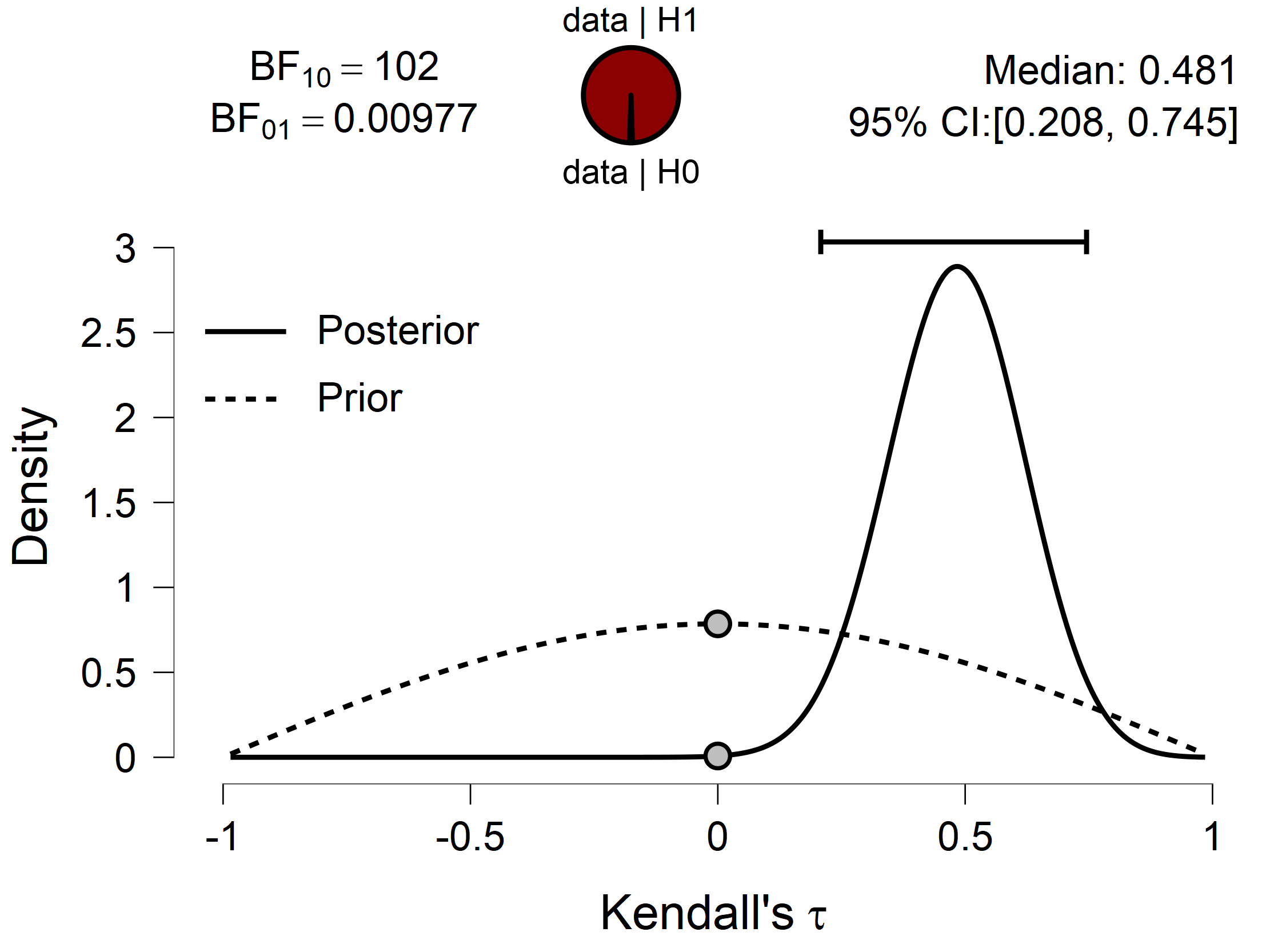


**Supplementary Figure 20.** Prior and posterior distribution of the effect size under H1 setting a default prior.

## Attitude S1 – FAA S1


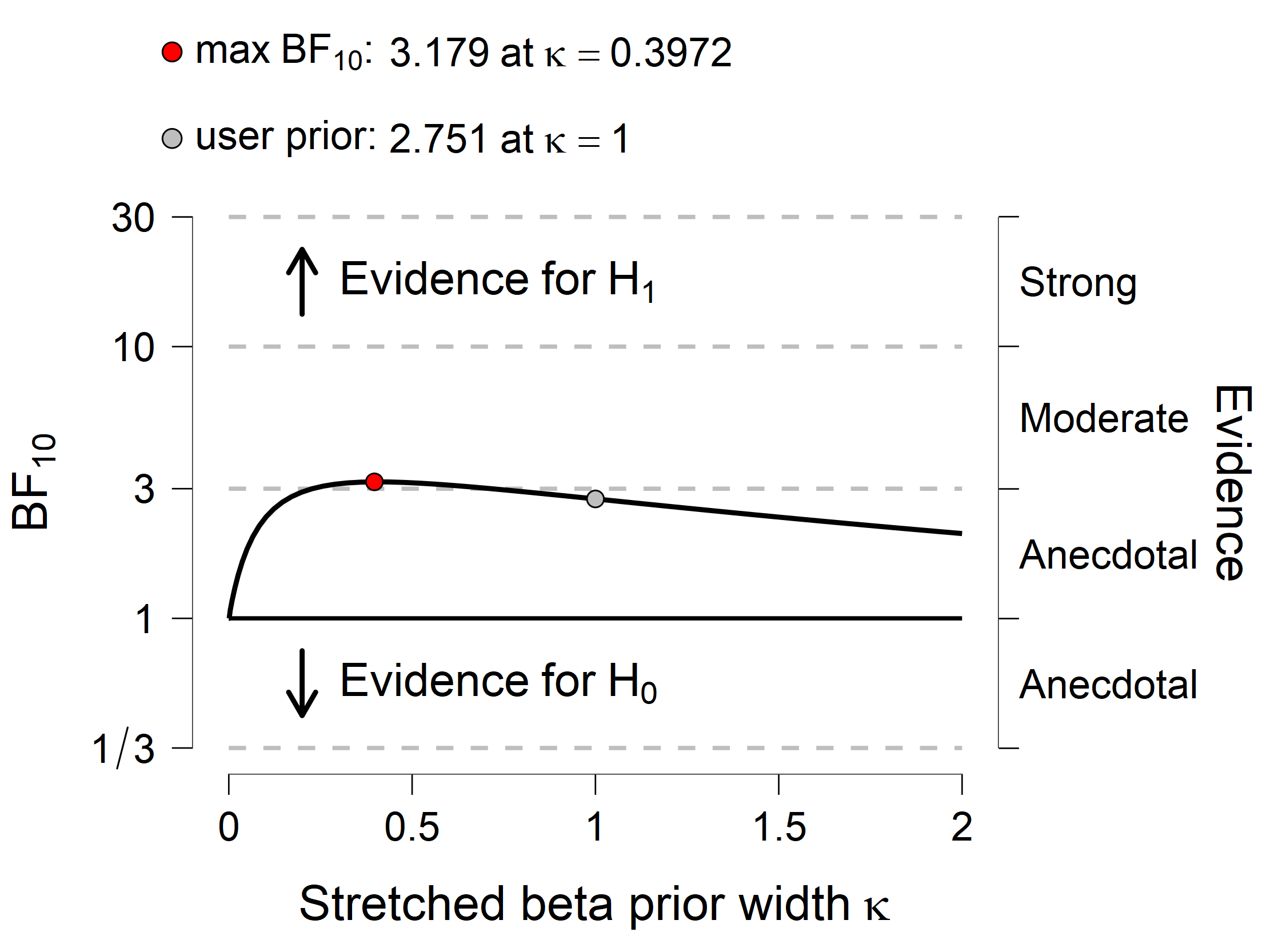


**Supplementary Figure 21.** Robustness analysis for the BF_10_ when correlating the attitude in S1 with the FAA in S1. Maximum BF_10_ in red and Default BF_10_ in grey.


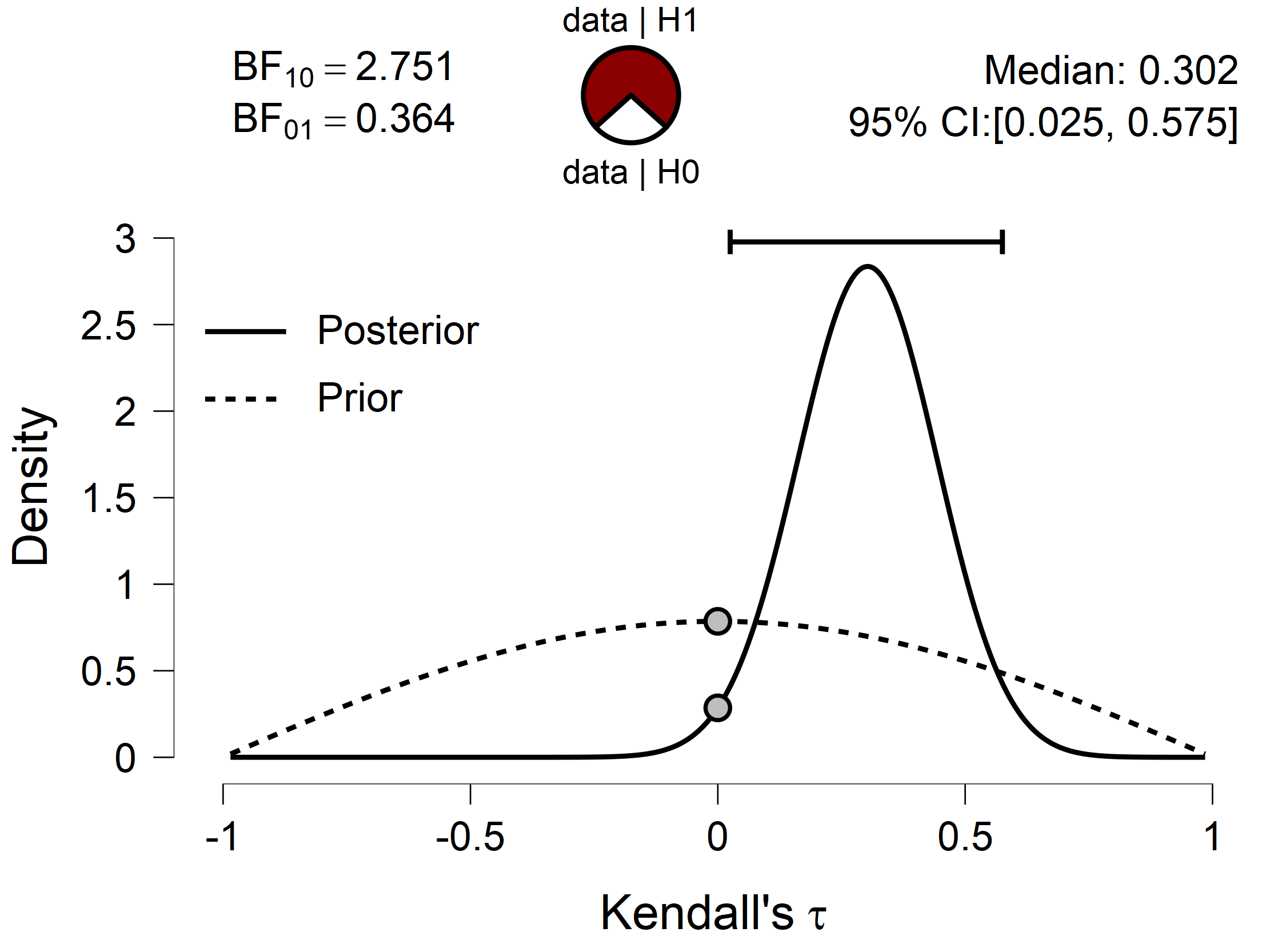


**Supplementary Figure 22.** Prior and posterior distribution of the effect size under H1 setting a default prior.

## Attitude S1 – PAA S1


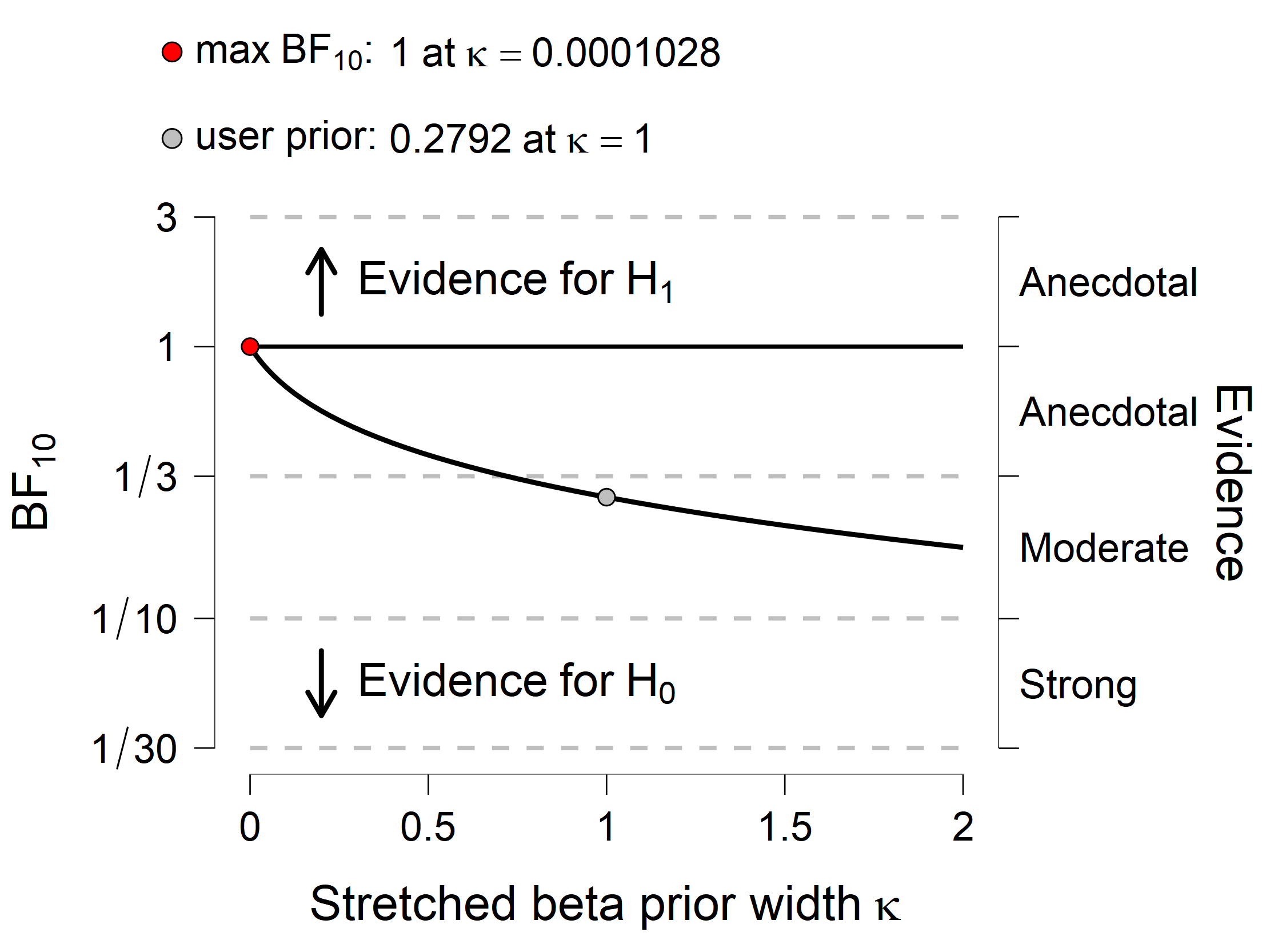


**Supplementary Figure 23.** Robustness analysis for the BF_10_ when correlating the attitude in S1 with the PAA in S1. Maximum BF_10_ in red and Default BF_10_ in grey.


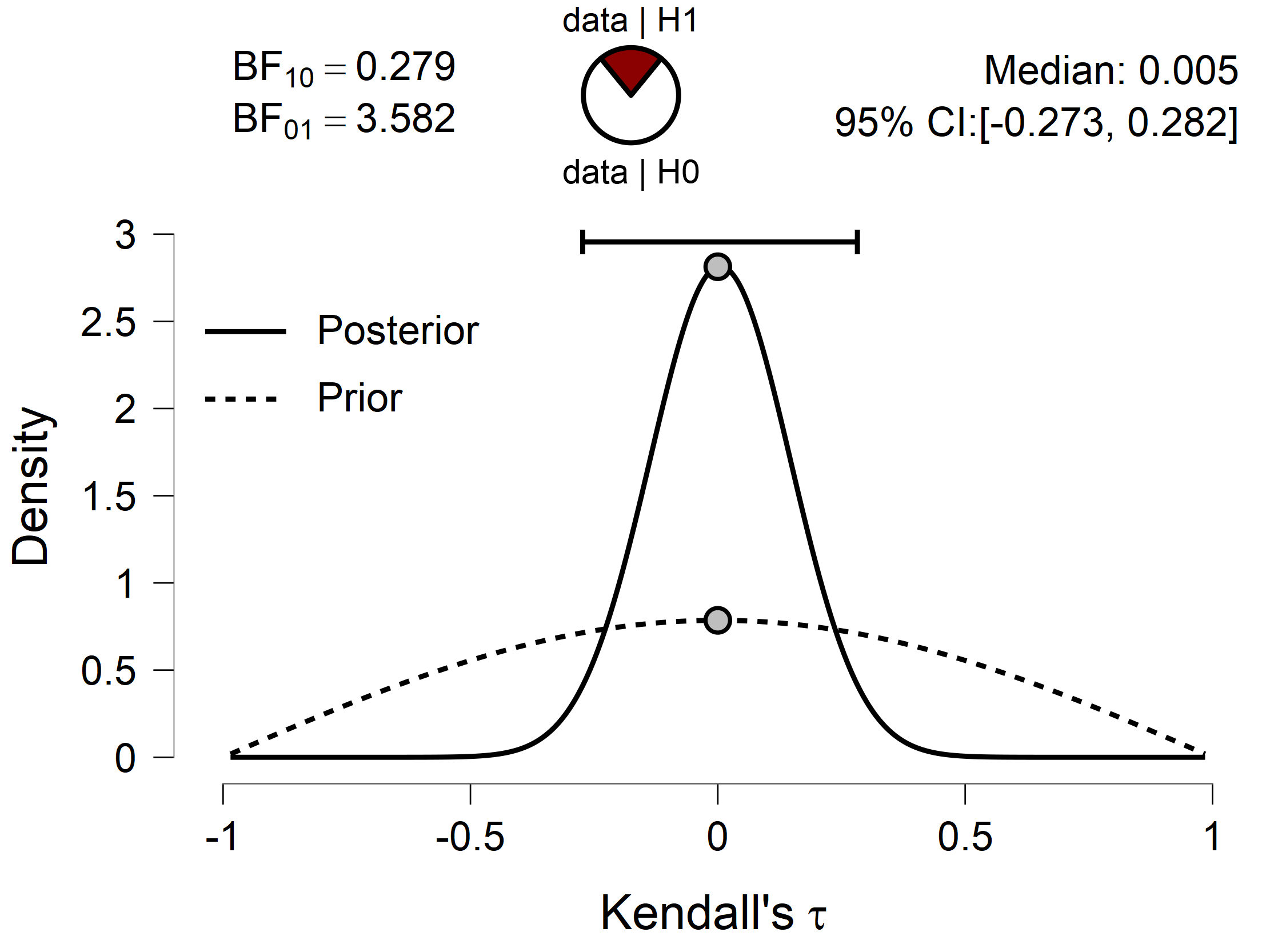


**Supplementary Figure 24.** Prior and posterior distribution of the effect size under H1 setting a default prior.

## Attitude S1 – Pupil Diameter S1


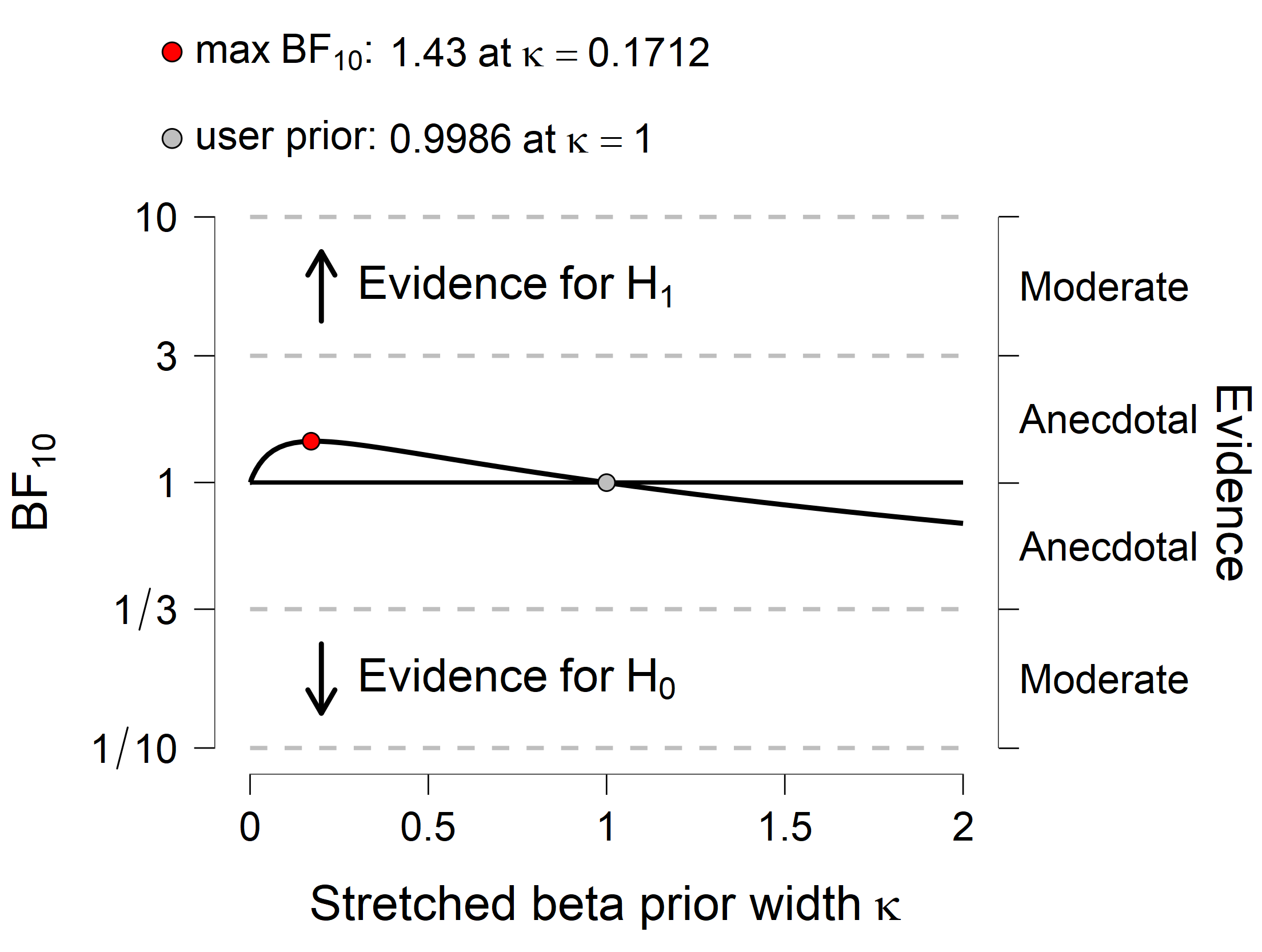


**Supplementary Figure 25.** Robustness analysis for the BF_10_ when correlating the attitude in S1 with the Pupil Diameter in S1. Maximum BF_10_ in red and Default BF_10_ in grey.


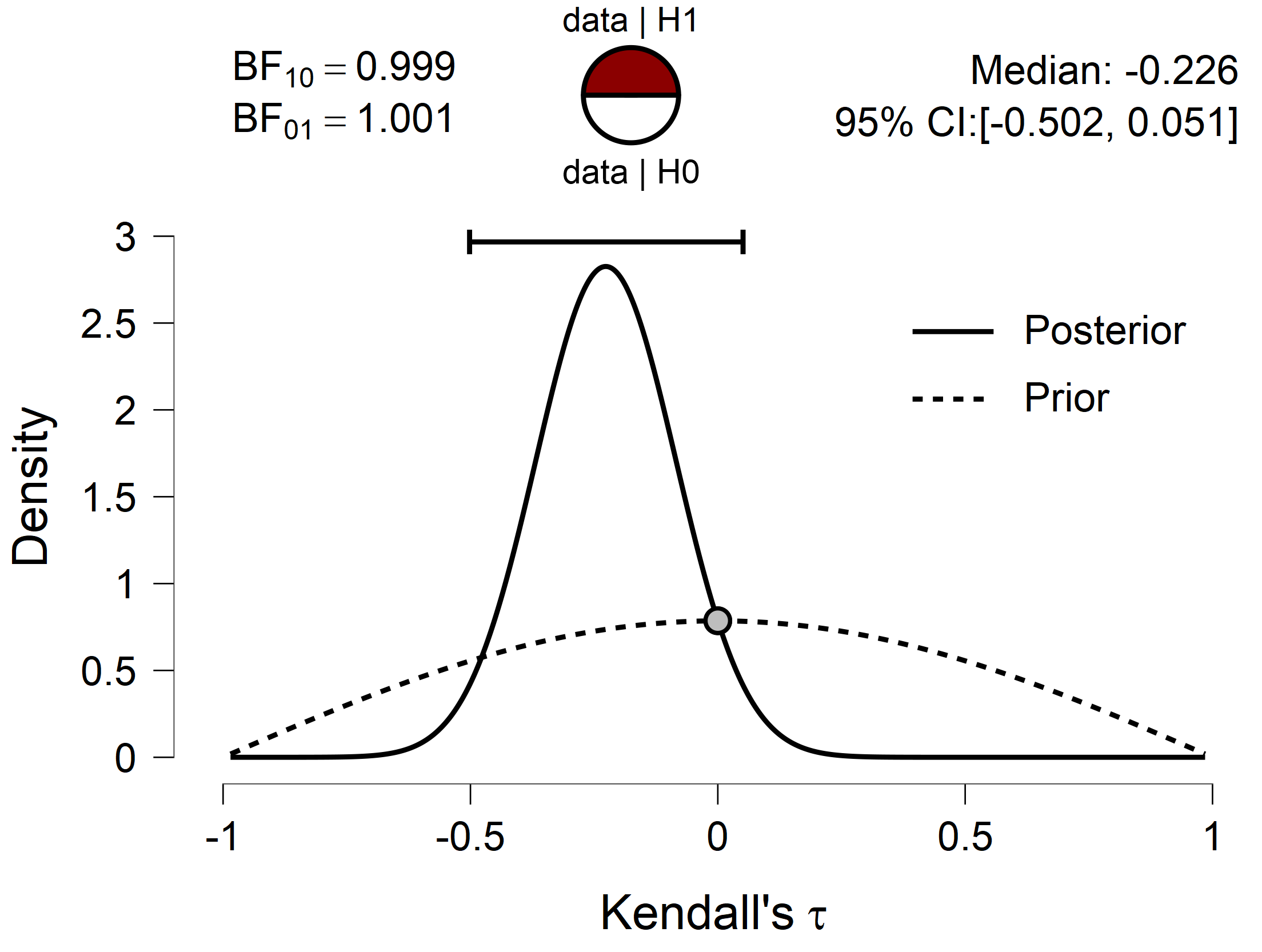


**Supplementary Figure 26.** Prior and posterior distribution of the effect size under H1 setting a default prior.

## Percentage of Investment S1 – FAA S1


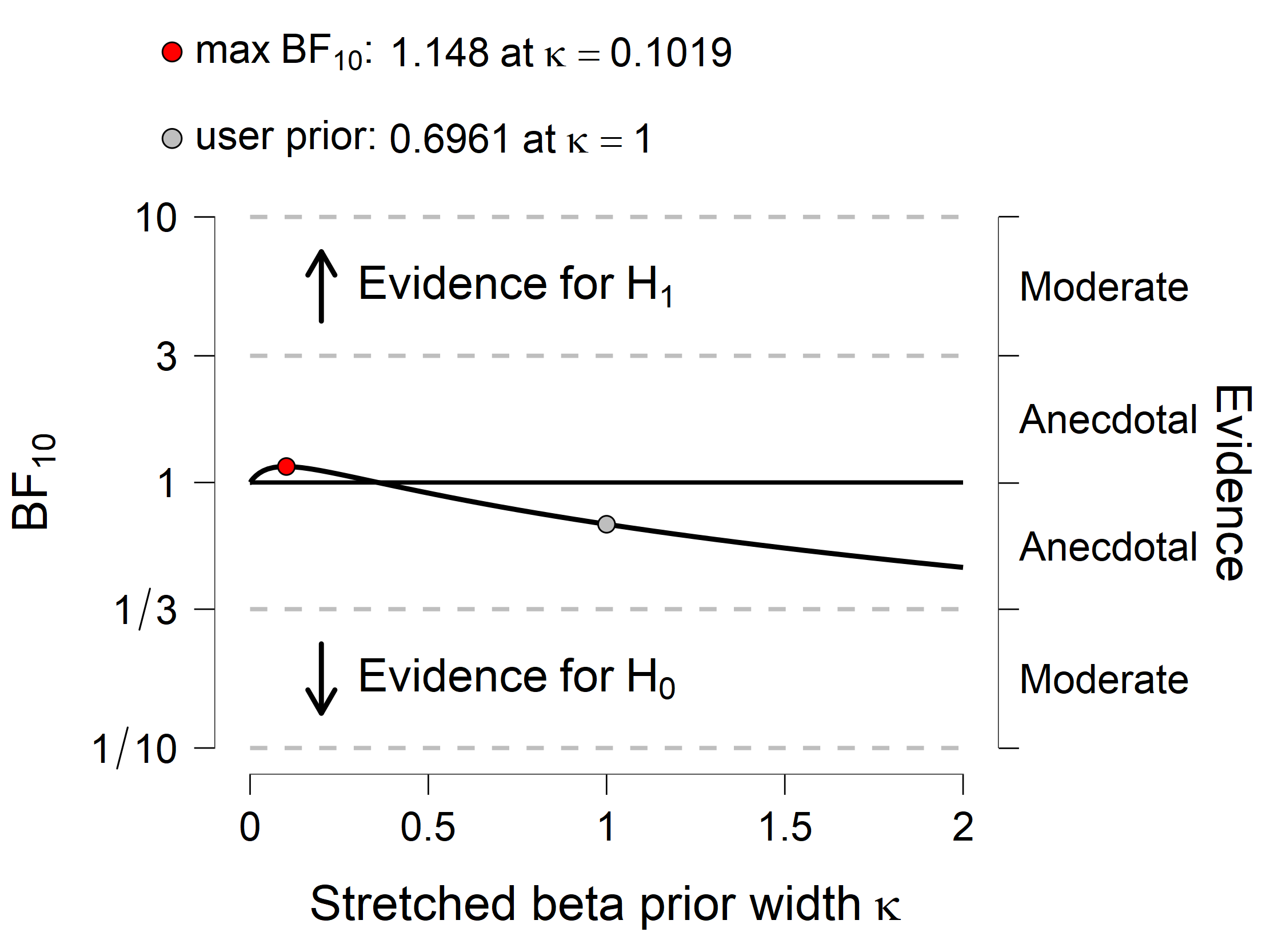


**Supplementary Figure 27.** Robustness analysis for the BF_10_ when correlating the percentage of investment in S1 with the FAA in S1. Maximum BF_10_ in red and Default BF_10_ in grey.


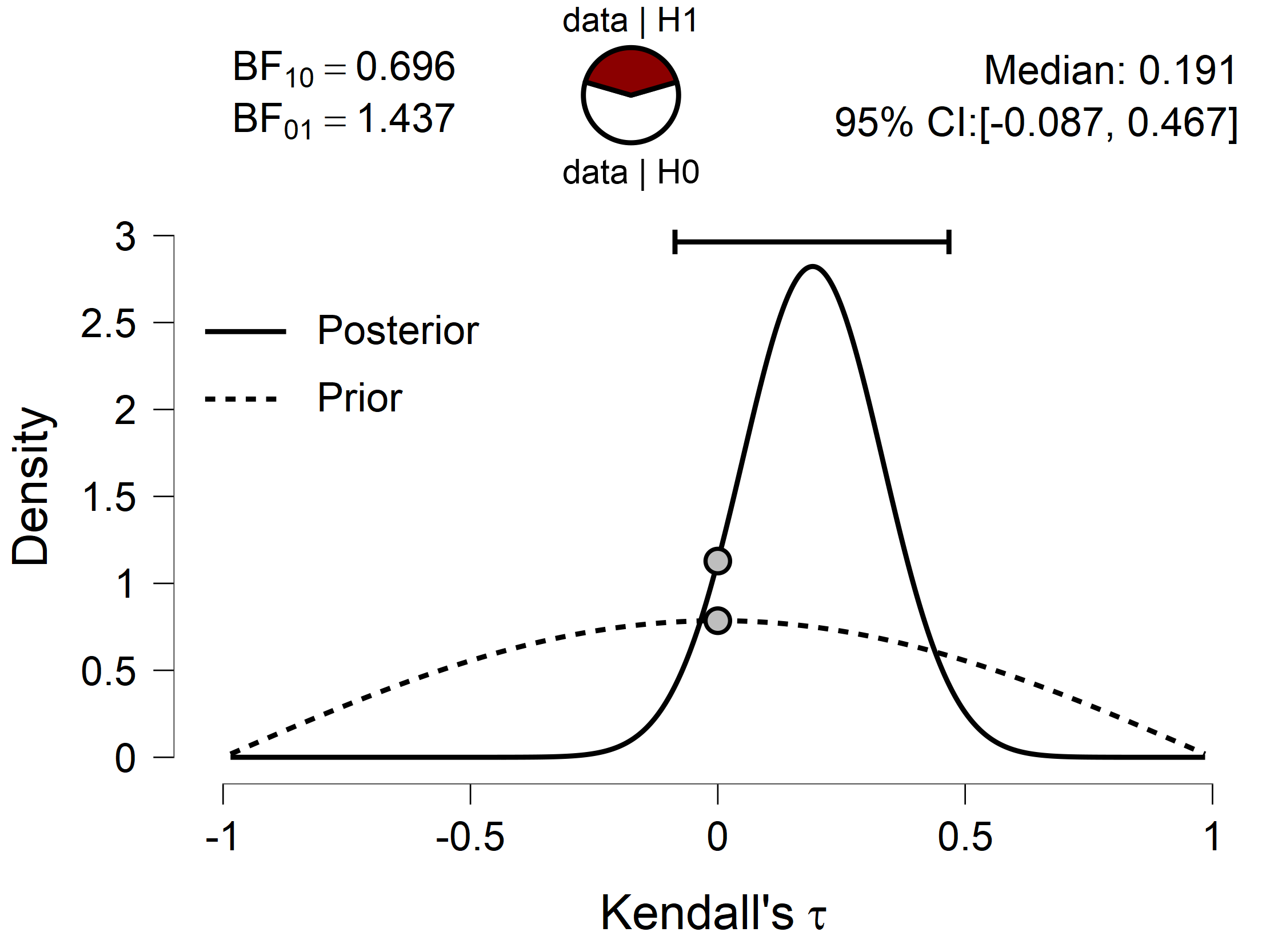


**Supplementary Figure 28.** Prior and posterior distribution of the effect size under H1 setting a default prior.

## Percentage of Investment S1 – PAA S1


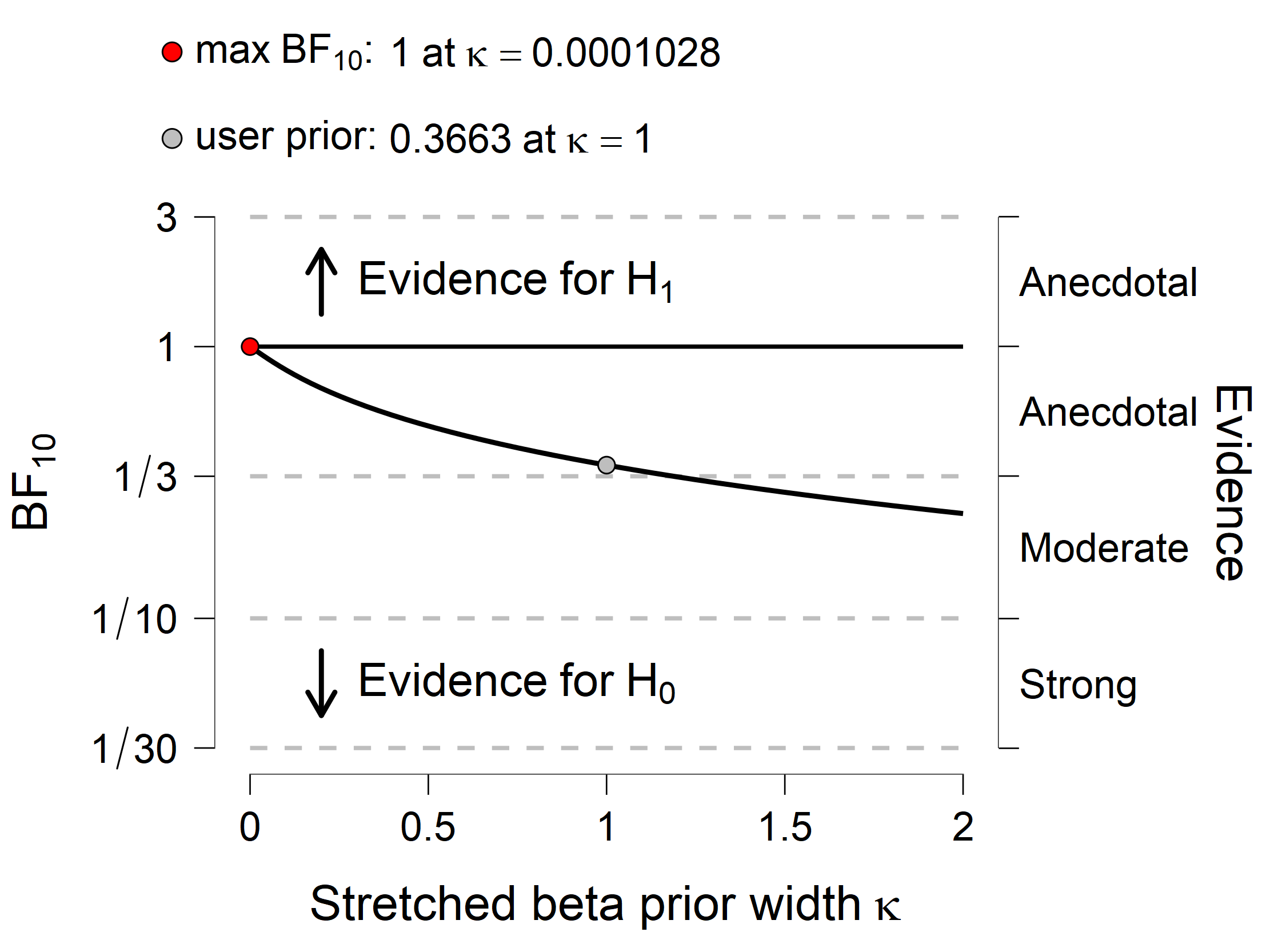


**Supplementary Figure 29.** Robustness analysis for the BF_10_ when correlating the percentage of investment in S1 with the PAA in S1. Maximum BF_10_ in red and Default BF_10_ in grey.


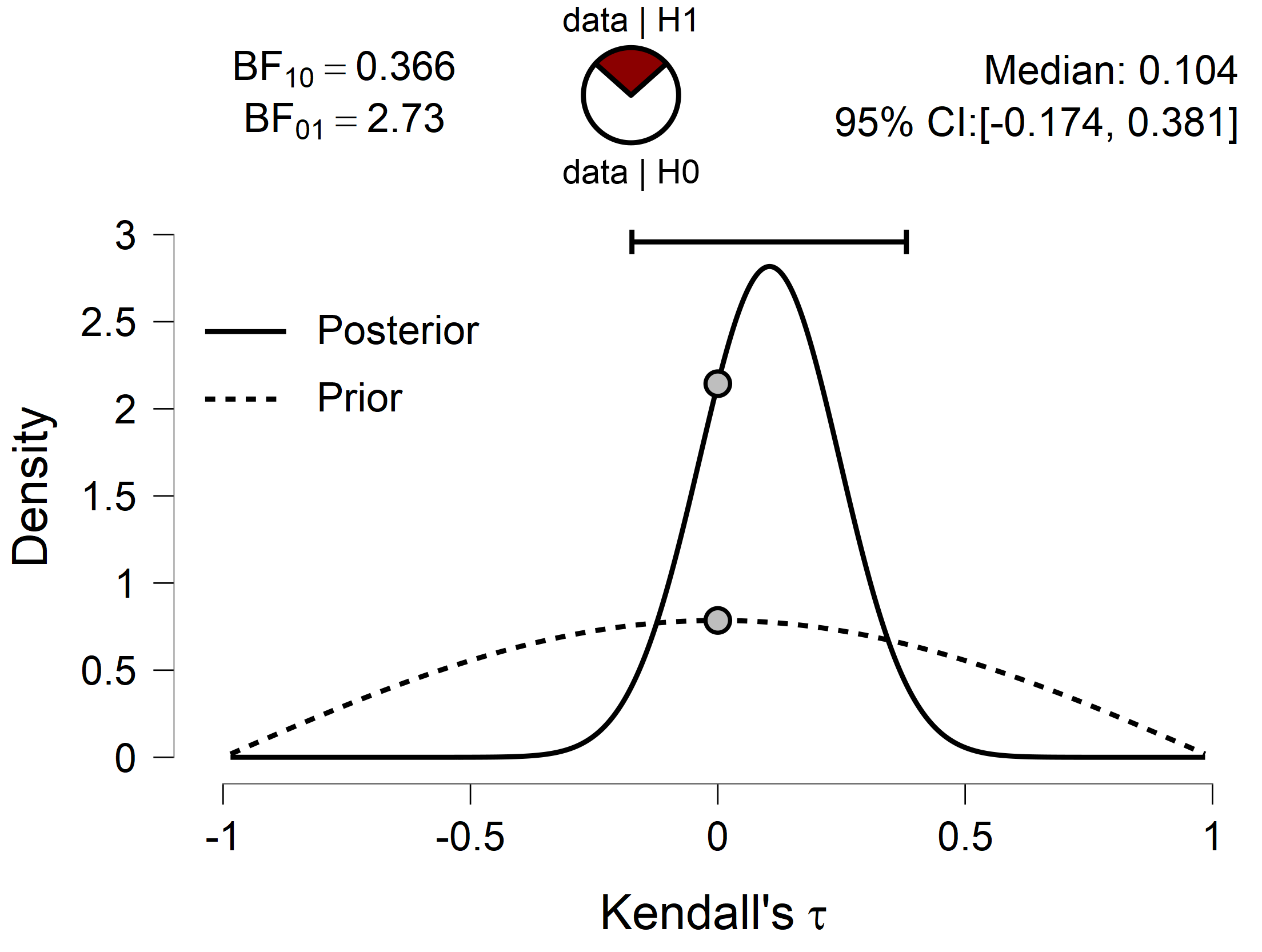


**Supplementary Figure 30.** Prior and posterior distribution of the effect size under H1 setting a default prior.

## Percentage of Investment S1 – Pupil Diameter S1


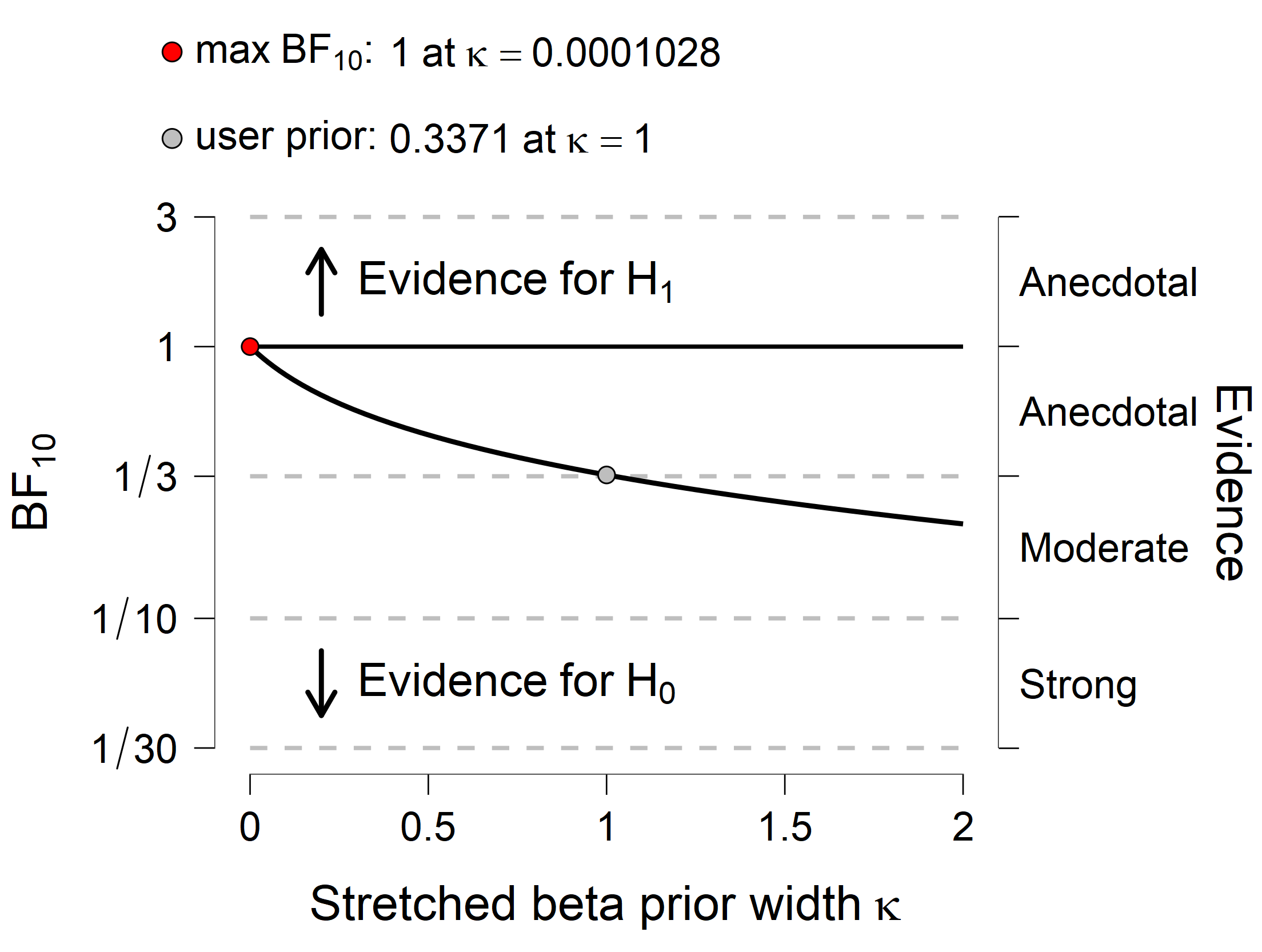


**Supplementary Figure 31.** Robustness analysis for the BF_10_ when correlating the percentage of investment in S1 with the Pupil Diameter in S1. Maximum BF_10_ in red and Default BF_10_ in grey.


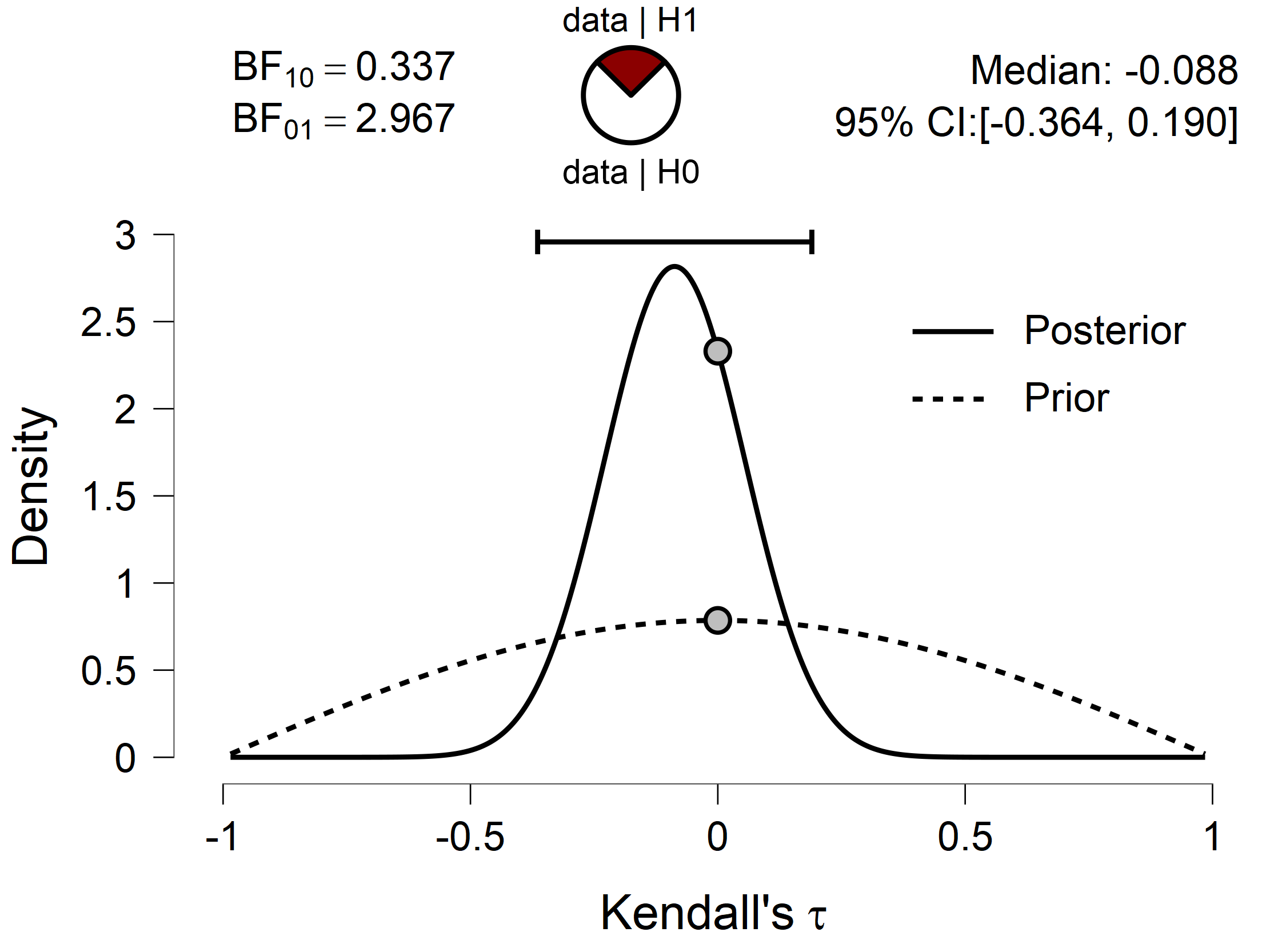


**Supplementary Figure 32.** Prior and posterior distribution of the effect size under H1 setting a default prior.

## Attitude S2 – Percentage of Investment S2


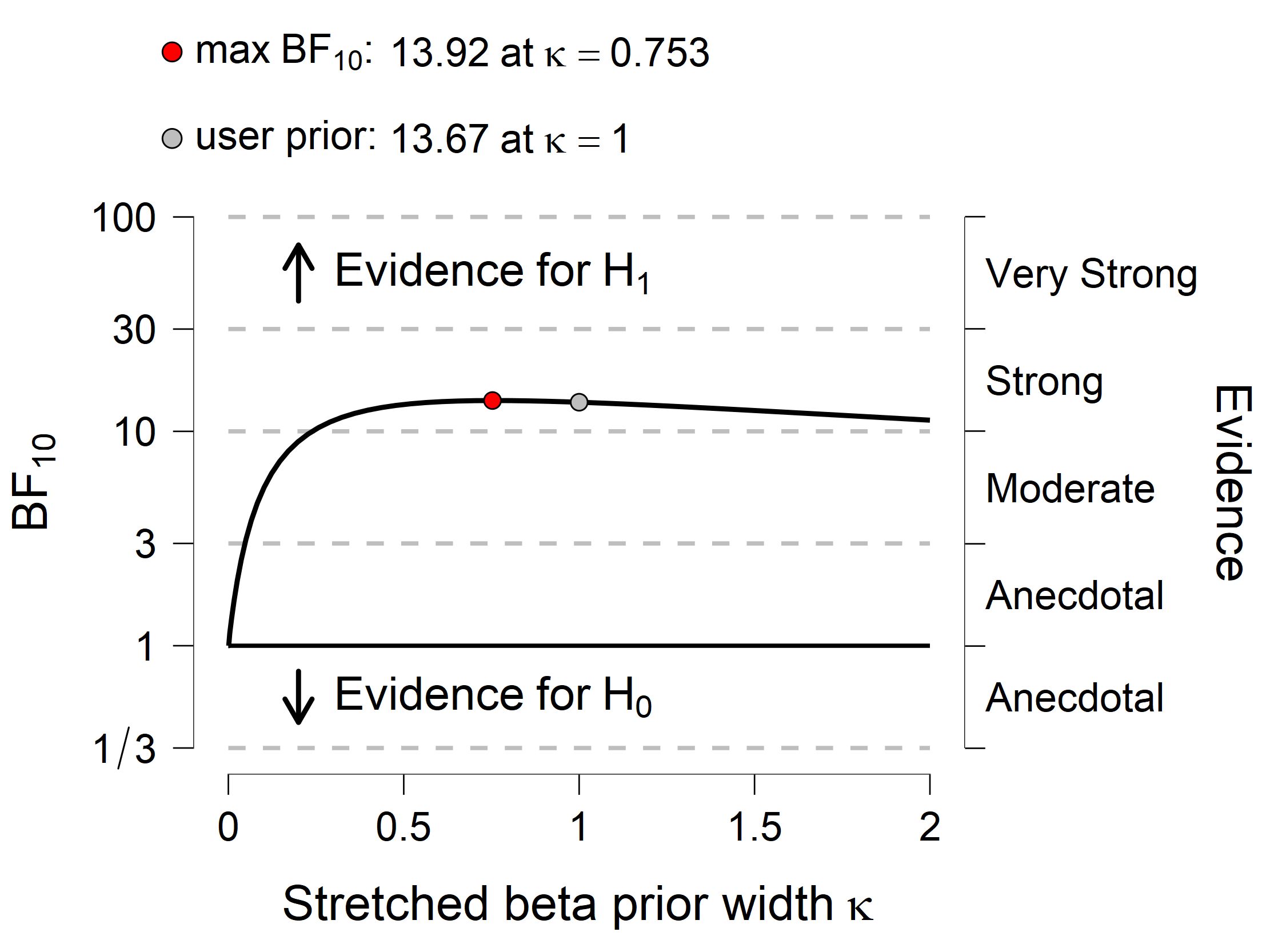


**Supplementary Figure 33.** Robustness analysis for the BF_10_ when correlating the attitude in S2 with the percentage of investment in S2. Maximum BF_10_ in red and Default BF_10_ in grey.


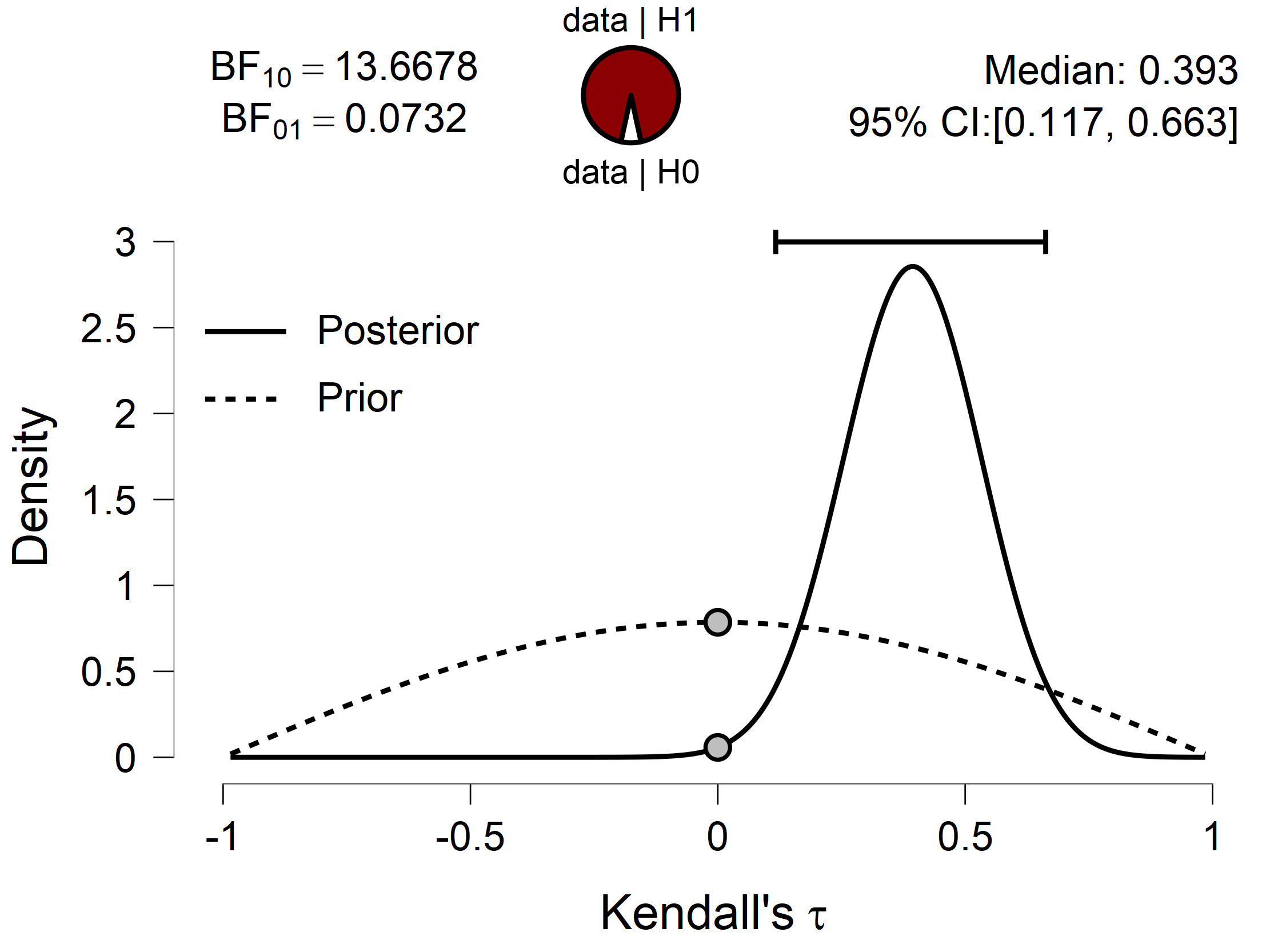


**Supplementary Figure 34.** Prior and posterior distribution of the effect size under H1 setting a default prior.

## Attitude S2 – FAA S2


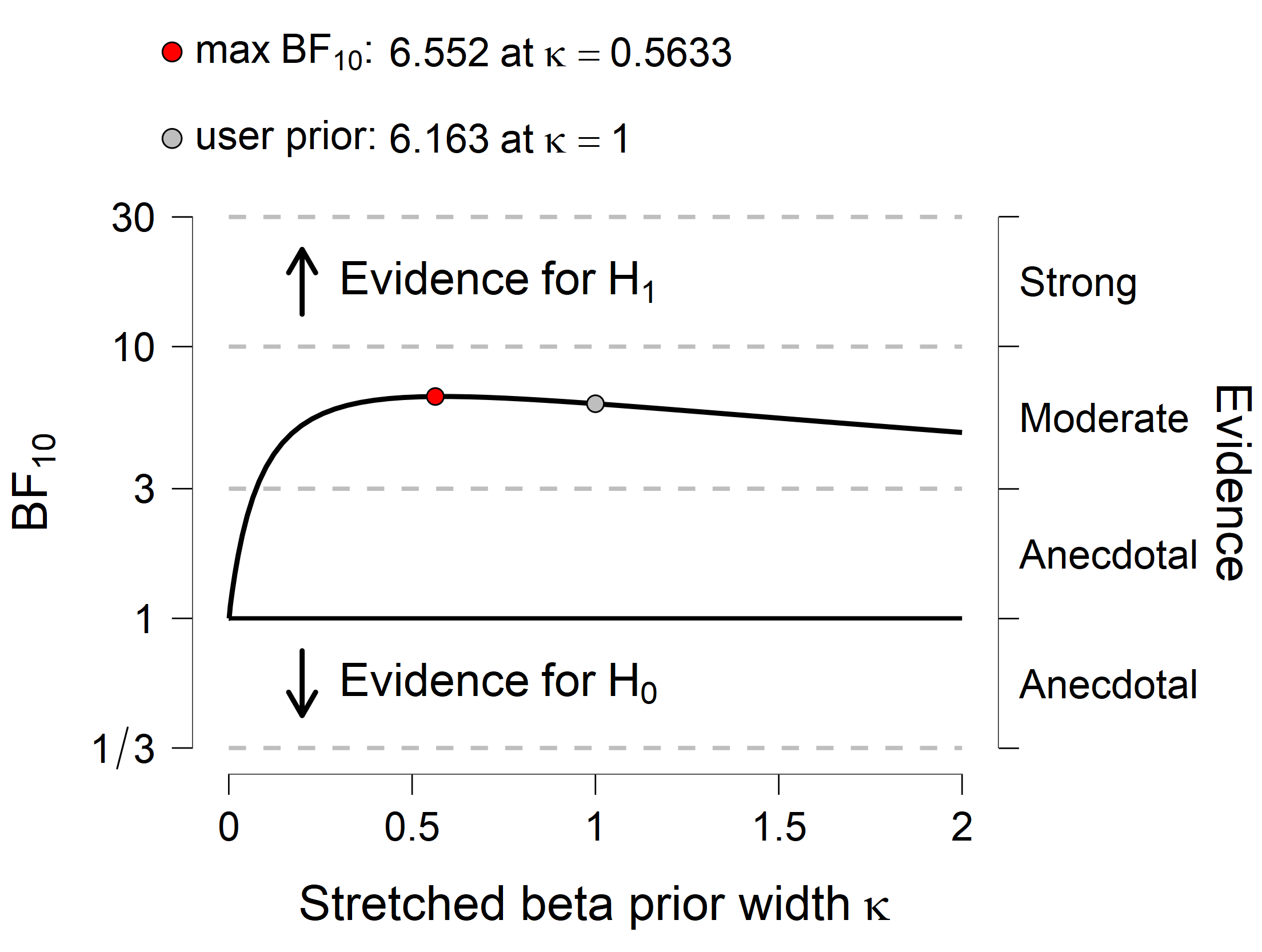


**Supplementary Figure 35.** Robustness analysis for the BF_10_ when correlating the attitude in S2 with the FAA in S2. Maximum BF_10_ in red and Default BF_10_ in grey.


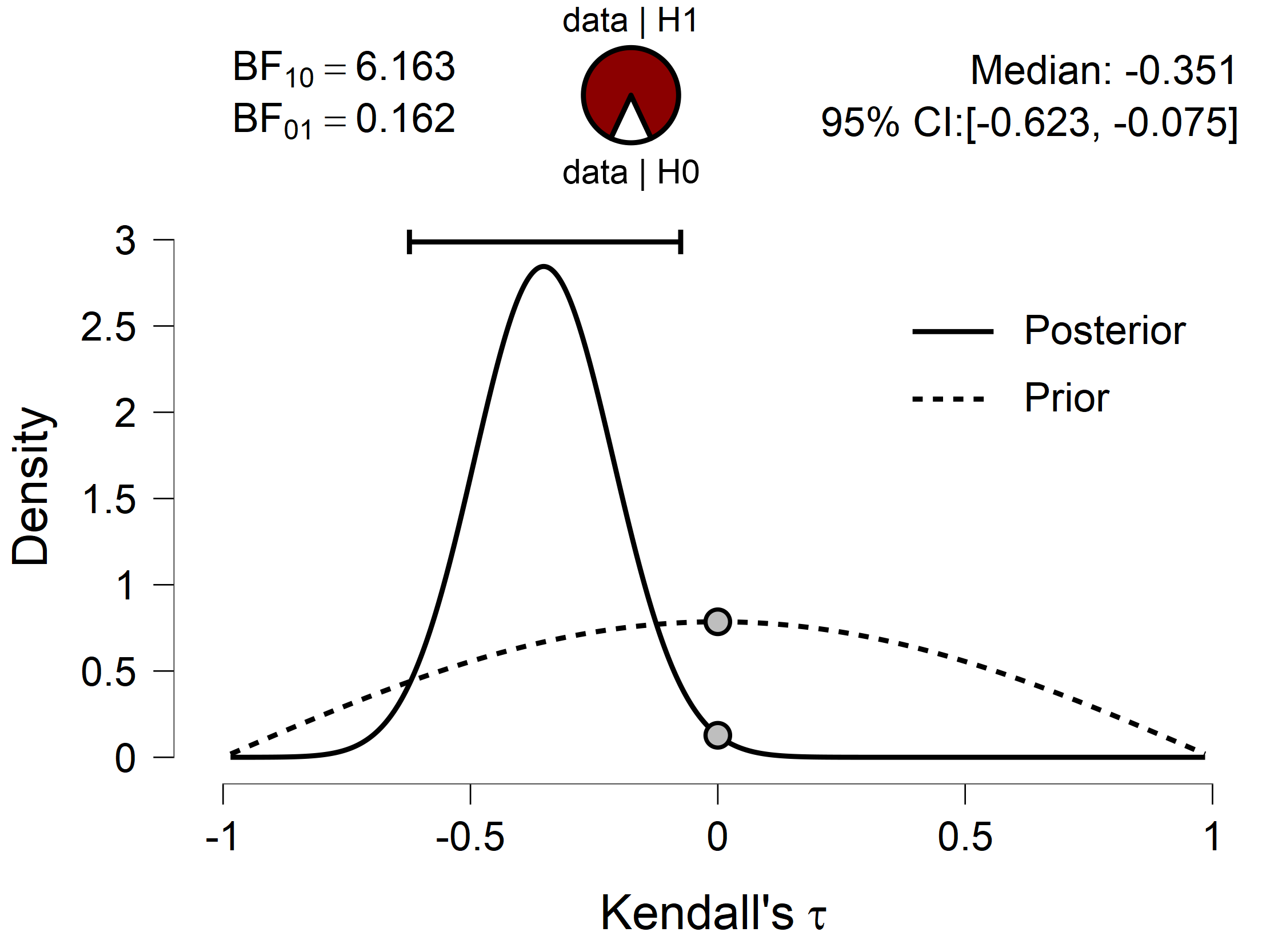


**Supplementary Figure 36.** Prior and posterior distribution of the effect size under H1 setting a default prior.

## Attitude S2 – PAA S2


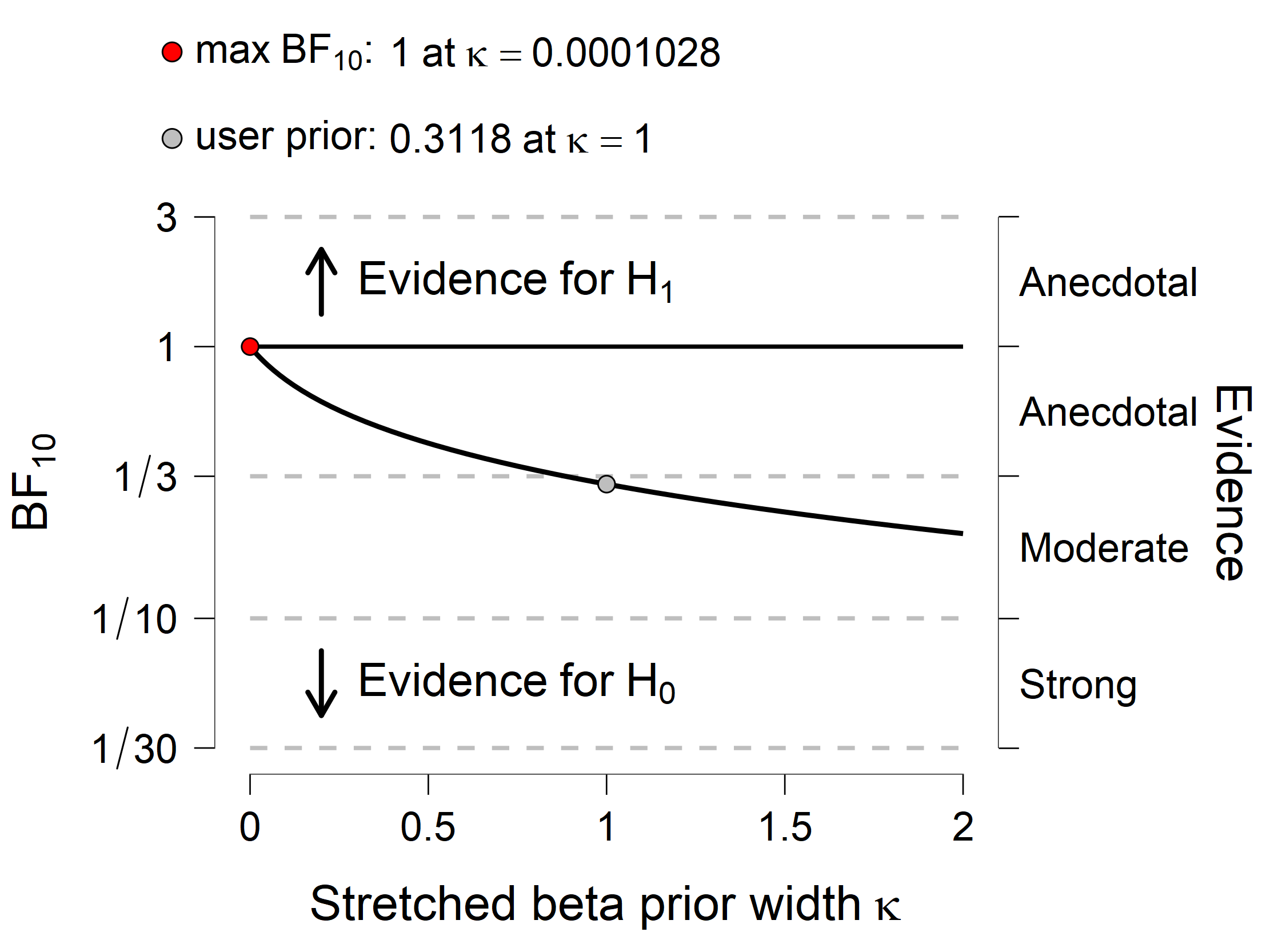


**Supplementary Figure 37.** Robustness analysis for the BF_10_ when correlating the attitude in S2 with the PAA in S2. Maximum BF_10_ in red and Default BF_10_ in grey.


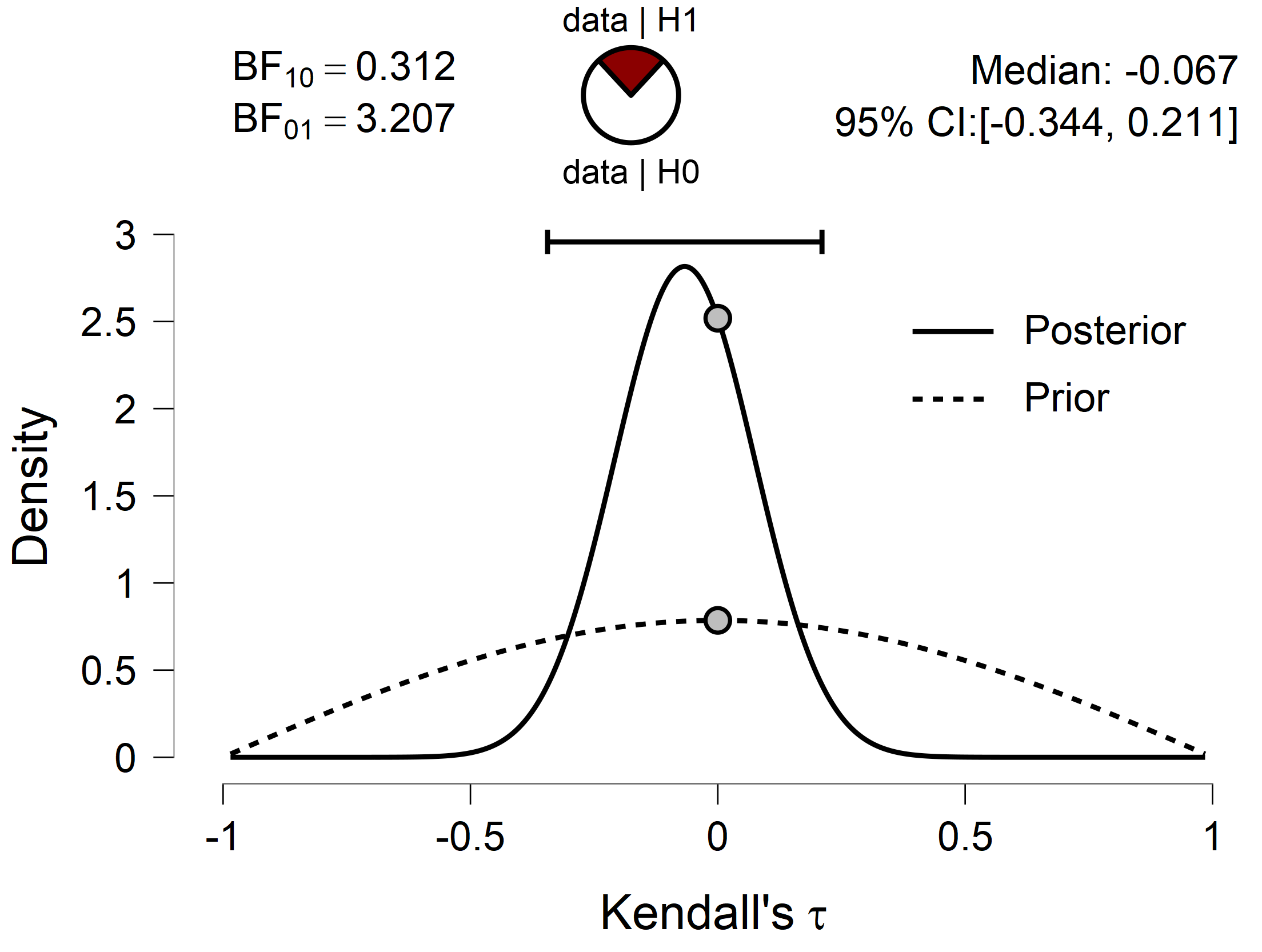


**Supplementary Figure 38.** Prior and posterior distribution of the effect size under H1 setting a default prior.

## Attitude S2 – Pupil Diameter S2


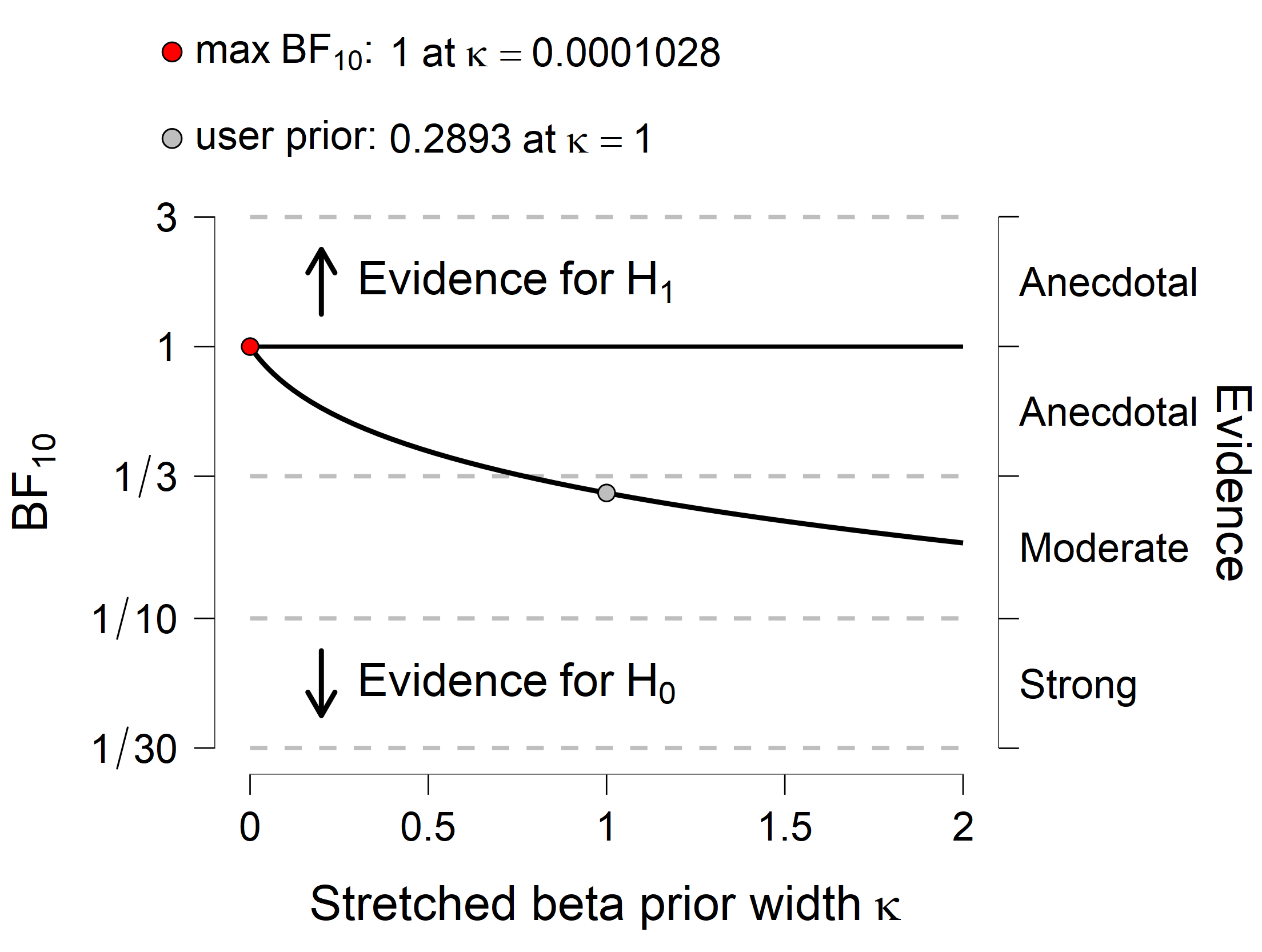


**Supplementary Figure 39.** Robustness analysis for the BF_10_ when correlating the attitude in S2 with the pupil diameter in S2. Maximum BF_10_ in red and Default BF_10_ in grey.


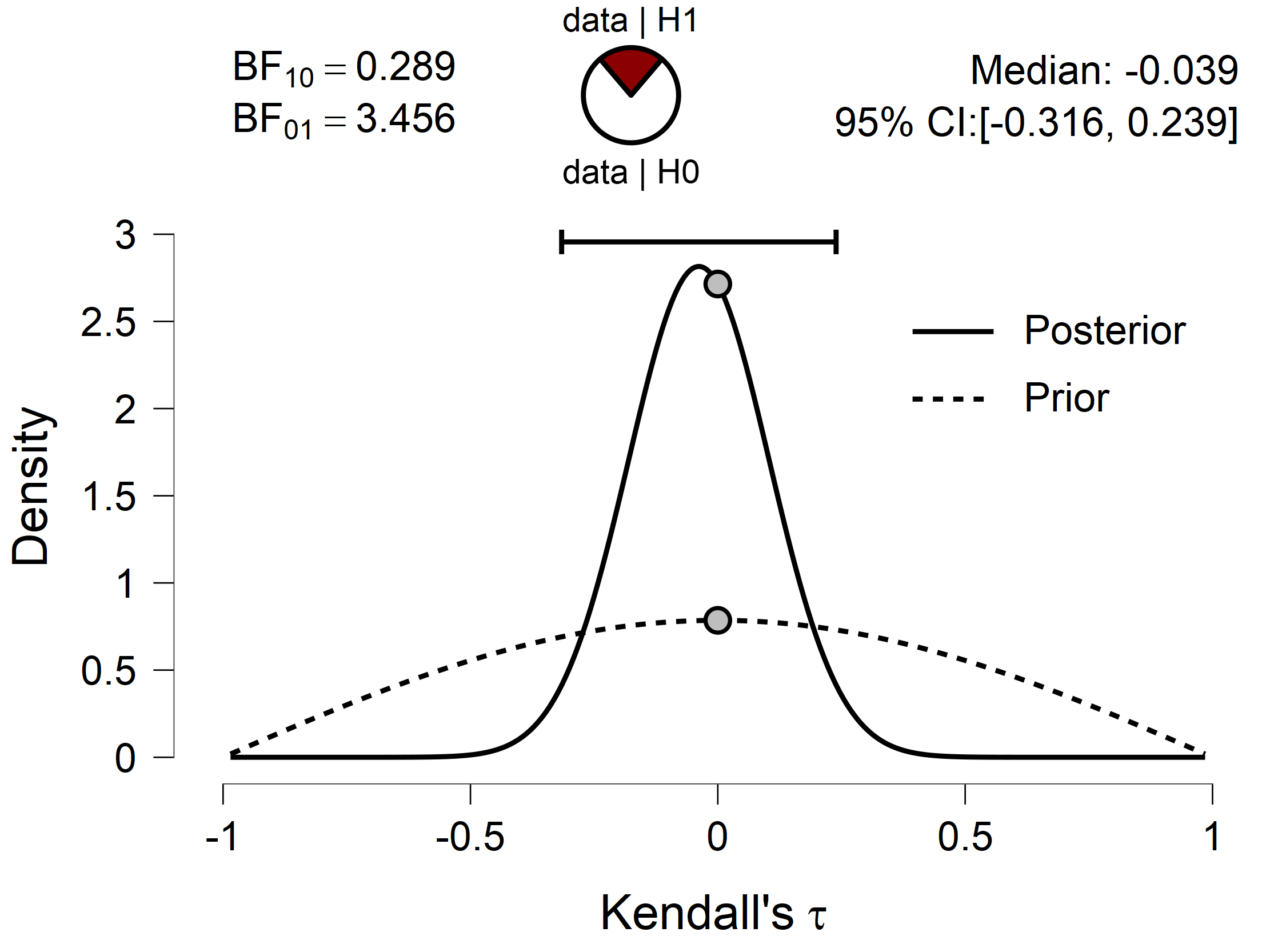


**Supplementary Figure 40.** Prior and posterior distribution of the effect size under H1 setting a default prior.

## Percentage of Investment S2 – FAA S2


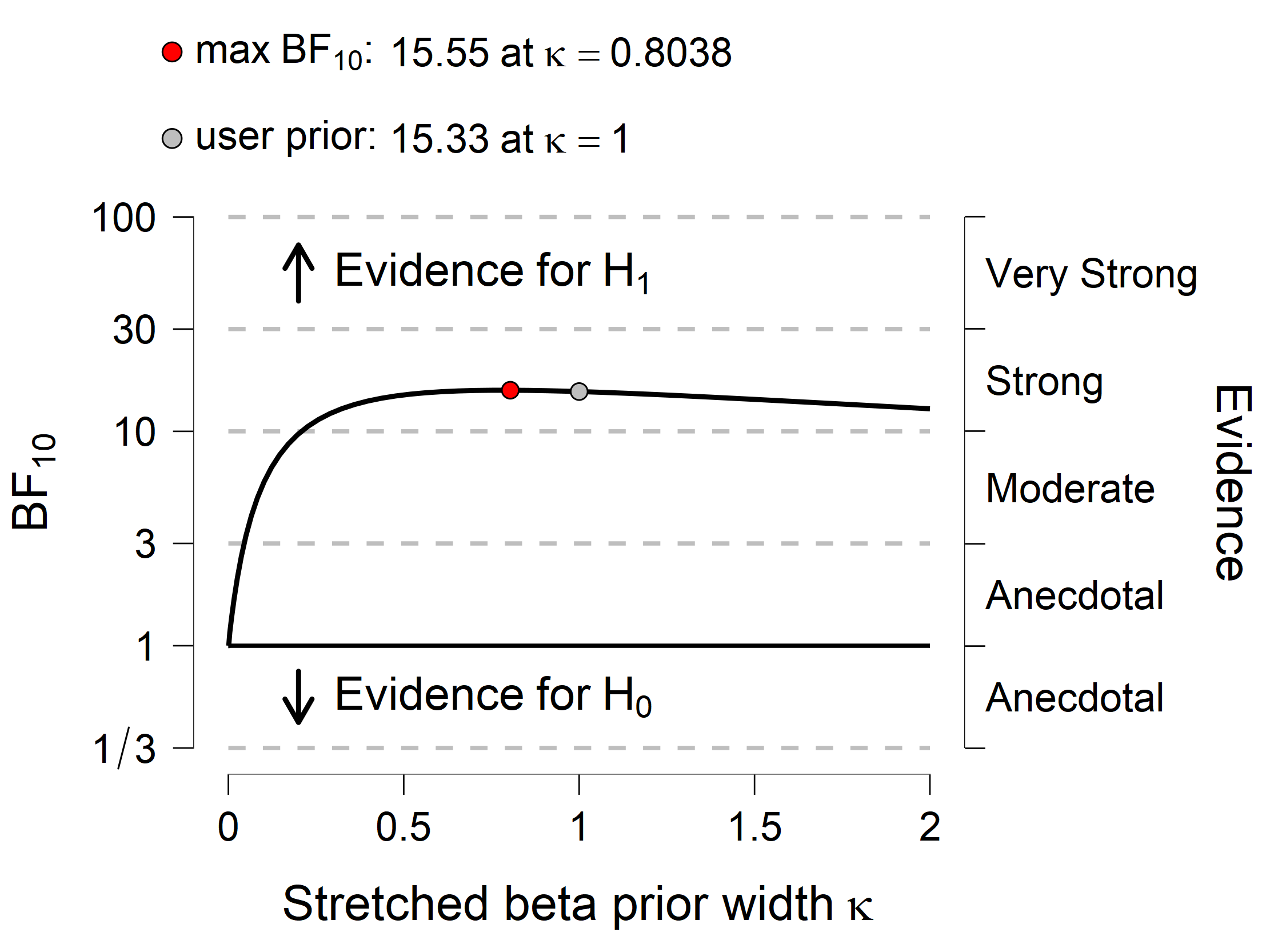


**Supplementary Figure 41.** Robustness analysis for the BF_10_ when correlating the percentage of investment in S2 with the FAA in S2. Maximum BF_10_ in red and Default BF_10_ in grey.


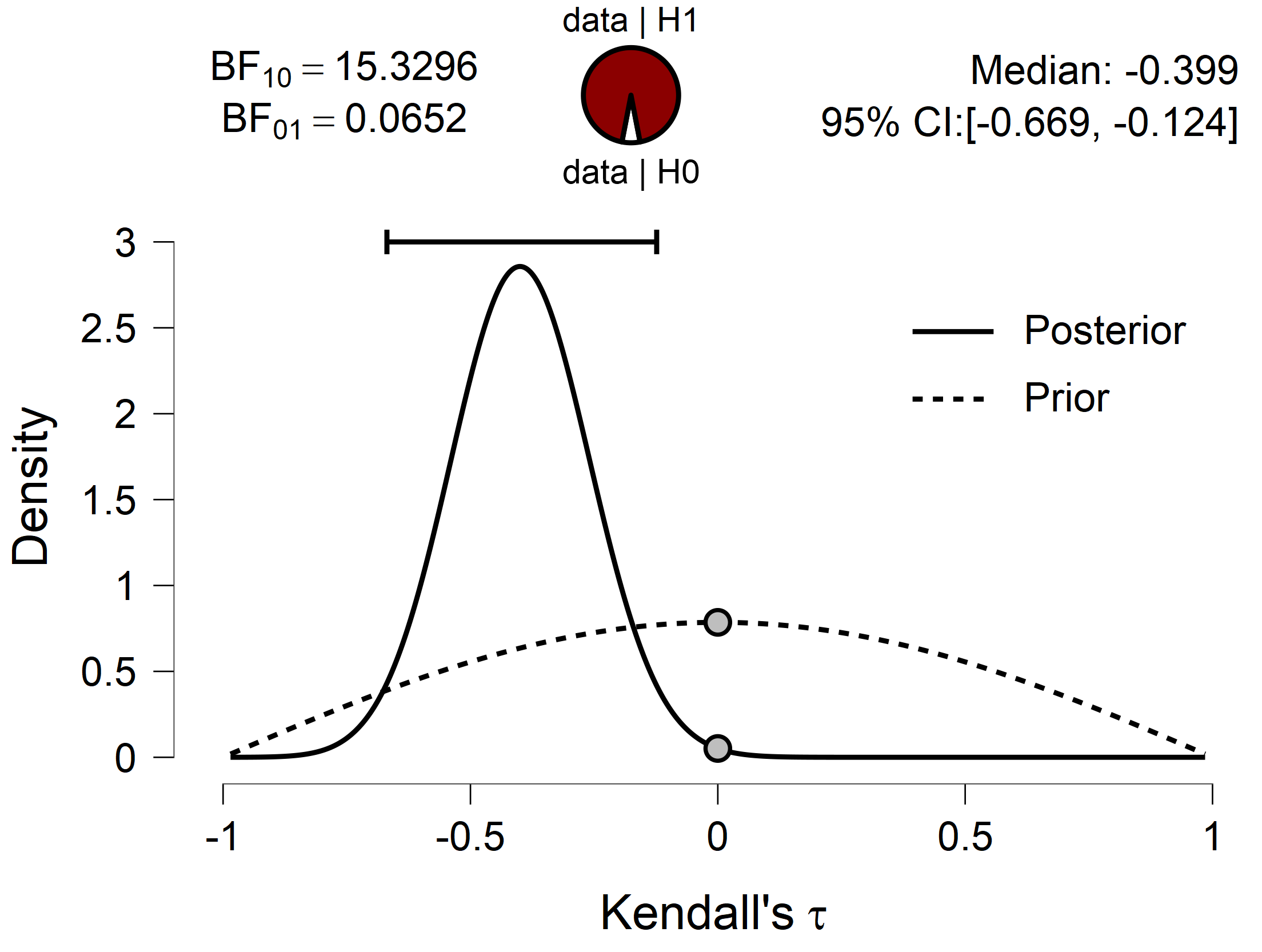


**Supplementary Figure 42.** Prior and posterior distribution of the effect size under H1 setting a default prior.

## Percentage of Investment S2 – PAA S2


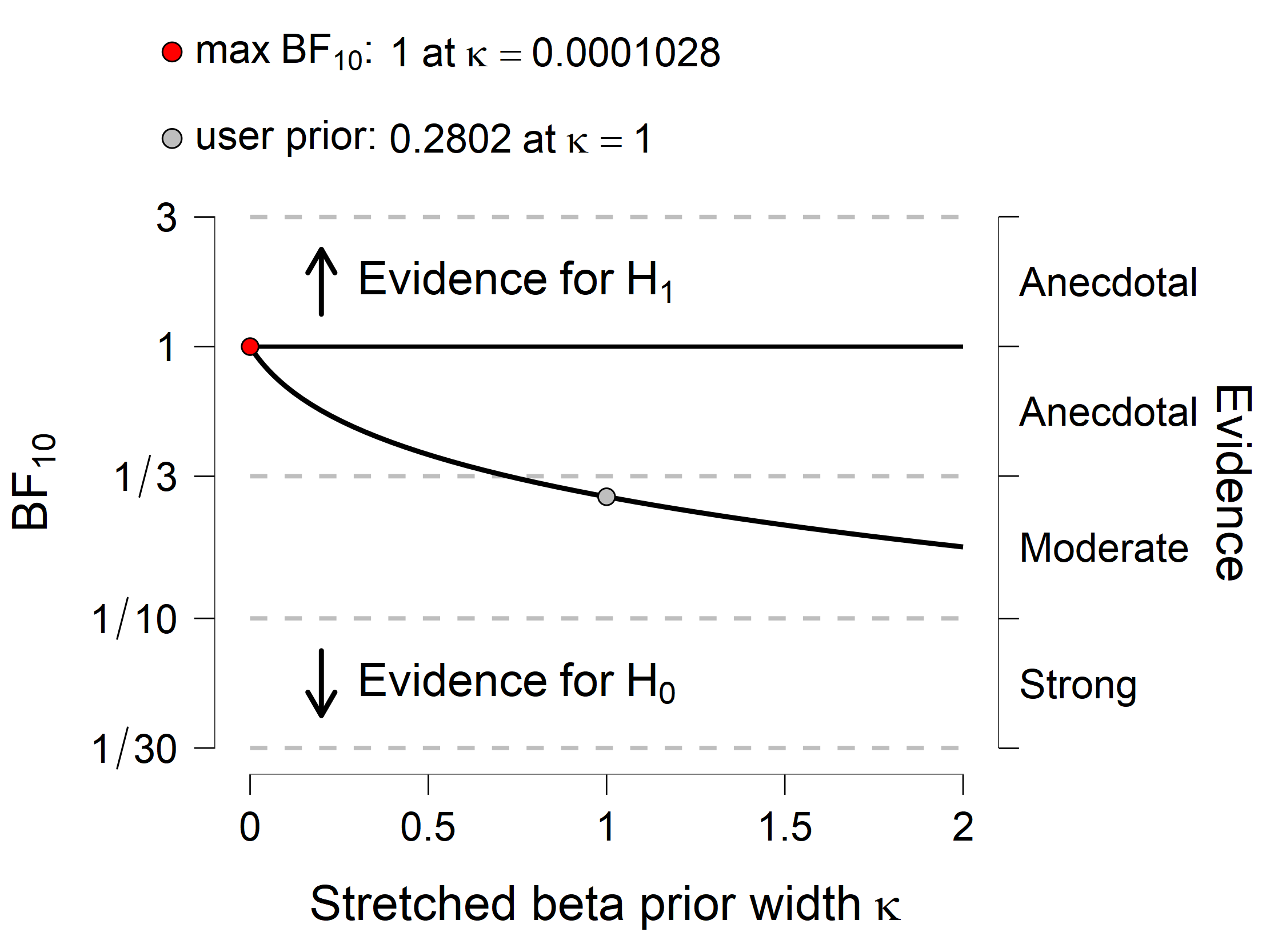


**Supplementary Figure 43.** Robustness analysis for the BF_10_ when correlating the percentage of investment in S2 with the PAA in S2. Maximum BF_10_ in red and Default BF_10_ in grey.


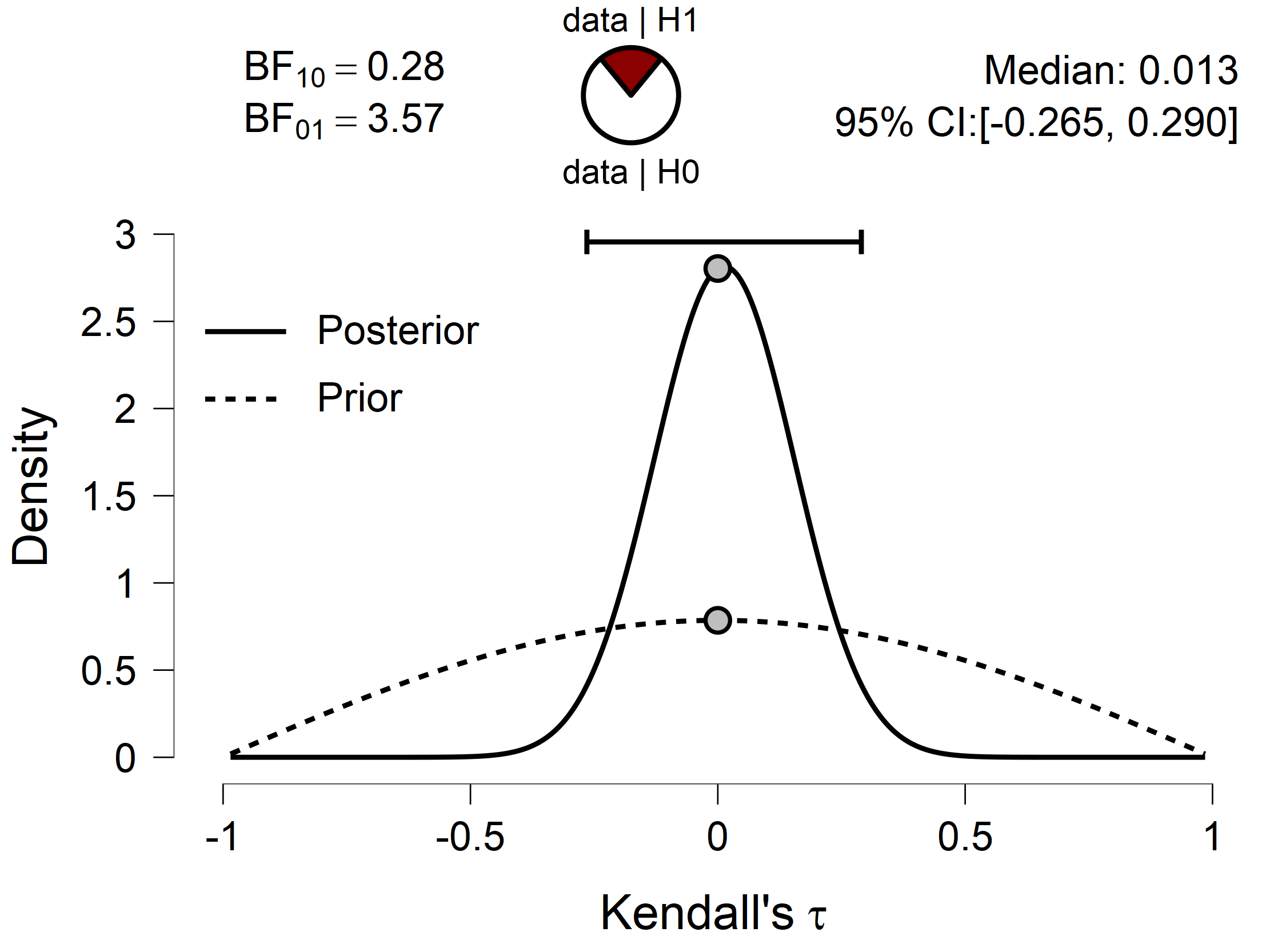


**Supplementary Figure 44.** Prior and posterior distribution of the effect size under H1 setting a default prior.

## Percentage of investment S2 – Pupil Diameter S2


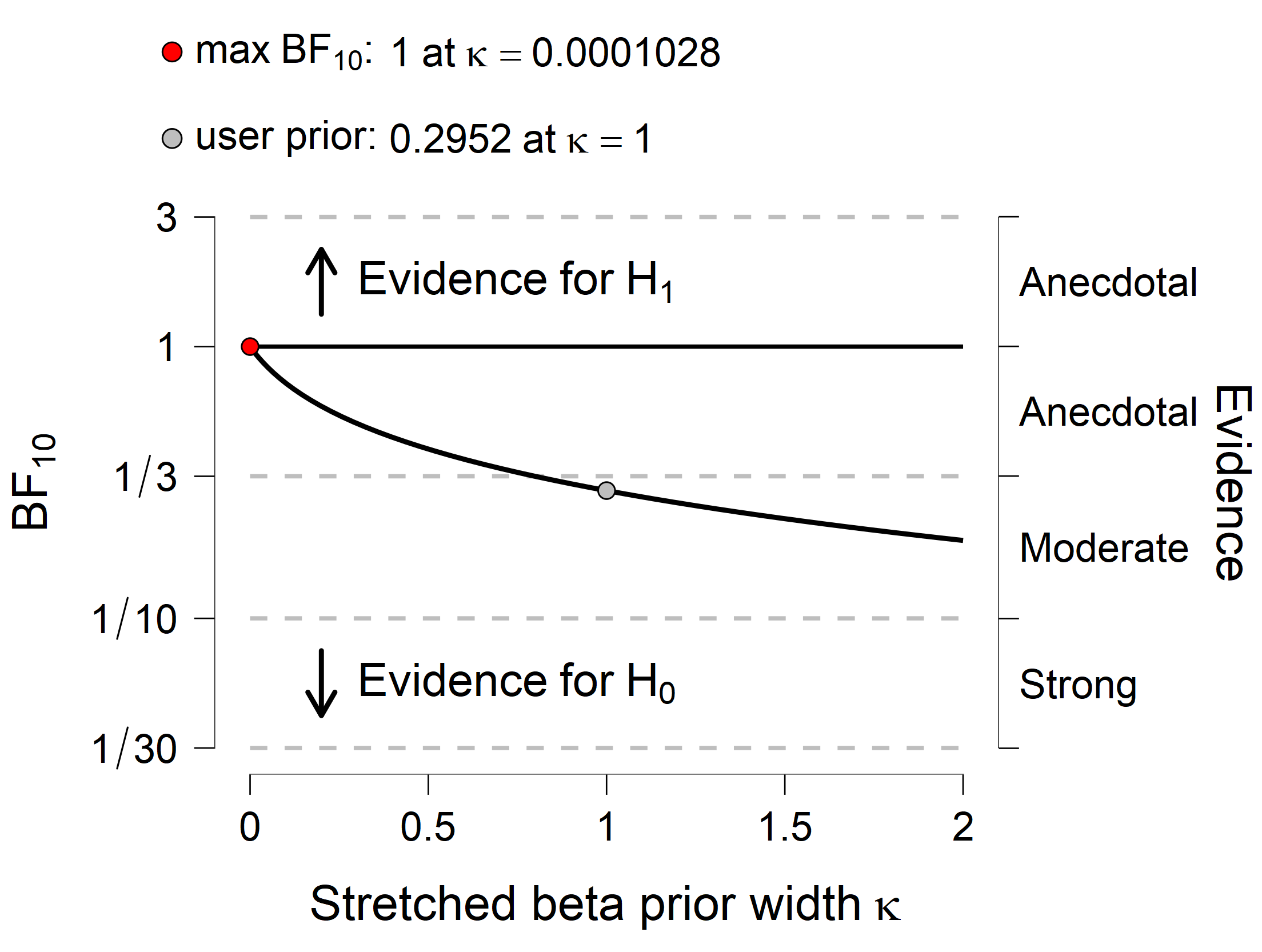


**Supplementary Figure 45.** Robustness analysis for the BF_10_ when correlating the percentage of investment in S2 with the Pupil Diameter in S2. Maximum BF_10_ in red and Default BF_10_ in grey.


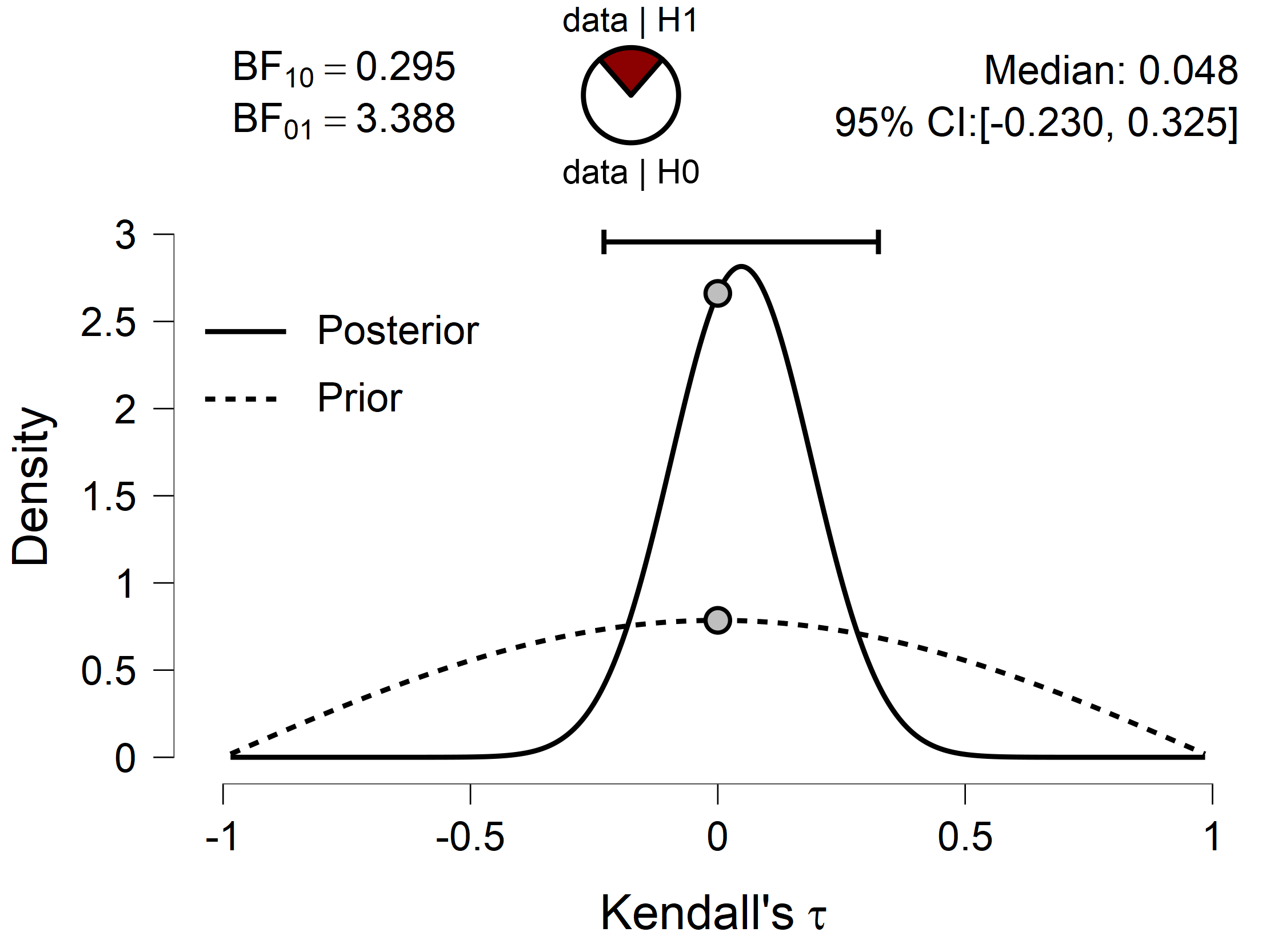


**Supplementary Figure 46.** Prior and posterior distribution of the effect size under H1 setting a default prior.
